# MABGEL 1: First Phase 1 Trial of the Anti-HIV-1 Monoclonal Antibodies 2F5, 4E10 and 2G12 as a Vaginal Microbicide

**DOI:** 10.1371/journal.pone.0116153

**Published:** 2014-12-29

**Authors:** Georgina C. Morris, Rebecca C. Wiggins, Sarah C. Woodhall, J. Martin Bland, Carol R. Taylor, Vicky Jespers, Brigitta A. Vcelar, Charles J. Lacey

**Affiliations:** 1 Centre for Immunology and Infection, Hull York Medical School, University of York, York, United Kingdom; 2 Department of Health Sciences, University of York, York, United Kingdom; 3 Hull York Medical School Experimental Medicine Unit, York Teaching Hospitals NHS Foundation Trust, York, United Kingdom; 4 Institute of Tropical Medicine, Antwerp, Belgium; 5 Polymun Scientific Immunbiologische Forschung GmbH, Vienna, Austria; Burnet Institute, Australia

## Abstract

**Background:**

Monoclonal antibodies (mAbs) which potently neutralize a broad range of HIV isolates are potential microbicide candidates. To date, topical application of mAbs in humans and their stability in vaginal secretions has not been studied.

**Objectives:**

To assess the pharmacokinetics and safety of the mAbs 2F5, 4E10 and 2G12 when applied vaginally in women.

**Design:**

A randomized, double-blind, placebo-controlled phase 1 trial.

**Methods:**

Twenty-eight healthy, sexually abstinent women administered 2.5 g of gel daily for 12 days containing either 10 or 20 mg/g of each mAb (MABGEL) or placebo. Main clinical evaluations and sampling occurred at baseline, 1, 8, and 24 hours post-1^st^ dose and 12 and 36 hours post-12^th^ dose.

**Results:**

After adjustment for dilution factors, median levels of 2F5, 4E10 and 2G12 in vaginal secretions at 1 hour post high-dose MABGEL were 7.74, 5.28 and 7.48 mg/ml respectively. Levels of 2F5 and 4E10 declined exponentially thereafter with similar estimated half-lives (4.6 and 4.3 hours). In contrast, 2G12 levels declined more rapidly in the first 8 hours, with an estimated half-life of 1.4 hours during this period. There was no evidence of systemic absorption. There were no significant differences in local or systemic adverse event rates or vaginal flora changes (by qPCR) between active and placebo gel arms. Whilst at least 1 adverse event was recorded in 96% of participants, 95% were mild and none were serious.

**Conclusions:**

Vaginal application of 50 mg of each mAb daily was safe over a 12 day period. Median mAb concentrations detected at 8 hours post dose were potentially sufficient to block HIV transmission.2G12 exhibited more rapid elimination from the human vagina than 4E10 and 2F5, likely due to poor stability of 2G12 in acidic human vaginal secretions. Further research is needed to develop mAb-based vaginal microbicides and delivery systems.

**Trial Registration:**

ISRCTN 64808733 UK CRN Portfolio 6470

## Introduction

Women remain disproportionately affected by the HIV-1 pandemic. In sub-Saharan Africa, where heterosexual intercourse is the primary route of transmission, women constitute approximately 60% of adults living with HIV infection. Of those with HIV aged 15 to 24 years around 85% are female [1]. There have been significant recent advances concerning the use of anti-retrovirals (ARVs) in HIV prevention. Within discordant heterosexual partnerships, providing combination ARVs as treatment for the HIV positive partner [2] or Truvada (tenofovir plus emtricitabine, Gilead, Foster City, CA, USA) as pre-exposure prophylaxis (PrEP) for the negative partner [3], reduced within-partnership transmissions to women by 96% and 66% respectively. However, studies of oral PrEP in women who are unaware of their partner's HIV status have shown discordant findings [4], [5], [6]. Proof of concept of the efficacy of an ARV microbicide to prevent HIV-1 transmission was demonstrated by the CAPRISA 004 trial, in which a 1% tenofovir gel used before and after sex gave 39% protection overall [7]. Efficacy increased in proportion with dosing adherence (confirmed by pharmacokinetic analyses), with 54% protection achieved with gel use in over 80% of vaginal sex acts. Recent disappointing results from the VOICE Trial (which compared daily use of one of 3 interventions- oral Truvada, oral tenofovir or 1% tenofovir vaginal gel, but showed that none of these strategies was protective due to low adherence [6]) have further emphasised the need to develop products that are acceptable to women and fit in with their lifestyles. As with contraception, it is unlikely that one product or strategy will suit all women and use will be influenced by a range of factors, including stability of relationships, perception of need, and any adverse effects. Less-than-daily dosing schedules, such as pre- or peri-coitally, or long-acting delivery mechanisms, e.g. rings or injections, may prove more favourable to some women than daily interventions.

Despite the undoubted potential of ARVs as PrEP, there remain concerns that topical ARVs or incomplete adherence to oral ARV dosing could give rise to resistance mutations in users who acquire HIV. Efficacy may also be reduced in the presence of ARV-resistant HIV strains. Thus, development of non-ARV-based anti-HIV microbicides remains a priority.

Monoclonal antibodies (mAbs) have been identified which potently neutralize a broad range of HIV isolates [8]–[10]. Some of the best characterised of these are 2F5, 4E10 and 2G12. 2F5 and 4E10 bind to neighbouring epitopes ELDKWA and NWFDIT on the gp41 membrane proximal external region (MPER) [11], whereas 2G12 binds to a cluster of carbohydrate residues on the gp120 glycan shield [12]. Intravenous (IV) passive transfer of 2F5, 4E10 and 2G12 has protected macaques following IV, oral, rectal and vaginal simian/human immunodeficiency virus (SHIV) challenge [13]–[16].

In addition to the above animal studies, the mAbs 2F5, 2G12, and 4E10 have been used in passive immunotherapy studies in humans. 4 studies involving a total of 39 HIV-1 positive individuals have been conducted [17]–[20]. Subjects received a dose of each mAb, ranging from 1 to 5g per infusion, at weekly intervals for between 4 and 16 weeks. Overall, the mAbs were shown to be safe, with no serious adverse events or thrombotic complications reported. Mild symptoms of myalgia, arthralgia and rash were seen in 8/24, 5/24 and 2/24 of subjects, respectively, receiving between 42 and 48 g of mAb over 13 to 16 infusions in the latter 2 studies [21]. In these studies, where all participants had stored, pre- HAART viral isolates which were sensitive to neutralization by at least 2 of the mAbs (all 3 mAbs in the majority), some efficacy in temporarily delaying viral rebound after cessation of HAART was observed in 14/16 individuals who had initiated HAART during acute/early HIV-1 infection, with 4 maintaining VL suppression to <50 copies/ml during the entire infusion period. Viral mutants emerged during rebound which were resistant to 2G12 in 19/22 participants, providing additional evidence that it produced a neutralizing effect causing a selective pressure [19], [20].

Although the generation of individual broadly-neutralising mAbs during chronic HIV-1 infection is more common than previously appreciated, antibodies against the MPER region are relatively rare [22]–[24], with most broadly-neutralising mAbs targeting gp120 [25]. It has been suggested, therefore, that they may arise as a result of atypical B cell induction pathways and potentially bind host (‘self’) epitopes [23], [26], [27]. Initially it was shown that both 2F5 and 4E10, but not 2G12, displayed auto-reactivity *in-vitro* through binding to various phospholipids, histones and other auto-antigens [26]. Further analyses and experiments in relation to the human clinical trials were thus performed to investigate the possibility of *in-vivo* autoreactivity and possible thromboembolic disorders associated with 4E10, 2F5 and 2G12. During the last trial the study design was modified to collect prospective data from the last four high-dose patients. Very mild (<1.25× ULN) prolongation of the activated partial thromboplastin time (APTT) 30 minutes post-infusion was observed which rapidly remitted, but there was no effect on the prothrombin time (PT) [21]. *In-vitro* experiments indicated that the effect on coagulation profile was mediated by 4E10. Further analyses have indicated that 4E10, but not 2F5 or 2G12, shows low-level cross-reactivity with cardiolipin, and that infusion of 4E10 resulted in transient low anti-cardiolipin antibody titres [21], [28]. Similar transient rises in anti-cardiolipin antibodies are a recognised feature of some viral infections, including HIV-1, and are rarely pathogenic, in contrast with the ‘classical’ anticardiolipin associated with autoimmune diseases such as systemic lupus erythematosus [29]–[32]. It was concluded that there was little evidence that the mAbs conferred an increased risk of thrombosis or other autoimmune phenomena, but that monitoring of both coagulation parameters and anti-cardiolipin antibodies (aCL) should be carried out in any further clinical trials [21].

A vaginal microbicide containing a combination of 4E10, 2F5 and 2G12 (MABGEL) was developed by the European Microbicides Programme (EMPRO). We therefore carried out a phase 1 trial (MABGEL1) to evaluate the pharmacokinetics and safety of MABGEL in healthy female volunteers. We also further investigated the vaginal half-lives of the mAbs in an ex-vivo stability study. As well as being the first investigators to study the topical application of mAbs within the human female genital tract, we are also among the first to employ quantitative molecular techniques [33] to determine the effects of a microbicide product on the vaginal microflora [34], [35]. Given that even subtle perturbations of vaginal flora can potentially increase the risk of HIV acquisition [36]–[38], accurate quantification and identification of bacteria at a species level may prove a useful addition to the safety assessments currently employed in trials of vaginal products [39]. Quantitative, DNA-based methods (amplification of the 16SrRNA genes) have an advantage over culture in that they can provide an accurate assessment of a wide range of non-fastidious and fastidious species, using stored swabs, rather than fresh samples.

## Materials and Methods

The MABGEL1 protocol and supporting CONSORT checklist are available as supporting information; see Checklist S1 and Protocol S1. Results have been reported in compliance with the CONSORT 2010 recommendations (www.consort-statement.org) [40], [41].

### Ethics Statement, Study Approval and Registration

MABGEL 1 was approved by Cambridgeshire 1 NHS Research Ethics Committee (REC) and conducted in accordance with the principles expressed in the Declaration of Helsinki and the UK Medicines for Human Use (Clinical Trials) Regulations 2004. Fully informed written consent was obtained from all participants prior to any study procedures.

Approval was also obtained from North and East Yorkshire Research and Development Alliance and the UK Medicines and Healthcare products Regulatory Agency. The study was registered on the European and International Clinical Trials Databases: EudraCT (2008-000312-32), ISRCTN (64808733) http://www.isrctn.com/ISRCTN64808733 and on the UK Clinical Research Network Study Portfolio (Study 6470) http://public.ukcrn.org.uk/search/StudyDetail.aspx?StudyID=6470

### MABGEL1: Study Design and Conduct

MABGEL1 was a randomised, double-blind placebo-controlled trial conducted, between September 2009 and July 2010, in the Hull-York Medical School Experimental Medicine Unit, York Hospital, UK. The primary objective of the study was to assess the local pharmacokinetics of 2F5, 4E10 and 2G12 when applied vaginally and, in addition, to determine whether there was any evidence of systemic absorption of the mAbs. Assessment of the local and systemic safety of the mAbs when applied vaginally was the secondary study objective.

Including screening, evaluations were conducted over 8 visits, and 2 follow-up telephone calls, spanning 3 menstrual cycles.

Target recruitment was 30; 10 subjects per study arm. Subject numbers were randomised to MABGEL high dose, MABGEL low dose or gel vehicle alone in the ratio 1∶1∶1 using blocked randomisation of mixed block sizes. Randomisation was performed by the York Trials Unit, University of York. The randomisation programme was created using Microsoft Visual Basic, and a copy of the data generated was stored securely on a password-protected database. The randomisation list was sent securely by York Trials Unit to a Qualified Person at Polymun Scientific, Austria for the purposes of labelling the study gel, after which it was destroyed. Personnel carrying out subsequent analyses did not have access to this list. Participants received sequential subject numbers in order of enrolment. All participants, study staff, pharmacists, clinicians and statisticians were blinded to study assignments. Double-blinding remained in place throughout the study.

### Recruitment and Selection of Participants

Recruitment, consent and enrolment processes were conducted by study clinicians as per the protocol approved by the REC. Women were eligible for the study if they were 18 to 45 years of age, HIV negative, in good general health, not pregnant, breast feeding, or within 12 weeks post-partum, using a reliable method of contraception (as defined in the study protocol i.e. consistent use of condoms, combined oral contraceptive (COCP), Desogestrel-containing progestagen only oral contraceptive (POP), intrauterine device, injectable or implanted progestagen-based contraceptive), and with no clinically significant abnormalities on baseline blood tests. At the screening visit, a thorough medical history and physical examination were conducted. Evaluation of the lower genital tract included colposcopy and digital image photography, and screening for genital tract infections (*Chlamydia trachomatis* (CT), *Neisseria gonorrhoea*, *Trichomonas vaginalis*, Candida *species, bacterial vaginosis* (BV)). Blood tests included haematology, biochemistry, coagulation parameters (activated partial thromboplastin time (aPTT), prothrombin time (PT)), HIV, Syphilis, HBV, HCV serology and IgG anti-cardiolipin (aCL) antibodies [21].

### Investigational Products and Dosing Procedures

mAbs were expressed in Chinese hamster ovary (CHO) cells as human IgG1 (k) and manufactured by Polymun Scientific, Vienna, Austria. Gels, similar in appearance, contained 20 mg/g (MABGEL high dose), or 10 mg/g (MABGEL low dose) of each of the mAbs in a vehicle of hydroxyethylcellulose (1.6%), glycerin (2.5%), methylparaben (0.18%), propylparaben (0.02%), maltose (5 to 5.2%) and purified water (to 100%). Individual 2.5 ml doses were transferred from syringes and administered using single-use Ortho vaginal applicators. Participants commenced using the gels between days 7 and 13 of their menstrual cycle. The first dose was administered in the Experimental Medicine Unit under the supervision of the study physician. The subsequent 11 doses were applied at home by participants on successive days. The 1st dose was applied in the early morning, and the 12^th^ at a pre-arranged time in the evening. Compliance with gel use was assessed by history, diary cards and by inspection of returned syringes.

Participants remained seated in the clinic room for 1 hour post 1^st^ dose. There were no other ambulatory restrictions. Participants were required to be sexually abstinent and refrain from using tampons and vaginal products from 48 hours prior to the 1^st^ dose to 36 hours post the 12^th^ dose.

### Pharmacokinetic Evaluations

Cervico-vaginal samples were obtained just prior to the 1st dose, and at 1 hour, 8 hours and 24 hours post-1st dose, and 12 and 36 hours post-12th dose. Two samples were taken per participant at each time point using Weck-Cel sponges (Medtronic, Watford, Hertfordshire, UK), which were each held against the vaginal mucosa at the lateral fornix for 1 minute, and weighed pre- and post-sample collection. Processing and storage were as previously described [42]. Serum samples were collected at baseline, 8 hours post-1st dose and 12 hours post-12th dose.

mAb concentrations were determined in vaginal secretions and serum by in-house ELISA at Polymun Scientific, using highly purified internal 2F5, 4E10 and 2G12 antibodies as standards and previously described methodology [19]. The lower limit of detection of the mAb assays was 0.06 µg/ml. Values <0.06 µg/ml were reported as not detected (ND). The mAb levels in vaginal secretions were adjusted to account for the dilution factor introduced by the addition of buffer during processing of the Weck-Cel samples, calculated as (Extraction buffer volume (600 µl) + secretion volume/secretion volume). Weights of individual Weck-Cel samples were converted to volumes by assuming a density of 1 mg/ µl. The eluates of the two Weck-Cel samples taken per participant per visit were pooled for analysis by ELISA, and dilution factors calculated using the mean of the two sample volumes. In view of reported cross-reactive binding of 2G12 by *Candida albicans* and *C. tropicalis* [43], vaginal Candida cultures (Sabouraud dextrose agar plus chloramphenicol, Oxoid, Basingstoke, Hampshire, UK); and speciation (API 20 C AUX, bioMerieux, Basingstoke, Hampshire, UK) were performed at entry.

### Safety Evaluations

Participants were monitored for adverse events (AEs) and compliance at each pharmacokinetic sampling time-point. Additional safety and compliance checks were also conducted at 2, 5 and 8 days post 1st dose. The final follow up evaluation took place 10 to 28 days after completion of dosing.

Genital AEs were solicited using questions related to unexpected vaginal bleeding, genital discomfort (itching, burning, soreness), discharge, dysuria and dyspareunia. Colposcopy and image capture was performed prior to sampling. Colposcopy was conducted in general accordance with the CONRAD/WHO Manual for the Standardization of Colposcopy for the Evaluation of Vaginal Products (2004) [44], however no saline lavages or swabs were used apart from the study samples specified in the protocol to avoid impacting on pharmacokinetic evaluations.

Vaginal flora was monitored through on-site microscopy (using Nugent scoring [45]) and through determination of Lactobacilli and BV-associated organisms via quantitative polymerase chain reaction (qPCR) (performed at the Institute of Tropical Medicine (ITM) Antwerp, Belgium). High vaginal samples were obtained, at baseline (screening visit), 36 hours post 12^th^ dose and at the final evaluation visit, using flocked Dacron swabs (Copan, Italy) (stored at −20°C pre-processing). DNA extraction and qPCR methods have been described in detail previously [33]. PCR was performed using SYBR Green PCR Mastermix (Qiagen, Venlo, the Netherlands) on 5 µl samples of extracted DNA. Specific primers were selected to detect Lactobacilli generally and also individual species (*Lactobacillus crispatus*, *L. gasseri*, *L. jensenii, L. vaginalis* and *L. iners*) and the BV-associated organisms *Gardnerella vaginalis* and *Atopobium vaginae*. Primers (synthesised by Eurogentec, Seraing, Belgium) were designed in-house for *L. vaginalis* [33] but have been described elsewhere for the other L. species, generic Lactobacilli, *G. vaginalis and A. vaginae* [46]–[49].

Blood tests for aCl antibodies, clotting profile, haematology and biochemistry were performed throughout the study (by York Hospital Pathology Laboratories in accordance with their standard protocols).

AEs were graded according to tables devised by the UK Medical Research Council Clinical Trials Unit Microbicides Development Programme (genital) [50] and the US National Institute of Health Division of AIDS (Version 1.0, December 2004) (all others) [51]. Causality was assessed as related to study gel (definitely, probably or possibly related) or not related (probably not related, unrelated).

### Statistical Analyses

All statistical analyses were performed on an intention to treat basis using STATA version SE 10.1. mAb concentrations were summarised at each time-point for each treatment group (high dose MABGEL, low dose MABGEL and placebo) as minimum, median, and maximum. Treatment arms were compared using the Kruskal-Wallis rank test corrected for ties, and where this was statistically significant, P<0.05, the two active MABGEL arms were compared using the Mann-Whitney U test. Analyses for vaginal samples were performed both before and after adjustment for the dilution factor.

For each type of AE, the number of events experienced as well as number and percentage of participants experiencing the event were tabulated by severity and relationship to treatment for each arm. Total numbers of AEs per participant in each arm were compared using negative binomial regression. Analyses were performed for all events and for events classed as possibly or probably treatment related. The study was not designed with sufficient power for statistical comparisons of rates of different types of AE between the arms.

For each Lactobacillus species, *G. vaginalis and A. vaginae*, presence or absence in women at each time-point by study arm was compared using Fishers Exact test. Absolute counts were compared at each time-point across study arms using Kruskal Wallis rank test, corrected for ties.

### Half- Life (*t_1/2_*) Determination

Estimates of residence vaginal *t_1/2_* for each mAb were derived using mAb concentrations detected at 1, 8 and 24 hours post 1st dose in both MABGEL arms., Plots showing mAb concentrations versus time and log_e_ mAb concentrations versus time were performed for each mAb using data from all participants receiving MABGEL (high and low dose). Assuming exponential decay, an analysis of covariance (ANCOVA) model was fitted to the log transformed data, with time as a continuous predictor and participant as a categorical factor. Two checks on this assumption were performed; first, a time squared term was included and tested to check linearity, then uniformity of the *t_1/2_* was assessed by testing an interaction between time and participant. Provided these assumptions were met, a line could be fitted as log_e_(*y*)  =  *a* – *bt*, where *y* is the antibody level and *t* is time, *a* and *b* are fitted constants. In addition, a factor was included for participant. The half-life, *t_1/2_*, is the time for *y* to be halved or for log_e_(*y*) to fall by log_e_(2). Hence log_e_(2)  =  *bt_1/2_*, *t_1/2_*  =  log_e_(2)/*b*. A 95% confidence interval (CI) can be found from applying the same transformation to the CI for the slope.

### *Ex vivo* mAb Stability Study

Approval was obtained from the Institutional Review Boards of ITM and the University Hospital of Antwerp to collect vaginal secretions from fully consented, healthy asymptomatic women attending a research clinic at ITM for further *in vitro* analyses (See Protocol S2). All women were naturally cycling, had normal vaginal flora (Nugent score 0–3) and were negative on screening for genital tract infections. Vaginal secretions were obtained from eight women by syringe aspiration. Aspirates were pooled (final pH 4.3), and then mixed 1∶1 with 10 mg each per ml of 2F5, 4E10, and 2G12, and incubated for 24 hr at 37°C. Aliquots for testing were removed pre-incubation, and at 0.25, 0.5, 1, 2, 4, 8 & 24 hours. mAb levels were measured at Polymun Scientific using the binding ELISAs described above.

## Results

### Study population, and protocol compliance

**Figure 1:**
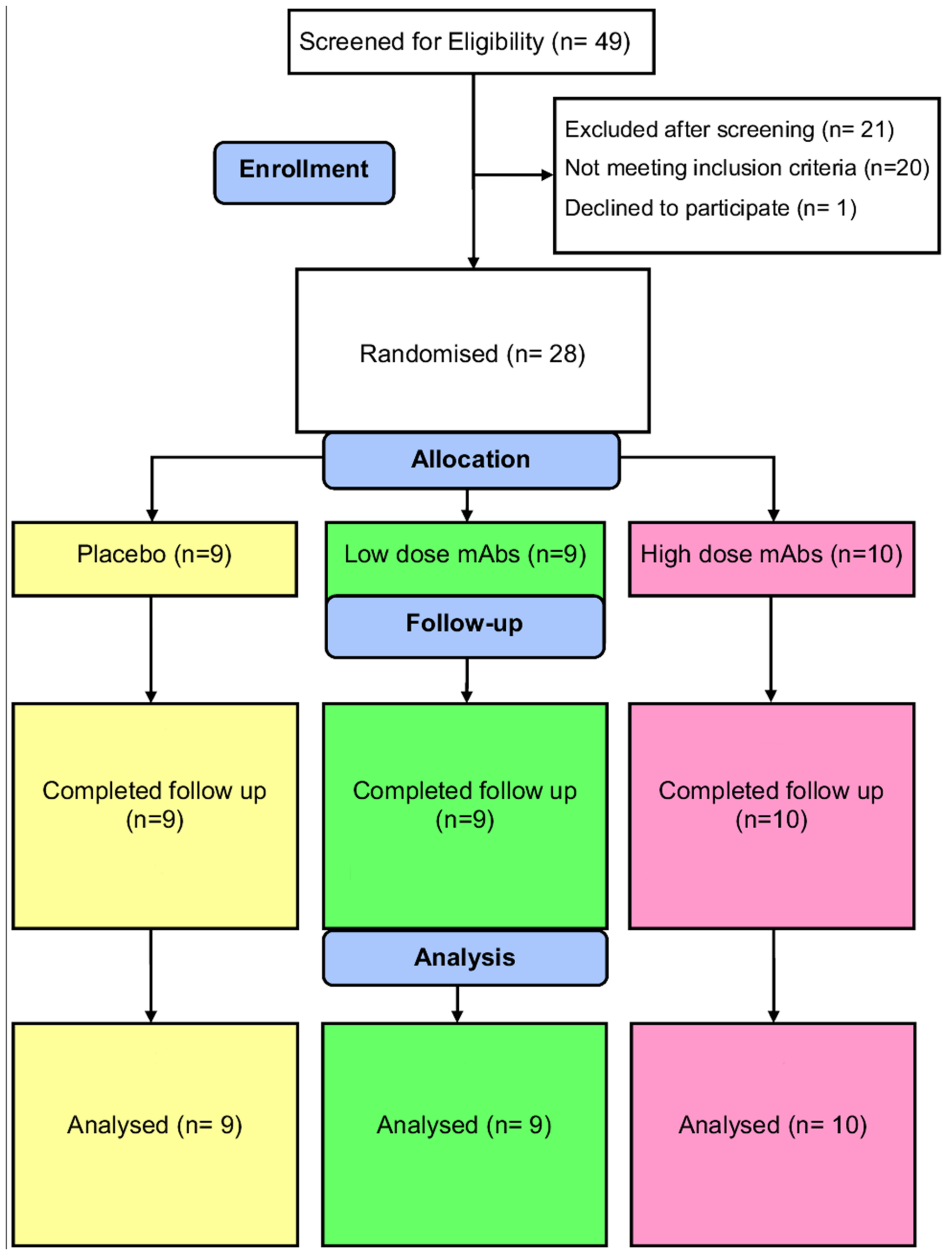
CONSORT diagram of the MABGEL 1 clinical trial.

Forty nine women were screened, and twenty eight women enrolled in the study (Figure 1). Of those excluded at screening, 3 women had genital infections (2× BV, 1× CT); 11 women were judged by the Study Physician and Chief Investigator to have significant current general medical conditions, 1 had deranged liver function tests (alanine transaminase twice upper normal limit), 2 were not using a reliable method of contraception, 1 was judged unlikely to be able to comply with the study protocol (needle phobia), and 2 were not registered with a General Practitioner. 1 eligible woman declined to participate due to starting a new job. The study was terminated early in July 2010 due to expiry of approval of the study gels. Ten women received high dose MABGEL, 9 low dose and 9 placebo. Study arms were well matched generally at enrolment, except women receiving low dose MABGEL were slightly older with higher parity (Table 1). All participants completed follow up. Three women (1 per arm) each missed 1 dose in the middle of the dosing period, and two sets of Weck-cel samples were not analysed; one (in high dose MABGEL arm) due to incorrect dosing timing (participant had mistakenly applied 2^nd^ gel dose just prior to 24 hours post 1^st^ dose visit) and one (placebo arm, 8 hours post 1^st^ dose) discarded in error. All other samples were obtained at specified time-points and analysed, with data presented according to assigned study arm. No sex or vaginal product use was reported by any of the participants.

**Table 1:** Summary of Baseline Characteristics of Enrolled Participants.

		Study Arm		
Demographic	Sub category	High Dose (n = 10)	Low Dose (n = 9)	Placebo (n = 9)
Ethnicity	White British	9 (90%)	8 (89%)	9 (100%)
	White Other	0	1 (11%)	0
	Black African	1 (10%)	0	0
Age (no. in range)	18–24 yrs	4 (40%)	3 (33%)	4 (44%)
	25–34 yrs	6 (60%)	3 (33%)	1 (11%)
	35–44 yrs	0 (0%)	3 (33%)	4 (44%)
Median Age		25	32	25
Mean Height (m)		1.68	1.68	1.68
Mean Weight (kg)		73.2	75.7	62.3
Contraception	Condoms	4 (40%)	5 (56%)	4 (44%)
	IUCD	1 (10%)	0 (0%)	0 (0%)
	COCP	1 (10%)	0 (0%)	3 (33%)
	POP	1 (10%)	0 (0%)	0 (0%)
	IUS	1 (10%)	1 (11%)	0 (0%)
	Implant	2 (20%)	3 (33%)	1 (11%)
	DMPA	0 (0%)	0 (0%)	1 (11%)
No. of (Term) Pregnancies	0	7 (70%)	5 (56%)	6 (67%)
	1	2 (20%)	0 (0%)	1 (11%)
	2	1 (10%)	2 (22%)	1 (22%)
	>2	0 (0%)	2 (22%)	0 (0%)

IUCD  =  copper intra-uterine contraceptive device, IUS  =  intra-uterine system, COCP  =  combined oral contraceptive pill, POP  =  (Desogestrel-containing) progestagen-only pill, DMPA  =  depot medroxyprogesterone acetate.

### Pharmacokinetics

**Figure 2:**
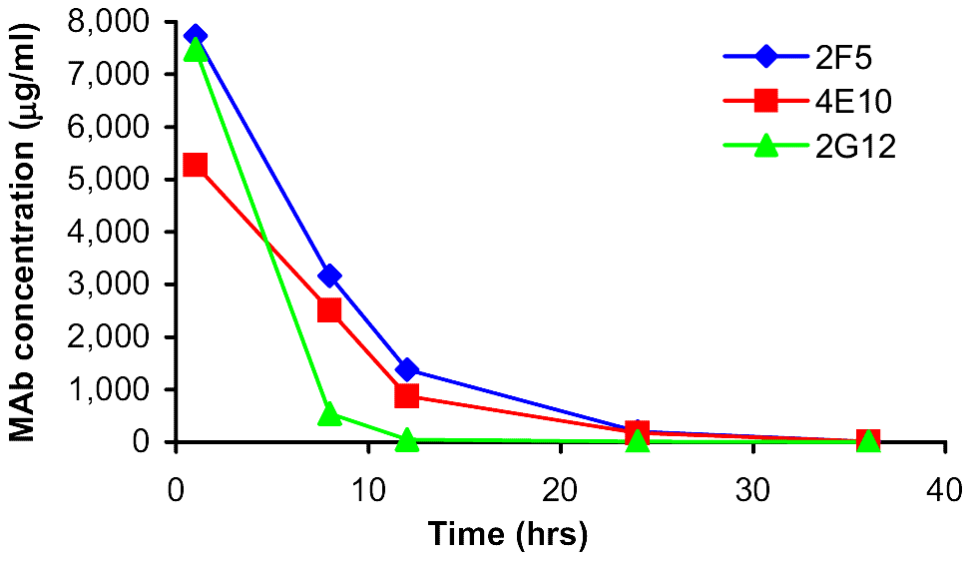
Median concentrations (adjusted for dilution factors) of 2F5, 4E10, 2G12 detected in Weck-cel samples- High dose (20 mg/g) Mabgel.

**Figure 3:**
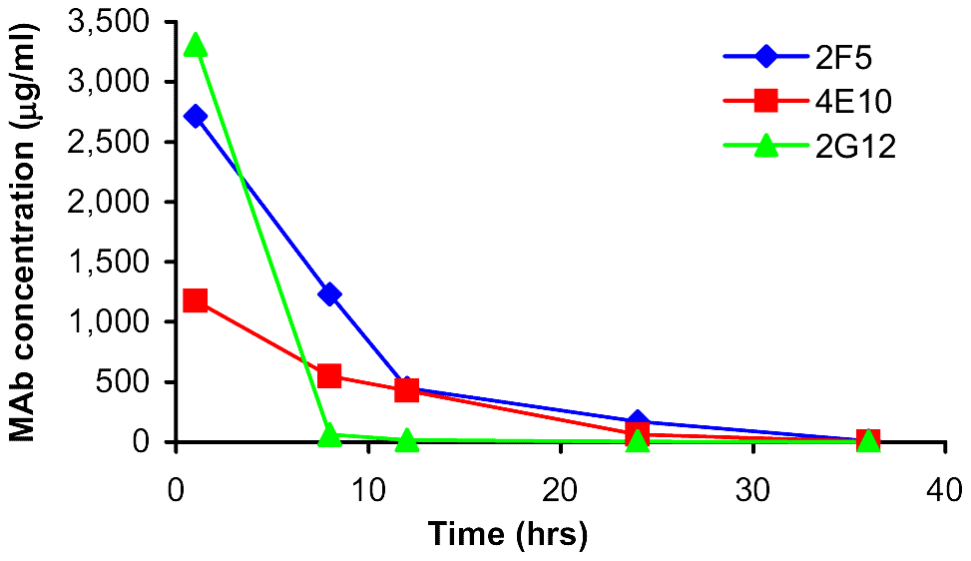
Median concentrations (adjusted for dilution factors) of 2F5, 4E10, 2G12 detected in Weck-cel samples- Low dose (10 mg/g) Mabgel.

As shown in Tables 2 to 4, there were statistically significant differences between the mAb levels detected in the placebo, and in the low dose and high dose MABGEL arms at all time points, except for 2G12 at 36 hrs (post-12th dose) (Kruskal-Wallis). Although there were clearly observable differences between the concentrations detected in the high dose compared with the low dose MABGEL arms at most time-points, these only reached statistical significance at the 1 hour post 1^st^ dose visit for 2F5 and 4E10 and not at all for 2G12 (Mann-Whitney U). These findings were true both before and after adjustment for dilution factors. Median concentrations of 2F5, 4E10, 2G12 in low dose and high dose MABGEL arms (after adjustment for dilution factors) are displayed in Figures 2 and 3.

**Table 2:** Median, minimum and maximum values for cervico-vaginal levels of 2F5 (µg/ml) after adjustment for dilution factors.

	Placebo (n = 9)			Low Dose (n = 9)			High Dose (n = 10)			P values	
Hrs*	Med	Min	Max	Med	Min	Max	Med	Min	Max	All 3 groups	Low vs. High
0 (A)	ND	ND	ND	ND	ND	ND	ND	ND	ND	----	----
1 (A)	ND	ND	3.8	2715	941	6073	7737	903	14377	0.0001	0.009
8 (A)	ND	ND	ND	910	342	3715	3161	30	6102	0.0001	0.06
24 (A)	ND	ND	ND	93	36	530	196	31	729	0.0001	0.4
12 (B)	ND	ND	ND	451	105	2146	1376	41	2533	0.0001	0.5
36 (B)	ND	ND	ND	8.1	ND	161	4.8	ND	118	0.006	0.6

ND: not detected (i.e.<0.06 µg/ml lower limit of assay).

* Hrs post - 1^st^ dose A, 12^th^ dose B.

**Table 3:** Median, minimum and maximum values for cervico-vaginal levels of 4E10 (µg/ml)after adjustment for dilution factors.

	Placebo (n = 9)			Low Dose (n = 9)			High Dose (n = 10)			P values	
Hrs*	Med	Min	Max	Med	Min	Max	Med	Min	Max	All 3 groups	Low vs. High
0 (A)	1.1	ND	5.9	ND	ND	2.0	0.2	ND	1.6	----	----
1 (A)	ND	ND	2.7	1175	583	3849	5277	1442	13580	0.0001	0.002
8 (A)	ND	ND	1.6	547	302	3128	2505	176	6612	0.0001	0.1
24 (A)	ND	ND	1.6	64	ND	297	171	18.2	1088	0.0006	0.2
12 (B)	ND	ND	1.7	427	47	2548	870	28	2198	0.0001	0.7
36 (B)	ND	ND	4.8	5.8	ND	159	4.7	ND	119	0.008	0.8

ND: not detected (i.e.<0.06 µg/ml lower limit of assay).

* Hrs post - 1^st^ dose A, 12^th^ dose B.

**Table 4:** Median, minimum and maximum values for cervico-vaginal levels of 2G12(µg/ml) after adjustment for dilution factors.

	Placebo (n = 9)			Low Dose (n = 9)			High Dose (n = 10)			P values	
Hrs*	Med	Min	Max	Med	Min	Max	Med	Min	Max	All 3 groups	Low vs. High
0 (A)	ND	ND	8.6	ND	ND	6.8	ND	ND	6.7	----	----
1 (A)	ND	ND	3.3	3314	742	12622	7479	1021	27882	0.0001	0.06
8 (A)	ND	ND	3.0	63	19	1183	538	2.4	6953	0.0001	0.06
24 (A)	ND	ND	4.3	5.6	1.1	25	12	1.5	182	0.002	0.2
12 (B)	ND	ND	5.2	17	2.3	283	52	5.4	1982	0.0002	0.8
36 (B)	ND	ND	27.7	1.3	ND	17	ND	ND	160	0.7	----

ND: not detected (i.e.<0.06 µg/ml lower limit of assay).

* Hrs post - 1^st^ dose A, 12^th^ dose B.

There was no evidence of systemic absorption of the mAbs (data not shown). There were no statistically significant differences in the levels of any of the mAbs between the 3 study arms at any time-point and no increases from baseline (pre-dose) values were seen for any of the mAbs.

**Figure 4:**
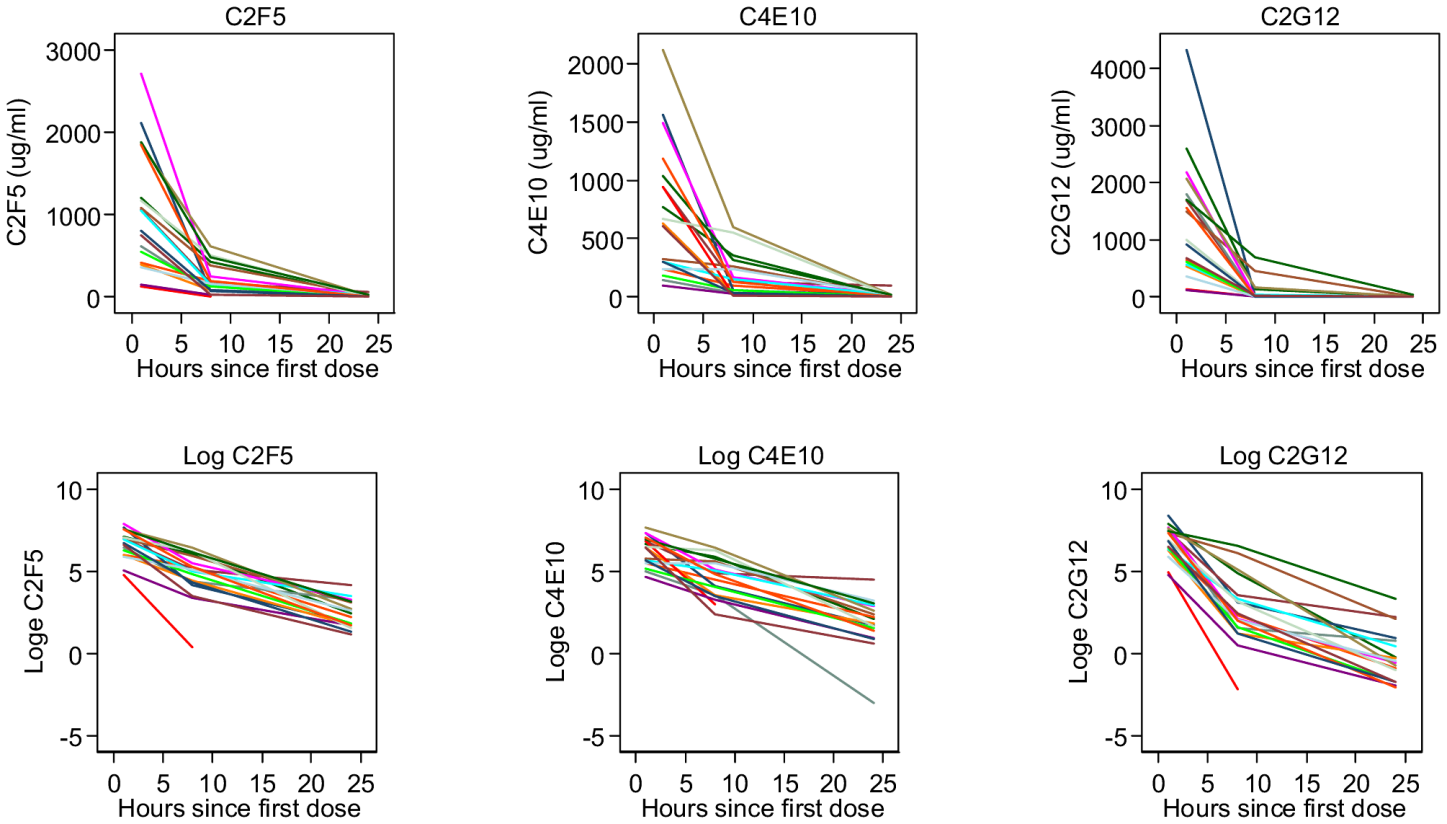
Antibody and log_e_ antibody concentrations over three time points for individual participants given active Mabgel (high and low dose) without adjustment for dilution factors.

**Figure 5:**
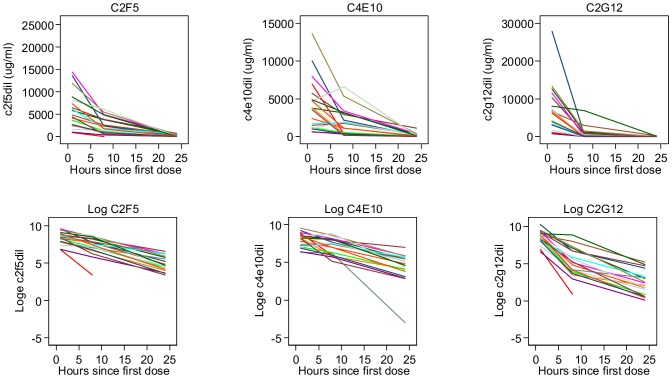
Antibody and log_e_ antibody concentrations over three time points for individual participants given active Mabgel (high and low dose) after adjustment for dilution factors.

Plots showing mAb concentrations and log_e_ mAb concentrations (before and after adjustment for dilution factors) versus time are displayed in Figures 4 and 5. Using the concentrations adjusted for dilution factors, estimates of residence *t_1/2_* in vaginal secretions (Weck-Cel samples) were similar for both of the MPER mAbs (4.3 hours (95% CI 3.6 to 5.3) for 4E10; 4.6 hours (95% CI 4.0 to 5.0) for 2F5) (Figures 2 and 3). *T_1/2_* estimates were approximately 1 hour shorter using the unadjusted values, with both adjusted and unadjusted data fitting an exponential model. In contrast, vaginal levels of 2G12 did not conform to a single overall exponential decay, displaying a more rapid initial rate of decline than the other two mAbs, which then slowed at lower concentrations. An estimate obtained for the early *t_1/2_* of 2G12, based on the 1 and 8 hour post 1^st^ dose values, was 1.4 hours (95% CI 1.2 to 1.8). Note *t_1/2_* estimates given are for the full data set since excluding outliers had limited effects on the estimates.

**Figure 6:**
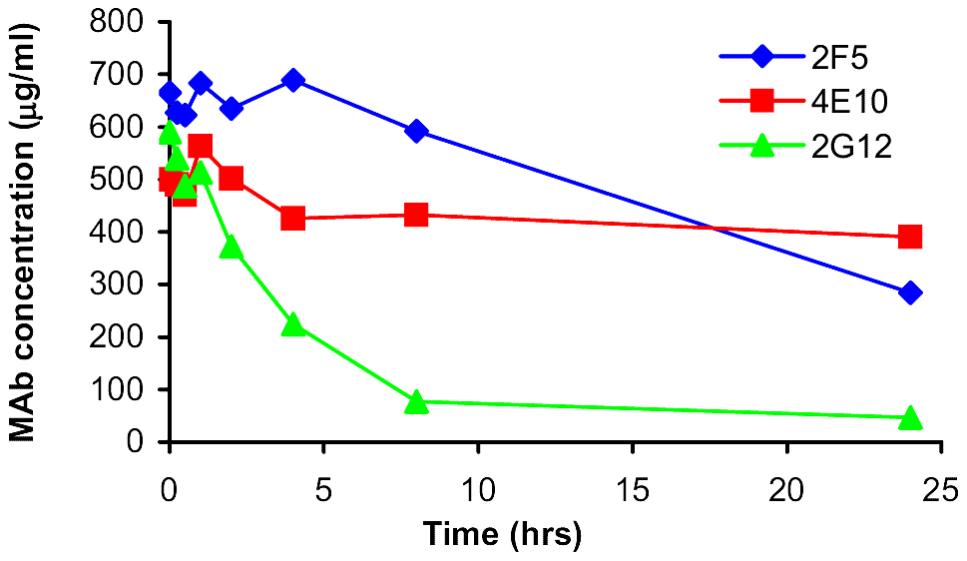
In vitro stability study of 2F5, 4E10, 2G12 in vaginal secretions.

We conducted an *in vitro* stability study to explore the *in vivo* observed accelerated decline of 2G12 in vaginal secretions. The results are shown in Figure 6 and confirm our *in vivo* data. 4E10 showed almost no decline over 24 hrs, 2F5 a slight decline, whereas 2G12 showed a rapid decline with an apparent bi-phasic curve.

### Safety

As displayed in Table 5, AEs were reported by all but 1 participant, however 95% were mild (grade 1). No AEs were serious (grade 3/4) and only 4 were moderate (grade 2). Two of the moderate AEs occurred in a single participant in the placebo arm of the study, the other 2 occurred in 2 separate participants, both in the low dose MABGEL arm. Overall, there were no significant differences in the mean number of AEs recorded per participant between placebo, low-dose and high-dose arms, and this was true for all events (P = 0.6) (negative binomial), and also the 88% of events considered at least possibly related to gel use (i.e. adverse reactions (ARs) (P = 0.1).

**Table 5:** Distribution and Nature of Adverse Events (AEs).

					Overall Number of AEs (%)				
		Number of participants experiencing AE (%) per study arm			Severity Grading			Relatedness to Gel Use	
Nature of AE		Placebo (n = 9)	Low dose Mabgel (n = 9)	High dose Mabgel (n = 10)	1	2	≥3	At Least Possibly Related	Not Related
Total AEs (n = 88)					84 (95)	4 (5)	0 (0)	77 (88)	11 (13)
At least 1 AE		9 (100)	9 (100)	9 (90)					
Reproductive system	Non-menstrual bleeding	6 (67)	5 (56)	2 (20)	11 (85)	2 (15)	0 (0)	13 (100)	0 (0)
	Discomfort (soreness, burning, itch)	6 (67)	2 (22)	1 (10)	10 (91)	1 (9)	0 (0)	11 (100)	0 (0)
	Vaginal discharge	3 (33)	2 (22)	2 (20)	10 (100)	0 (0)	0 (0)	10 (100)	0 (0)
	Vulval or vaginal erythema	1 (11)	2 (22)	0 (0)	3 (100)	0 (0)	0 (0)	3 (100)	0 (0)
	Epithelial disruption	1 (11)	0 (0)	0 (0)	1 (100)	0 (0)	0 (0)	1 (100)	0 (0)
Gastrointestinal	Abdominal pain (lower)	2 (22)	1 (11)	2 (20)	6 (100)	0 (0)	0 (0)	6 (100)	0 (0)
	Abdominal pain (upper)	0 (0)	1 (11)	0 (0)	3 (100)	0 (0)	0 (0)	3 (100)	0 (0)
	Nausea, bloating, vomiting or diarrhoea	0 (0)	1 (11)	2 (20)	4 (100)	0 (0)	0 (0)	4 (100)	0 (0)
Haematological	Neutropenia	1 (11)	1 (11)	1 (10)	3 (100)	0 (0)	0 (0)	3 (100)	0 (0)
	Low total white cell count	1 (11)	0 (0)	1 (10)	2 (100)	0 (0)	0 (0)	2 (100)	0 (0)
	Increased prothrombin time	3 (33)	0 (0)	3 (30)	8 (100)	0 (0)	0 (0)	4 (50)	4 (50)
Biochemical	Increased blood bilirubin	0 (0)	0 (0)	1 (10)	1 (100)	0 (0)	0 (0)	1 (100)	0 (0)
	Increased blood creatinine	0 (0)	1 (11)	0 (0)	1 (100)	0 (0)	0 (0)	1 (100)	0 (0)
Infections	Candida (pre 1^st^ dose)	1 (11)	1(11)	1 (11)	3 (100)	0 (0)	0 (0)	0 (0)	3 (100)
	Bacterial vaginosis	0 (0)	1 (11)	0 (0)	1 (100)	0 (0)	0 (0)	1 (100)	0 (0)
Other Clinical	Lethargy	1 (11)	1 (11)	0 (0)	2 (67)	1 (33)	0 (0)	2 (67)	1 (33)
	Headache	4 (44)	3 (33)	1 (10)	8 (100)	0 (0)	0 (0)	7 (88)	1 (13)
	Back Pain	1 (11)	1 (11)	0 (0)	4 (100)	0 (0)	0 (0)	3 (75)	1 (25)
	Rash	0 (0)	0 (0)	1 (10)	1 (100)	0 (0)	0 (0)	1 (0)	0 (0)
	Other	0 (0)	1 (11)	1 (10)	2 (100)	0 (0)	0 (0)	1 (50)	1 (50)

51% of reported ARs involved the genital tract, including unexpected vaginal bleeding, abnormal vaginal discharge, and vaginal discomfort. Of these, vaginal bleeding was the most common, reported by 46% (13/28) of participants. Examination findings suggestive of possible mucosal inflammation were seen in 5 participants. There were 4 cases of erythema (none in high dose, 3 in low dose, 1 in placebo arm) and 1 case of superficial epithelial disruption (<1 swab tip) (placebo arm); all were mild and localised. One of the cases of erythema, associated with soreness, was attributed to *C. albicans* (detected on pre-1^st^ dose culture) and treated with fluconazole; all others were self-limiting. Only 2 AEs were seen on colposcopy that would possibly not have been detected by participant report or visual examination with the naked eye alone. 1 participant had a small area of erythema on her posterior vaginal wall that was not seen with the naked eye. The single superficial epithelial disruption was just visible with the naked eye but better delineated using the colposcope. Both of these findings were detected at visit 3 (8 hours post 1^st^ dose) but had resolved by visit 4 (24 hours post 1^st^ dose). No genital findings were present at more than 1 successive evaluation, although all were attributed as possibly related to use of the study gel. Overall, there were more genital ARs in placebo users (17) than in the MABGEL arms (9 high dose, and 13 low dose).

All laboratory ARs were grade 1 and none required any medical intervention. All aCL titres and aPTT values during the study were in the laboratory normal ranges. 6 participants (3 placebo arm, 3 high dose MABGEL arm) had transiently mildly elevated PT results (<1.25× upper limit of normal) detected during the study. In 2 of these participants (1 placebo, 1 high dose), PT results were elevated on Visit 2 (pre-1st dose) and Visit 8 samples but not in between. These were considered to be unrelated to study gel use by the Chief Investigator as only elevated before and several weeks after gel exposure.

There was no evidence that either MABGEL or placebo caused a change in vaginal flora. Lactobacilli were detected in all women at all visits using the generic PCR primers. *A. Vaginae* and *G. vaginalis* were only present in a minority of women. In general, the presence or absence of a particular *Lactobacillus* species or BV-associated bacterial species appeared to remain constant throughout the study (Table 6.)

**Table 6:** Presence of *Lactobacillus* species, *Gardnerella vaginalis* and *Apotobium vaginae* by visit and study arm.

			L. crispatus	L. iners	L. jensenii	L. gasseri	G. vaginalis	A. vaginae	Median Nugent Score	Vaginal pH	
										Median	IQR
Visit	Arm	n	n (%)	n (%)	n (%)	n (%)	n (%)	n (%)			
Screening	Placebo	9	5 (56)	7 (78)	7 (78)	4 (44)	2 (22)	3 (33)	0	4.3	0.3 [4.15–4.45]
	Low dose	9	8 (89)	2 (22)	7 (78)	3 (33)	2 (22)	3 (33)	0	4.3	0.3 [4.3–4.6]
	High dose	10	7 (70)	5 (50)	7 (70)	1 (10)	1 (10)	0 (0)	0	4.3	0.45 [4.3–4.75]
	P-value *		0.343	0.080	1.00	0.256	0.703	0.099			
36hrs PLD	Placebo	9	6 (67)	6 (67)	7 (78)	4(44)	2 (22)	1 (11)	0	4.3	0.3 [4.3–4.6]
	Low dose	9	8 (89)	2 (22)	7 (78)	4 (44)	1 (11)	2 (22)	0	4.3	0.3 [4.3–4.6]
	High dose	10	7 (70)	5 (50)	7 (70)	3 (30)	0 (0)	0 (0)	0	4.3	0.60 [4.3–4.9]
	P-value *		0.639	0.183	1.00	0.793	0.286	0.286			
Final FU	Placebo	9	6 (67)	6 (67)	7 (78)	3 (33)	4 (44)	2 (22)	0	4.3	0.3 [4.3–4.6]
	Low dose	9	8 (89)	2 (22)	7 (78)	4 (44)	3 (33)	2 (22)	0	4.3	0.3 [4.3–4.6]
	High dose	10	8 (80)	5 (50)	6 (60)	3 (30)	2 (20)	1 (10)	0	4.3	0.60 [4.0–4.6]
	P-value *		0.639	0.161	0.664	0.887	0.536	0.703			

PLD: Post-last dose, IQR: Interquartile range.

In keeping with the previous analysis, the only time point and *Lactobacillus* species where there was a significant difference in counts detected between the study arms was at screening for *L. iners* (p = 0.04) (Kruskal-Wallis). Overall, no significant changes in any Lactobacillus or BV-associated bacterial species were detected from baseline at either 36 hours post 12^th^ dose or final visit in any study arm as quantified by qPCR (data not shown). Only one woman, in the low dose Mabgel arm, developed BV during the study (heavy white discharge, Nugent score of 8 and pH of 6.4 at final follow up). *G. vaginalis* was present at this visit in high counts (3.7×10^7^) whereas it was absent at the previous visits.

## Discussion

MABGEL 1 was the first study to assess the pharmacokinetics and safety of topical application of mAbs in the human female genital tract. Differences were detected in the rate of vaginal elimination and half-lives between 2G12 and the MPER mAbs 2F5 and 4E10. Whereas 2F5 and 4E10 declined exponentially, with t ½ around 4 to 5 hours, 2G12 levels declined more rapidly initially, with slower decline at lower concentrations. Interestingly, such findings differed from those seen in a parallel PK study in cynomolgus macaques (*Macaca fascicularis*) [52]. Macaques (n = 6) were vaginally administered MABGEL containing 20 mg/g of each mAb (i.e. the same as used by women in the high-dose MABGEL arm). In view of their smaller-sized vaginas, 2 g rather than 2.5 g gel was applied; Weck-cel sampling, processing methodology and detection ELISAs were the same as employed in our trial, although published data did not include adjustment for dilution factors. In macaques, all sampling was performed at serial time-points following a single application of the gel. In women, for reasons of comfort and logistics, samples at the 12 and 36 hour time points were performed after the 12^th^ dose. For 2F5 and 4E10, average (mean or median) vaginal concentrations in macaques, at equivalent time-points, were very similar to those seen in the high dose Mabgel arm before adjustment for dilution factors. In contrast, 2G12 levels were significantly higher in macaques, declining exponentially at a similar rate to the MPER mAbs. However, it should be noted that all animals in this study were pre-treated with Depo-Provera (DMPA) whereas women in the MABGEL arms of our trial were either naturally cycling (n = 10) or using a range of non-DMPA hormonal contraception (COCP n = 1; desogestrel-POP n = 1, intra-uterine system n = 2, implant n = 5). Further research designed specifically to study the effects of particular hormonal contraceptives on genital tract mAb pharmacokinetics would be of benefit.

As we have described, we conducted an *in vitro* mAb stability study to explore our in vivo findings further. We found that in vaginal secretions obtained from naturally-cycling women with normal vaginal flora, in the absence of protease inhibitors, 4E10 was the most stable, 2F5 levels declined slightly more quickly and 2G12 levels, as *in vivo*, declined rapidly initially with slower decline at lower concentrations.

The apparent reduced stability of 2G12 in human vaginal secretions compared to the MPER mAbs may be due to its structure. 2G12 has an unusual tertiary structure, with interlocked, domain-exchanged variable heavy chain (V_H_) regions giving it an extended linear configuration rather than a classical Y or T shape. This configuration creates additional antigen-binding sites enabling nanomolar binding affinity to oligomannose clusters within gp120 [53]. IgG is susceptible to proteolysis by a number of physiological enzymes including plasmin, pepsin, trypsin, cathepsins, elastases. The hinge region is the most susceptible part of an antibody to cleavage, and more sites around the hinge region become susceptible to enzymatic cleavage after exposure to low pH [54]. Crystallographic analysis of 2G12 has shown that there is an adaptable hinge between the V_H_ and C_H_1 (1^st^ constant heavy chain) regions which permits the domain exchanged structure [53], and we propose that this region is susceptible to accelerated physiological proteolysis in the acidic human cervico-vaginal environment, that is not seen in macaques or in plasma.

Comparisons of the vaginal microflora between humans and macaques, both cynomolgus and the larger pig-tailed macaque (*Macaca nemestrina*), have shown similarities in the range of bacterial species identified, through microscopy and culture, but differences in the proportion in which each is present. For example, Lactobacilli are generally found in lower density and Viridans streptococci in higher density in macaques than is usual for the healthy human vagina, with most hydrogen-peroxide production attributable to the latter rather than the former [55], [56]. In general, vaginal pH is higher in macaques, being 5.5 to 8 in *M. fascicularis*, and 4 to 8.5 in *M. nemestrina*, with 6 to 7 being most common [56], [57]. This compares with 3.8 to 4.5 in healthy women [58]. Interspecies differences in vaginal pH, and perhaps in other, as yet undetermined, variables e.g. in the enzymic activity of mucosal secretions, could underlie the observed differences in 2G12 genital tract pharmacokinetics in macaques compared with women, and may have implications for evaluating the function/persistence of other protective agents.

The pharmacokinetics of intravenously administered 2G12, 4E10 and 2F5 have been studied previously in HIV positive individuals [19], [20]. In contrast to its more rapid elimination in cervico-vaginal secretions, in plasma 2G12 displays a much longer elimination *t_1/2_* (14.1 days) compared to 4E10 (6.6 days) and 2F5 (3.2 days) [18]. These plasma *t_1/2_* differences have never been fully explained. Differences in recycling by the neonatal Fc receptor (FcRn) is one possible mechanism. As all 3 mAbs share identical IgG1k constant (Fc) regions, they would be expected to have similar FcRn binding affinities. However, recent observations suggest that the structure of the variable (Fv) domain may influence FcRn binding through steric influences on the Fc region [59]. Formal assessment of FcRn–mAb interactions may therefore be warranted.

Although FcRn has been shown to be expressed by human female genital tract epithelium [60], our data, showing no systemic uptake of the mAbs, and the much shorter half-life of the mAbs in the genital tract compared to plasma, do not support a functional role *in vivo* for FcRn. However, one limitation of our study is that we did not perform tissue biopsies to look for any local retention. Further studies using tissue biopsies, including in sexually active women, may be warranted.

Even outwith any potential influence on FcRn binding, differences in Fv structure and overall molecular charge between 2G12 and the MPER antibodies could still impact on their pharmacokinetics. In general, the more basic (higher) isoelectric point of most antibodies compared with other serum proteins (pI 6.5 to 10 vs <5.5) tends to favour their retention in tissues, as it renders them more likely to bind to negatively charged moieties on cell surfaces or within the extracellular matrix [61], andenhances cellular uptake by pinocytosis [62], [63]. Overall, there is a trend for antibodies to show greater uptake and binding by tissues, and a resulting increase in their plasma clearance, with increasing cationization. In contrast, although anonized antibodies generally show less tissue uptake, they can display either increased or decreased plasma and total body clearance [61], [63]. One might predict from their higher pIs (9 to 10 for 2F5 and 9.5 for 4E10 vs 7.5 to 8.5 for 2G12, B Vcelar, Personal Communication), and possibly their greater affinity for lipids [64], that the MPER mAbs could be taken up more readily by tissues than 2G12. This could explain their more rapid plasma clearance. In addition, it could lead to them being retained in greater concentrations in mucus and/or the superficial layers of the vaginal epithelium [65]. The latter warrants further exploration, e.g. through studying mAb distribution in cervico-vaginal tissue biopsies *ex vivo* following *in vivo* MABGEL application or in cultured explants.

Data from explant studies and the macaque SHIV challenge system has clarified that mAbs need to exhibit both potent HIV-1 neutralization and FcγR-mediated activity to block vaginal transmission [66]–[68], properties exhibited by all 3 of the mAbs in our trial [52]. In addition, breadth of coverage against circulating HIV strains and mechanism of neutralization e.g. degree of dependence on viral Env-host cell CD4 receptor interactions, whether reversible or irreversible, will also be important *in vivo*. Probably the key feature for real world implementation will be breadth of coverage, as the smaller the number of mAbs needed to neutralise as close as possible to 100% of transmitted strains, the lower would be production costs, and the greater the ease of manufacturing scale up and clinical development. 4E10 is a truly remarkable mAb in this regard, with neutralisation of 96, 98, & 100% of strains in various analyses [8], [9], [10]. Although series of mAbs with at least 10-fold [9], [69] and 100-fold [10] greater potency than 4E10 have now been described, very few have the same breadth as 4E10. One exception is the recently described 10E8, which also neutralised 98% of strains, and targets an epitope overlapping with that of 4E10, but is around 6 fold more potent [70]. Although demonstrating reduced breadth of coverage than 4E10 and 2F5, 2G12 neutralizes HIV much more rapidly than the MPER mAbs, which take several hours to mediate maximal neutralization *in vitro* [8], [71]. However, whereas the former can induce Env shedding, resulting in permanent viral inactivation, 2G12-mediated neutralization is reversible [71]. All 3 mAbs have been shown to act synergistically *in vitro* (B. Vcelar, Personal Communication).

The potential correlation of many *in vitro* assays analyzing mAb functionalities with *in vivo* protection against HIV infection is currently unclear. In a recent explant study, nicotiana-produced 4E10 was shown to be more effective at blocking HIV-1_JR-CSF_ infection of *ex vivo* polarized human cervical tissues than the newer gp 120 binding HIV neutralizing mAbs PG9 and PG16, despite the latter having *in vitro* IC_90_ values that were measured>200 and>2000 fold lower [72]. However, 10 times more 4E10 was required to block infection of tissue compared to the TZM-bl assay. 2F5 has also been shown to be efficacious at blocking HIV infection (HIV _BaL_) in a cervical explant system [68].

To date, the amount of antibody needed at mucosal surfaces to prevent HIV-1 infection has not been fully established. However, comparisons can be made between the mAb levels detected in vaginal secretions in our Phase 1 trial and those shown to block HIV transmission in SHIV non-human primate (NHP) vaginal transmission models. For SHIV NHP studies, data is available for vaginal concentrations of (a) transudative mAbs present after intravenous infusion, or (b) directly vaginally administered mAbs. In a recent study, 5 macaques received a single high dose vaginal challenge of SHIV_BaL_ following a 25 mg/kg IV infusion of 2F5. The challenge inoculum was administered 6 hours post infusion at the time of peak vaginal 2F5 concentrations, mean 306 µg/ml (range 19.4 – 463.2 µg/ml). No animals became infected [68]. These researchers used the same sampling techniques, ELISA methodology and dilution factor correction as ourselves, and by extrapolation the median levels of 2F5 we detected in both the high and low dose MABGEL arms at 8 hours (post 1^st^ dose) and high dose at 12 hours (post 12^th^ dose) should be sufficient to block HIV transmission. However, luminal mAb concentrations following iv mAb infusion are dependent on the systemic-mucosal gradient, and may not adequately reflect the concentration of mAbs within tissues [68]. In a study designed specifically to investigate the prophylactic efficacy of 2 mls of high dose (20 g/ml) MABGEL, progesterone-treated macaques in 4 groups (untreated controls (n = 6), placebo-treatment 1 hour before challenge (n = 9), placebo-treatment 4 hours before challenge (n = 9), MABGEL 1 hour before challenge (n = 9) and MABGEL 4 hours before challenge (n = 6)) were challenged intra-vaginally with a stock containing 4–10 animal infectious doses (AID_50_) of SHIV_162P3_ in 50% human seminal plasma. 5/6 untreated control animals were infected, as were 15/18 animals who received placebo. Efficient protection from acquisition of SHIV infection was obtained with MABGEL only and with equal efficacy at 1 h (6/9 uninfected, Chi-Square p = 0.038) and 4 h (4/6 uninfected, Chi-Square p = 0.037) post-challenge [52]. As for the macaque PK study discussed above, vaginal 2F5 and 4E10 concentrations detected at time of challenge were similar to corresponding values found in our study, but levels of 2G12 were higher. However, as both of these transmission studies utilized macaques with progesterone-induced vaginal thinning not seen in humans, and challenge inocula at copy number higher than in human ejaculate, our data clearly suggest the potential for a protective effect to occur in women.

We observed no toxicity associated with 12 days of MABGEL administration. Although vaginal bleeding was reported in almost half of the participants, this, like the other genital AEs, was seen equally as frequently in the active and placebo arms. Intermenstrual bleeding is commonly reported in vaginal microbicide trials [73]. Although a number of mildly elevated PT results were detected, none was temporally associated with a bleeding event, and in view of the lack of systemic absorption of the mAbs and the normal alanine transaminase, aPTT and aCL titres, these were felt to be related to intrinsic variability in the laboratory PT assay. The remainder of the clinical ARs were also evenly distributed between study arms and judged as reflecting background morbidities.

Given the association between perturbations of the vaginal microflora and increased risk of HIV acquisition [36]–[38], evaluation of the effect of topical products on vaginal microflora is essential [39]. Overall, using qPCR, there was no evidence to suggest that the presence or absence of any *Lactobacillus* species was affected by the application of study gel, and no statistically significant differences were seen across the groups. The lower percentage detection of *L. iners* in the low dose arm was present from screening (p = 0.04 (Fisher's Exact)), thus was independent of study gel use. Performance of our end of dosing evaluations at 36 hours post last dose could have potentially missed some changes due to the dynamic nature of the vaginal flora. Due to the expected low frequency of *G. vaginalis* and *A. vaginae*, no significant conclusions can be reached with regards to these and therefore further evaluation of the effect of the Mabgel on these and other BV-associated species is needed in settings with high BV prevalence e.g. in a phase II trial [39]. Although our study was not designed to directly compare methodologies for assessing the vaginal flora, it is reassuring to note that in general there was agreement between clinical, microscopic and qPCR assessments and this increases the validity of our findings.

Further consideration needs to be given to the development pathway for mAbs as microbicides. The discovery of novel, increasingly potent mAbs, particularly 10E8 [70], and alternative plant-based production methods [74], [75], increases their potential to be effective and affordable HIV prevention agents. Moreover our data suggests that even at the level of 4E10 potency, single dosage can achieve sufficient *in vivo* concentrations for transmission blockade for many hours. Thus extreme breadth and depth of activity could be achieved for example with a triple combination of 4E10, PGV04 & VRC01 [10], [69] and this could be considered as a prototype product. Recent data have even suggested the possibility of both systemic and genital tract vectored immuno-prophylaxis, which would be able to provide long term mAb delivery and protection [76]–[78].

In conclusion, in our study, daily application of up to 50 mg each of 2F5, 4E10 and 2G12 to the vagina was shown to be safe over a 12 day period, and achieved monoclonal antibody concentrations potentially capable of blocking HIV transmission. However, our findings highlight the importance of clinical assessment of stability, and ideally function, in the vaginal environment of future mAb microbicide candidates. Further research, including the evaluation of genital tract mAb tissue distribution following vaginal application, functional studies to assess the neutralization and FcR –binding capacity of recovered antibody, broader evaluation of the effects on the vaginal bacterial and immune milieu and comparison between hormonal contraceptive users and naturally cycling women is required and warranted to develop more potent mAb combinations as microbicides.

## Supporting Information

S1 CONSORT Checklist Checklist for MABGEL1 Clinical Trial.
(DOC)

S1 Protocol Final Approved Protocol for MABGEL1 Clinical Trial.
(PDF)

S2 Protocol Final Approved Protocol for *In vitro* stability study of 2F5, 4E10, 2G12 in vaginal secretions.
(PDF)
