# Supplementary material for: MABGEL 1: First Phase 1 Trial of the Anti-HIV-1 Monoclonal Antibodies 2F5, 4E10 and 2G12 as a Vaginal Microbicide
Source: PLoS One. 2014 Dec 29;9(12):e116153. doi: 10.1371/journal.pone.0116153 (PMC4278856; doi:10.1371/journal.pone.0116153)
Supplement: S1 Protocol — Final Approved Protocol for MABGEL1 Clinical Trial. (PDF) [file pone.0116153.s002.pdf]

Acronym: MABGEL 1

A randomised double blind phase 1 study to assess the pharmacokinetics of C2F5, C2G12 & C4E10 when administered together in a gel vehicle as a vaginal microbicide

Protocol code: Mabgel1
EudraCT number: 2008-000312-32
ISRCTN: ISRCTN64808733

Version 3.3

Protocol date 29 March 2010

(Based on MRC CTU template protocol version 3.15)

Authorised by:

Name: Prof Charles Lacey Role: Chief Investigator

Signature: Date:

GENERAL INFORMATION

Sponsor
York Hospitals NHS Foundation Trust and the University of York will act as the co-Sponsors for the study.

Funder
The study is funded by the European Commission through the European Microbicides Programme (EMPRO).

Authorisation
The study will be authorised by Professor Charles Lacey.

Monitoring
The study will be monitored by Covance.

Main Contacts:

Medical expert
Professor Charles Lacey

Chief Investigator
Professor Charles Lacey
Department of GU Medicine
31 Monkgate
York, YO31 7WA, UK
charles.lacey@hyms.ac.uk
Tel: 44 1904 725474
Fax: 44 1904 642116

Investigators
Dr Georgina Morris (clinical research fellow) georgina.morris@hyms.ac.uk
Dr Rebecca Wiggins (laboratory procedures)
Clinical Staff from the Experimental Medicine Unit, York Hospitals NHS Foundation Trust
Clinical Staff from the York HIV Research Group

Co- Investigators
Dr Brigitta Vcelar
Polymun Scientific,
Nussdorfer Laende 11,
A-1190 Vienna, Austria
brigitta.vcelar@boku.ac.at
Tel : 43 1 36006 6209
Fax: 43 1 3697615

Dr Vicky Jespers
Institute of Tropical Medicine,
Antwerp, Belgium
vjespers@itg.be

Trial management
Dr Georgina Morris georgina.morris@hyms.ac.uk

Trial statistician
Dr Victoria Allgar
Hull York Medical School
University of York
York YO10 5DD, UK
victoria.allgar@hyms.ac.uk

Data Management

Covance Clinical Research Unit
Springfield House
Hyde Street
Leeds
LS2 9LH

Clinical laboratories, medical and technical departments, institutions
All participant visits will take place at York Hospital. Safety assays and haematological, biochemical microbiological screening tests will be conducted at the Pathology Laboratories, York Hospital. Samples for pharmacokinetic assays and vaginal flora will be processed and stored at the HYMS Experimental Medicine Unit (EMU) at York Hospital, or at the Immunology and Infections Unit at the University of York. The pharmacokinetic assays will be performed at Polymun Scientific, Vienna. Vaginal flora assays will be performed at the Institute of Tropical Medicine, Antwerp and the Immunology and Infection Unit at the University of York.

Definitions

Chief Investigator: To take ultimate responsibility for the design, conduct, analysis and reporting of a clinical study/trial.

Investigator: An individual member of the clinical trial team with designated responsibilities, who in terms of the clinical trial is under the overall supervision of the Chief Investigator

PROTOCOL SIGNATURE PAGE

The signatures below confirm agreement by the individuals authorised by the Sponsor and principal participating institution at the clinical site responsible for signing the clinical trial agreement, that study MABGEL1 (protocol version 3.3) will be conducted in accordance with this protocol and UK Clinical Trials Regulations. Any amendments to this protocol that have a direct influence on the participants in the trial will be approved by the relevant ethics committees before implementation.

____________________	__________________	____________________	____________
Chief Investigator signature	Printed name	Designation	Date
____________________	__________________	____________________	____________
Sponsor signature	Printed name	Designation	Date

I, the Chief Investigator, agree to conduct this study in full accordance with the provisions of this protocol and will comply with all requirements regarding the obligations of clinical investigators in accordance with the UK Clinical Trials Regulations. I agree to allow Sponsor monitors and auditors full access to all medical records at the research facility for participants screened or enrolled in the study.

I agree to maintain all study documentation until the Sponsor consents to disposal of files in writing. I also agree to publish or present data only upon review and approval by the EMPRO consortium.

I have read and understand the information in the Investigator's Brochures including the potential risks and side effects of the product under investigation, and will ensure that all associates, colleagues, and employees assisting in the conduct of the study are informed about the obligations incurred by their contribution to the study.

SAE NOTIFICATION

In the case of a SAE, complete a SAE report form and forward by fax to the North and East Yorkshire R&D Alliance within 24 hours of becoming aware of the SAE.

Fax number:
North and East Yorks R& D Alliance:
01904 731297

CONTENTS

1. Study summary 10
2. Study flow chart 11
3. Introduction 12
3.1 Background 12
3.2 In vitro data 12
3.3 Studies in animal models 13
3.4 Clinical research in human subjects 17
3.5 Rationale for study objectives 18
4. Study objectives 19
5. Study design 20
5.1 Study Centre 20
5.2 Type of design 20
5.3 Disease/participants studied 20
5.4 Trial interventions – research and control 20
5.5 Outcome measures 20
5.6 Duration 21
5.7 Sub-studies 21
5.8 Organisational summary 21
6. Recruitment of participants 22
6.1 Inclusion criteria 22
6.2 Exclusion criteria 22
6.3 Number and source of participants 23
6.4 Screening procedures and pre-randomisation investigations 23
7. Assessments and follow-up 26
7.1 Schedule for follow-up 26
7.2 Visit 2: Randomisation and trial entry, pre- and post-1st dose 28
7.3 Visit 3, 8 hours post 1st dose 29
7.4 Visit 4, 24 hours post 1st dose 29
7.5 Dosing at home 29
7.6 Telephone calls 29
7.7 Visit 5, day of the 5th to the 7th dose 29
7.8 Visits 6 and 7, timed post 12th dose 29
7.9 Visit 8: Final visit 29
7.10 Unblinding individual participants 30
7.11 Co-enrolment 30
8. Details of study products 31
8.1 Products 31
8.2 Packaging and labelling 31
8.3 Storage of the gels 32
8.4 Treatment schedule and dispensing 33
8.5 Dose modifications 33
8.6 Accountability and unused drugs/devices 33
8.7 Measures of compliance and adherence 33
8.8 Non-trial treatment 33
9. Safety reporting 35
9.1 Definitions 35
9.2 Causality 36
9.3 Reporting procedures 37

9.4 Withdrawal of participants from the study 38
9.5 Temporarily halting or stopping the study 38

10. Statistics and analysis 40
10.1 Sample size 40
10.2 Planned statistical analyses 40
10.3 Randomisation scheme and maintenance of blinding 41
10.4 Analysis of biological samples 42

11. Management of data, samples and trial procedures 44
11.1 Data management 44
11.2 Management of biological samples 45
11.3 End of the study 45
11.4 Risk assessment 45
11.5 Trial monitoring 45
11.6 Trial management 46

12. Sub-studies 47
12.1 Investigation of the impact of Mabgel on vaginal flora 47
12.2 Comparison of self-sampled, to clinician-sampled vaginal secretions 47
12.3 Investigation of the experience of taking part in the clinical trial 48

13. Ethical Considerations and Approval 49
13.1 Ethical approval 49
13.2 Informed consent 49
13.3 Confidentiality 49
13.4 Risks 50
13.5 Benefits 51
13.6 Reimbursement 51
13.7 HIV testing 51

14. Regulatory issues 52
14.1 Required approvals 52
14.2 GCP and GMP compliance 52

15. Indemnity 53
15.1 Negligent harm 53
15.2 Non-negligent harm 53

16. Finance 54
16.1 Funding 54
16.2 Reimbursement for participants 54

17. Publication 55

18. Protocol Amendments 56

19. References 57
Inclusion criteria 84
Exclusion criteria 84

LIST OF TABLES

Table 3-1: Results of intravaginal challenge study in adult macaques. Number of protected and infected animals after passive infusion of MAbs and challenge with SHIV-89.6PD....14
Table 3-2: Passive immunization against oral SHIV challenge in neonatal macaques....15
Table 3-3: Overview of clinical trials using C2F5, C2G12, and C4E10 to date....17
Table 7-1 : Schedule of visits....27
Table 8-1: Study gels....31

LIST OF FIGURES

Figure 3-1 : Antibody concentrations in lavages obtained 1, 4 and 12 h after vaginal application of Mabgel to macaques....16
Figure 3-2: Antibody concentrations in wick samples obtained up to 24h after vaginal application of Mabgel to macaques....16

LIST OF APPENDICES

Appendix 1: Brief participant information sheet.... 61
Appendix 2: Participant information sheet.... 63
Appendix 3: Consent form.... 81
Appendix 4: GP Letter.... 82
Appendix 5: Mabgel – storage instructions and instructions for use.... 87
Appendix 6: Grading of genital reactions.... 89
Appendix 7: Grading of clinical and laboratory adverse events.... 90
Appendix 8: Source data definition.... 95

ABBREVIATIONS

**aCL**	Anti-cardiolipin
**ADR**	Adverse drug reaction
**AE**	Adverse event
**APTT**	Activated partial thromboplastin time
**AR**	Adverse reaction
**CI**	Chief Investigator
**CRF**	Case report form
**CT**	Chlamydia Trachomatis
**CTA**	Clinical Trials Authorisation
**DMC**	Data Monitoring Committee
**EMPRO**	European Microbicides Programme
**EUDRACT**	European Union Drug Regulatory Agency Clinical Trial
**HIV**	Human Immunodeficiency Virus
**GC**	Gonorrhoea
**GU**	Genitourinary
**HYMS EMU**	Hull York Medical School Experimental Medicine Unit
**IB**	Investigator's brochure
**ID**	Identification number
**IDMC**	Independent Data Monitoring Committee
**IIU**	Immunology and Infection Unit
**IMP**	Investigational medicinal product
**ISRCTN**	International standard randomised controlled trial number
**MAbs**	Monoclonal antibodies
**MHRA**	Medicines and Healthcare Regulatory Authority
**MRC CTU**	Medical Research Council Clinical Trials Unit
**NHS**	National Health Service
**PEP**	Post exposure prophylaxis
**PI**	Principal Investigator
**PT**	Prothrombin time
**QOL**	Quality of life
**REC**	Research Ethics Committee
**SAE**	Serious adverse event
**SAR**	Serious adverse reaction
**SID**	Screening Identification Number
**SOP**	Standard operating procedures
**SPC**	Summary of product characteristics
**SSA**	Site specific assessment
**STI**	Sexually Transmitted Infection
**SUSAR**	Suspected unexpected serious adverse reaction
**TMF**	Trial Master File
**TMG**	Trial Management Group
**TSC**	Trial Steering Committee
**UAR**	Unexpected adverse reaction

1. STUDY SUMMARY

**TITLE**	MABGEL 1: A randomised double blind phase 1 study to assess the pharmacokinetics of C2F5, C2G12 & C4E10 when administered together in a gel vehicle as a vaginal microbicide
**DESIGN**	A single centre, three-arm, double blinded phase I randomised clinical trial.
**OBJECTIVES**	The primary objective is to assess the pharmacokinetics of the specified monoclonal antibody combination when applied vaginally.
**POPULATION**	Healthy, HIV-negative, women aged ≥18 and ≤45 years.
**INTERVENTIONS**	Vaginal microbicide gel: (a) high-dose monoclonal antibodies (50mg each of C2F5, C2G12 and C4E10) (b) low dose monoclonal antibodies (25mg each of C2F5, C2G12 and C4E10) or (c) a control placebo vaginal microbicide gel.
**OUTCOME MEASURES**	Primary endpoint:<br>• levels of MAbs in Weck-Cel vaginal secretions 1, 8 & 24 hours post-1st dose and 12 & 36 hours post-12th dose<br><br>Secondary endpoints:<br>• 24 hour post-1st dose and 36 hours post-12th dose cervico-vaginal lavage MAb levels<br>• 24 hour post-1st dose and 36 hours post-12th dose Mab levels in self-sampled vaginal aspirate<br>• 8 hours post-1st dose and 12 hour post-12th dose serum MAb levels<br>• Number of grade 3 or above genital adverse events during the dosing or follow up period<br>• Number of grade 3 or above other clinical or laboratory adverse events confirmed at examination or on repeat testing respectively during the dosing or follow up period<br>• Number of events attributable to the study gel leading to discontinuation of gel
**DURATION**	The study takes place over a minimum of 3 menstrual cycles. Screening will take place 2 to 6 weeks before the first dosing visit. Dosing will take place on a daily basis over 12 days, starting on day 7-13 of the menstrual cycle. Further genital examinations with systemic and genital tract samples will be taken 12 & 36 hours after the last dose. The final follow-up visit will be conducted in the subsequent menstrual cycle.
**CO-SPONSORS**	York Hospitals NHS Foundation Trust and the University of York
**FUNDER**	European Commission via the European Microbicides Programme
**CHIEF INVESTIGATOR**	Prof Charles JN Lacey

2. STUDY FLOW CHART

Locate participant suitable for entry: briefly check inclusion/exclusion criteria, check menstrual cycle. Provide participant information sheet. Arrange appointment for screening visit.

↓

Screening visit: check eligibility criteria obtain informed consent, physical and cervico-vaginal examinations, colposcopy, GU infection and blood test screening.

↓

Check results of screening tests, if eligible for entry then invite for visit 2 and entry into dosing stage of trial. If ineligible refer onward/treat/invite for re-screening at a later date as appropriate.

↓

Visit 2: Cervico-vaginal examination, colposcopy. Randomise to one of the three trial arms. Blood and genital tract samples. Administer 1st dose.

↓

Visit 2, 1 hour after dosing: Cervico-vaginal examination, colposcopy, genital tract samples.

↓

Visit 3, 8 hours after dosing: Cervico-vaginal examination, colposcopy, blood and genital tract samples.

↓

Visit 4, 24 hours after 1st dose: Cervico-vaginal examination, colposcopy, genital tract samples.

↓

Phone calls, day of 2nd-4th and 8th-10th doses: Check compliance and tolerability

↓

Visit 5, day of the 5th to 7th dose: Cervico-vaginal examination, colposcopy,

↓

Visit 6, 12 hours post 12th dose: Cervico-vaginal examination, colposcopy, blood and genital tract samples.

↓

Visit 7, 36 hours post 12th dose: Cervico-vaginal examination, colposcopy, genital tract samples.

↓

Visit 8, subsequent menstrual cycle: Physical examination, cervico-vaginal examination, blood and genital tract samples, qualitative interview.

3. INTRODUCTION

3.1 Background

In an attempt to control the HIV epidemic the development of prophylactic intravaginal microbicides is now a high research priority[1,2]. Currently the only commercially available 'female-controlled' method of HIV prevention is the female condom. However, although the woman initiates use of the female condom, it cannot be used without the knowledge of her male partner, requires his co-operation, has a number of features in use that diminish its acceptability, and is relatively expensive. Therefore in further evaluations the female condom has been associated with high discontinuation rates, and is of uncertain effectiveness[3]. Consequently several vaginal microbicides, such as PRO 2000 are now in phase III clinical trials to determine their effectiveness[4-6]. However, these trials are all investigating the use of polyanionic compounds as vaginal microbicides, and it is possible, or perhaps likely, that specific antiretroviral agents or combinations of such antiretroviral agents will be needed to be developed as microbicides to achieve high levels of protection against vaginal HIV transmission[7].

The antibodies C2F5, C2G12 and C4E10 are human recombinant monoclonal antibodies (MAbs) which potently, broadly and synergistically neutralize divergent HIV-1 subtypes[8]. They have been administered intravenously to human subjects in high doses with demonstrated safety[9,30]. When administered intravenously they have also protected adult female macaques against vaginal challenge with SHIV 89.6PD[10]. Therefore this combination of monoclonal antibodies could potentially represent an extremely promising vaginal microbicide for use in women.

3.2 In vitro data

The primary pharmacological action of the monoclonal antibodies C2F5, C2G12 and C4E10 is binding to conserved epitopes on the envelope of HIV-1 and subsequent virus neutralization. The monoclones are recombinant human IgG1 antibodies of average molecular weight 150 kD.

C2F5

The antibody C2F5 recognises a highly conserved epitope on the ectodomain of the HIV-1 gp41, identified to consist of the amino acid sequence ELDKWA[11]. The presence of the LDKW sequence was found to be crucial for recognition of peptides and these findings correlated with the virus neutralisation sensitivity. Subsequently it was also shown that C2F5 broadly neutralises primary HIV-1- isolates from different HIV-1 subtypes.

C2G12

C2G12 recognises a conformational dependent epitope on gp120. Binding of C2G12 to monomeric gp120 is abolished by amino acid substitutions removing N-linked glycosylation in the C2, C3, V4 and C4 regions of gp120[12-13]. Using crystal structures of FAb 2G12 it was demonstrated that this antibody binds to an unusually dense cluster of carbohydrate moieties on the "silent" face of gp120 by a previously unappreciated mechanism for high affinity recognition of carbohydrate epitopes[14].

C4E10

C4E10 binds to a highly conserved epitope on the ectodomain of the HIV-1 envelope gp41 consisting of the amino acid sequence NWFDIT. It is located subsequent to the C2F5 epitope closer to the C terminus of gp41[15-16].

C2G12 has been demonstrated to inhibit binding of HIV-1 to the CCR5 coreceptor [12;17-18]. C2F5 and C4E10 bind to epitopes exposed after virus attachment, and binding to these pre-hairpin intermediates interferes with the virus-cellular membrane fusion process preventing infection of target cells[19].

In vitro, neutralising antibodies prevent infection of target cells by blocking of envelope binding to receptors, blocking infection at the stage of fusion or by inducing conformational changes in gp120[19], although additional mechanisms may operate *in vivo*[20]. Neutralization by C2F5, C2G12 and C4E10 has been intensively studied with a large number of primary HIV-1 isolates of divergent clades [12;15;16;21-27].

3.3 Studies in animal models

The preventive potential of C2F5, C2G12 and C4E10, the active ingredients of Mabgel, was investigated in the SHIV/macaque model. The SHIV/macaque model is considered to be the most relevant model for investigating the pathogenesis of HIV-1 infection in humans. The construction of chimeric simian/human immunodeficiency virus (SHIV) isolates that express *env* genes derived from HIV-1 has made it possible to study human neutralising antibodies against HIV-1 in the macaque model. Different antibody combination and routes of applications have been investigated. The most relevant in terms of this clinical trial investigated mucosal SHIV challenge in macaques and are described bellow.

3.3.1.1 Intravaginal Challenge of Adult Macaques

Experiments were conducted at the Walter Reed Army Institute of Research (WRAIR) Rockville under the guidance of Drs. John Mascola and Mark Lewis, WRAIR, Rockville, MD/USA[10,32]. In this early study C2F5 and C2G12 were tested alone or in combination with polyclonal HIV specific antibody preparation (HIVIG). Control monkeys received irrelevant polyclonal non HIV-specific human intravenous immunoglobulin (IVIG). C4E10 was not available in sufficient quantities at the time of conduct. Challenge was performed using a highly pathogenic virus, SHIV 89.6PD, which depletes all $CD4^+$ target cells within two weeks after infection. Two levels of protection were observed after passive immunotherapy; complete protection from infection and low level infection associated with protection from disease progression.

Study Design

The antibodies were infused intravenously either individually, in double combination of C2F5 and C2G12, or in triple combination with HIVIG. SHIV-challenge (SHIV-89.6PD) was done intravaginally 24 hours post infusion of the MAbs in order to simulate the predominant way of HIV transmission. Antibody concentrations for infusion were defined based on neutralisation studies with primary isolates[33]:

Doses of antibodies:	IVIG	400 mg/kg
	HIVIG	400 mg/kg
	C2F5	15 mg/kg
	C2G12	15 mg/kg

Results

Whereas all five IVIG treated control monkeys displayed high plasma viraemia and rapid $CD4^+$ cell decline, the 14 antibody treated macaques were either completely protected against infection or against pathogenic manifestations of SHIV-infection (Table 3-1). Complete protection was achieved in four of five monkeys in the HIVIG/C2F5/C2G12 group, two of five in the C2F5/C2G12 group, and two of four in the C2G12 group. In addition, antibody-treated monkeys that became infected displayed low or undetectable plasma RNA concentrations and only modest decline in $CD4^+$ cell counts. This protection against SHIV-associated disease was more pronounced than in the intravenous challenge study[10,32].

Table 3-1: Results of intravaginal challenge study in adult macaques. Number of protected and infected animals after passive infusion of MAbs and challenge with SHIV-89.6PD

Infused MAbs	Total number	No infection[1]	Infection, no disease progression[2]	AIDS[3]
Irrelevant IVIG	5	none	None	5
HIVIG/C2F5/C2G12	5	4	1	none
C2F5/C2G12	5	2	3	none
C2G12	4	2	2	none

[1] No viral infection detectable by RT-PCR and co-culture
[2] Viral infection detectable but low viral loads and no progression to AIDS
[3] High viral loads, profound $CD4^+$ loss, progression to AIDS

3.3.1.2 Macaques/Mother-Infant Transmission Model

These experiments aimed to develop an immune prophylaxis against mother-to-infant HIV transmission. For this purpose a SHIV infection model in neonatal macaques was established mimicking intrapartum and postnatal mucosal virus exposure (Baba TW et al 1994). Experiments of this type were performed under the guidance of Dr. Ruth Ruprecht from Daner Faber Cancer Research Institute, Harvard University, Boston, Massachusetts; USA. In the mother-to-infant studies C2F5 and C2G12 were tested in combination with other monoclonal antibodies (IgG1b12 or F105, respectively). C4E10 was added in later studies as soon as sufficient quantities of clinical grade material were available. MAb combinations including C4E10 provided better protection upon challenge of neonate macaques (Ferrantelli et al. 2003). Combinations of MAbs were capable of providing protection from infection or from disease progression after oral challenge with different virus strains in neonate macaques. Passive immunisation was effective as pre-exposure prophylaxis, but also as post-exposure prophylaxis when administered up to 12 hours after virus exposure (Ferrantelli et al. 2003). Passive immunisation was studied in adult and neonatal macaques, including protection against different routes (i.v. or oral), different strains of SHIV (SHIV-vpu^+, SHIV89.6P, or C clade SHIV-1157ip), as well as different dose-schedules of human MAbs in triple or quadruple combinations. The goal of these animal studies was to develop a safe and effective approach to prevent mother-to-infant transmission of HIV with passive immunisation. Experiments focused on prevention of intrapartum and postnatal virus transmission through breast milk, two of the three recognised modes of maternal HIV transmission.

Taken together, challenge experiments in new-born monkeys resulted in 22 out of 31 neonates completely protected against infection. Table 3-2 summarises the results of passive immunisations using different combinations of the antibodies C4E10, C2F5, and C2G12 in the neonate macaque/SHIV-challenge model.

Passive immunoprophylaxis against SHIV challenge with combinations of C4E10, C2F5, and C2G12 was clearly shown to be :

- safe in neonate and adult rhesus monkeys
- efficient in neonatal rhesus monkeys against oral clade B SHIV challenge after i.v. antibody administration
- efficient in neonatal rhesus monkeys against oral clade C SHIV challenge after i.v. antibody administration
- efficient in neonatal rhesus monkeys against oral clade B SHIV challenge after i.m. antibody administration
- efficient in neonatal rhesus monkeys as prophylaxis and as post-exposure prophylaxis against oral SHIV challenge.

Table 3-2: Passive immunization against oral SHIV challenge in neonatal macaques

MAbs combinations	Dose (route) and time of	SHIV challenge (AID_{50})	Monkeys protected/ treated	Reference
2G12+2F5+F105	10 mg/kg (i.v.) 5d before birth, d0, d8	SHIV-vpu^{+} (10x)	4/4	Baba et al 2000
2G12+2F5+F105	10 mg/kg (i.v.) d0, d8	SHIV-vpu^{+} (10x)	2/2	Hofmann et al 2001; 2002a
2G12+2F5+F105	10 mg/kg (i.v.) PEP[i]: +1h, d8	SHIV-vpu^{+} (10x)	2/2	Hofmann et al 2002a
2G12+2F5+b12	10 mg/kg (i.v.) d0, d8	SHIV89.6P (15x)	1/4	Hofmann et al 2001
2G12+2F5+4E10 + b12	30 mg/kg (i.v.) PEP[i]: +1h, d8	SHIV89.6P (15x)	2/4	Ferrantelli et al 2003
2G12+2F5+4E10 + b12	30 mg/kg (i.v.) PEP[i]: +1h, d8 +12h, d8	SHIV89.6P (15x)	3/4 1/3	Ferrantelli et al 2007
2G12+2F5+4E10 + b12	30 mg/kg (i.v.) PEP[i]: +1h, d8	SHIV-1157ip (15x)	3/4	Xu et al unpublished
2G12+2F5+4E10	40 mg/kg (i.m.) PEP[i]: +1h, d8	SHIV89.6P (15x)	4/4	Ferrantelli et al 2004
**Total protected neonatal macaques**			**22/31**	

[i]PEP: post exposure prophylaxis

3.3.2 Pharmacokinetics of Mabgel after single vaginal application to macaques

Two studies were performed in the CEA animal facilities (Fontenay-aux-Roses, France). Six adult, female *Macaca fascicularis* received vaginally 2 g of antibody gel containing 20 mg C2F5, 20 mg C2G12 and 20 mg C4E10 per g.

In the first study animals were dispatched in three groups of two animals (one per time-point). Weck-Cel sampling followed by vaginal lavage was performed after 1 hour (group 1), 4 hours (group 2), 12 hours (group 3) to quantify C2F5, C2G12 and C4E10 antibodies. Blood was drawn 1, 4, 12 and 72 hours after application for quantification of antibodies in serum. Morbidity/mortality, local tolerance, rectal temperature and body weight were recorded. Vaginal application of 120 mg antibody in total was tolerated without adverse events by all animals. Weck-Cel sampling and lavages resulted in similar estimates for antibody concentration at the vaginal mucosa (Figure 3-1). Low quantities (nanogram range) of C2F5 and C4E10 were detected in the serum, whereas C2G12 was not detectable.

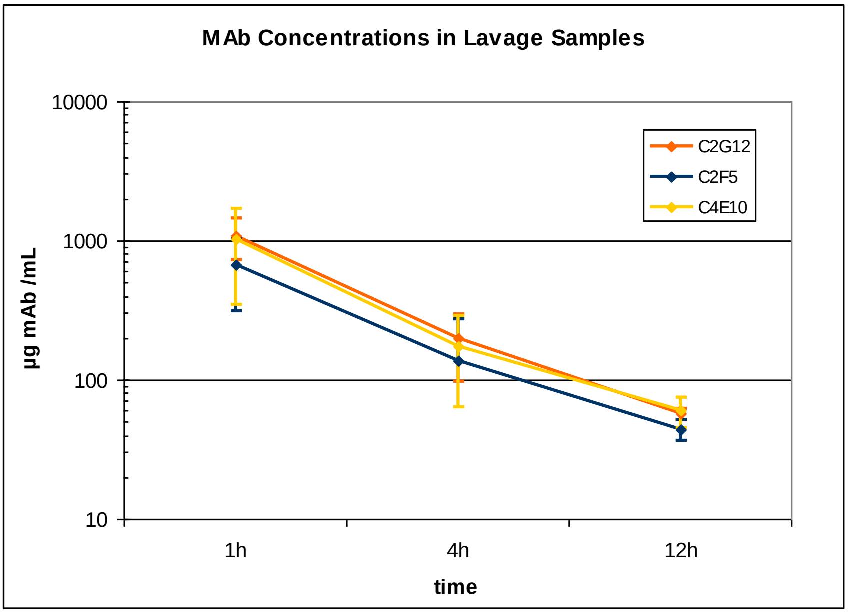

Figure 3-1 : Antibody concentrations in lavages obtained 1, 4 and 12 h after vaginal application of Mabgel to macaques

In the second study the same 6 adult, female *Macaca fascicularis*, after a washout period of 4 days, again received vaginal administration of 2 g of antibody gel containing 20 mg C2F5, 20 mg C2G12 and 20 mg C4E10 per g gel. Weck-Cel sampling was performed in all animals at 1, 4, 8, 12, 24, 48 and 72 hours to quantify C2F5, C2G12 and C4E10 antibodies. Morbidity/mortality, local tolerance, rectal temperature and body weight were recorded.

The second vaginal application of 120 mg antibody in total was again tolerated without adverse events by all animals. All three antibodies were detectable until 72 h with significant quantities (microgram range) detected until 24 h after application (Figure 3-2).

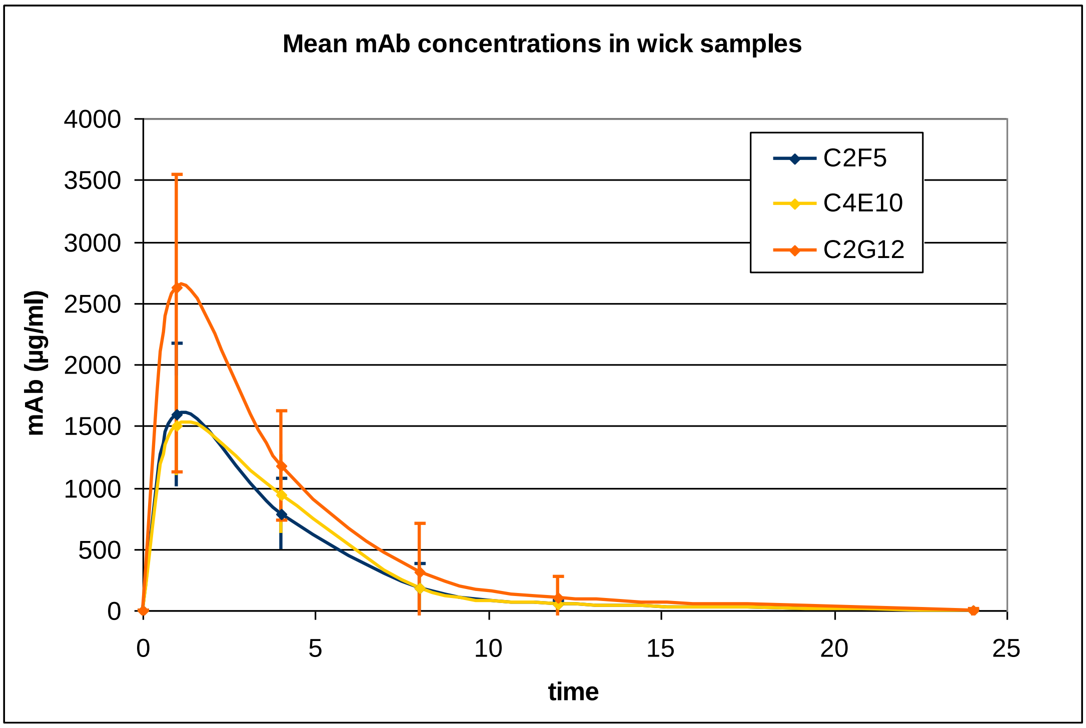

Figure 3-2: Antibody concentrations in wick samples obtained up to 24h after vaginal application of Mabgel to macaques

3.4 Clinical research in human subjects

To date, C2F5, C2G12, and C4E10 have undergone testing in four clinical trials[9;28-30]. These have evaluated the safety and efficacy of passive immunotherapy in a total of 39 HIV-1 infected subjects, receiving a total of 418 intravenous antibody infusions. Subjects received between 4 and 16 infusions of each monoclonal antibody at weekly intervals in doses ranging from 1 to 5G per infusion.

Table 3-3: Overview of clinical trials using C2F5, C2G12, and C4E10 to date

	Study			
	**1 Armbruster 2002[28]**	**2 Armbruster 2004[29]**	**3 Trkola 2005[9]**	**4 Mehandru 2006[30]**
No. patients	7	8	14	10
Male/Female	4/3	4/4	10/4	10/0
MAbs	C2F5, C2G12	C2F5, C2G12, C4E10	C2F5, C2G12, C4E10	C2F5, C2G12, C4E10
Dose per infusion (G)	1/1	1/0.5/1	1.3/1/1	1/1/1 (6 pts.)<br>2/1/2 (4 pts.)
No. infusion days	8	4	13	16
Length of dosing	4 weeks	4 weeks	13 weeks	16 weeks
SAEs	0	0	0	0
Thrombotic complications	0	0	0	0
Possible ADRs				
None	7/7	8/8	7/14	7/10
Myalgia	0	0	5/14	3/10
Arthralgia	0	0	4/14	1/10
Skin rash	0	0	1/14	1/10

The results of these trials are detailed in the Investigator's Brochure. In summary, no serious adverse events (SAEs) or thrombotic complications were reported, and very few possible adverse drug reactions (ADRs) were observed. The most common adverse events reported were myalgia, arthralgia, and skin rash. These were generally mild and resolved during or shortly after cessation of dosing. Direct evidence was obtained that these monoclonal antibodies contributed to the temporary containment of HIV-1 replication in infected humans.

Further analyses and experiments in relation to these clinical trials were recently reported, which investigated the possibility of autoreactivity and possible thromboembolic disorders associated with C4E10 and C2F5[31]. During trial 4 (as above table) the study design was modified to collect prospective data from the last four high-dose patients. Very mild (<1.25 x ULN) prolongation of the APTT 30 minutes post-infusion was observed which rapidly remitted, and there was no effect on the PT. In vitro experiments indicated that the effect on coagulation profile was mediated by 4E10. Further analyses indicated that C4E10, but not C2F5, also showed low-level cross-reactivity with cardiolipin, and that infusion of C4E10 resulted in transient low anti-cardiolipin antibody titres. It was concluded that there was little evidence that the monoclonal antibodies conferred an increased risk of thrombosis, but that monitoring of both coagulation parameters and anti-phospholipid antibodies should be carried out in any further clinical trials.

3.5 Rationale for study objectives

This trial is being conducted to assess the pharmacokinetics and safety of the monoclonal antibody combination when applied vaginally. As the antibodies have already been given systemically to HIV infected men and women without any serious adverse events, and the gel formulations using the same components are sold as over-the-counter products (Replens® vaginal lubricant), we have every expectation that the IMP will be safe when applied vaginally.

The primary objective of the study is therefore to understand the pharmacokinetics of the antibody combination when applied vaginally, as this will guide us in terms of future development of the monoclonal antibody combination. Specifically we need to know how long the antibodies persist in the lower female genital tract, as this will inform recommended dosing frequency for development of the microbicide. The a priori hypothesis is that the monoclonal antibodies will not be systemically absorbed because of their relatively large molecular weight. No systemic absorption was detected after repeated application to rabbits. However, recent data in macaques shows very low (nanogram range) systemic absorption of the antibodies when applied vaginally, so this provides the rationale to study this directly in healthy females. We will also monitor systemic and local safety parameters, including haematology, biochemistry and genital adverse events. The formal list of outcome measures is presented in section 5.5.

In summary, administration of C2F5, C2G12, and C4E10 to male and female HIV-infected patients has been judged to be safe and well-tolerated by the investigators in all human clinical trials conducted to date and there are no overt safety concerns with regard to vaginal administration of the MAbs to healthy, sexually abstinent female volunteers. The primary objective of this study is to assess the persistence of the MAbs in the vagina and the systemic absorption at two different doses.

4. STUDY OBJECTIVES

The primary objective of the study is to assess the pharmacokinetics of the specified monoclonal antibody combination when applied vaginally. Specifically:

- to assess the retention of the MAbs in the vagina after administration of the IMP
- to investigate whether there is any systemic absorption of the MAbs

The secondary objective of the study is:

- to assess the safety of the specified monoclonal antibody combination when applied vaginally

Further exploratory objectives of the study are:

- to investigate the changes in vaginal flora between the placebo and IMP groups after 12 daily doses of the study gel
- to compare self-sampled vaginal secretions to clinician-sampled vaginal secretions
- to investigate participants' experience of taking part in the clinical trial

5. STUDY DESIGN

5.1 Study Centre

The study will be carried out at York Hospitals NHS Foundation Trust. All visits will take place at York Hospital in conjunction with staff from the HYMS EMU.

5.2 Type of design

The study is a double-blinded, randomised, three-arm clinical trial to assess the pharmacokinetics of C2F5, C2G12, and C4E10 monoclonal antibodies (MAbs) when administered together in a gel vehicle as a vaginal microbicide.

5.3 Disease/participants studied

Healthy, HIV-negative, women aged ≥18 and ≤45 years who will remain sexually abstinent during the study dosing period, but are both willing and able to adhere to the trial conditions, methodology and to give written informed consent. For more details refer to section 6.

5.4 Trial interventions – research and control

Participants will receive vaginal microbicide gels containing either (a) high-dose monoclonal antibodies (b) low dose monoclonal antibodies, or (c) a control placebo vaginal microbicide gel. Gels containing either the high or low dose MAbs are referred to as 'the IMP' in this protocol; when referring to either the IMP or the placebo, the term 'study gel' is used. For more details of the study gels refer to section 8. A placebo arm is included in the study to increase the validity of safety endpoint analyses and investigate the impact of the IMP on vaginal flora (see sections 5.5.2 and 12.1).

5.5 Outcome measures

5.5.1 Primary endpoint:

- levels of MAbs in Weck-Cel vaginal secretions 1, 8 and 24 hours post-1st dose, 12 & 36 hours post-12th dose

5.5.2 Secondary endpoints:

- 24 hours post-1st dose, and 36 hours post-12th dose cervico-vaginal lavage MAb levels
- 24 hours post-1st dose, and 36 hours post-12th dose Mab levels in self-sampled vaginal aspirate
- 8 hours post-1st dose, and 12 hours post-12th dose serum MAb levels
- Number of grade 3 or above genital adverse events (see Appendix 6) during the dosing or follow up period
- Number of grade 3 or above other clinical or laboratory adverse (see Appendix 7) events confirmed at examination or on repeat testing respectively during the dosing or follow up period
- Number of events attributable to the study gel leading to discontinuation of gel

Data on local events listed above will be solicited with specific questions and/or cervico-vaginal and colposcopic examination. Data on laboratory events will be collected through the routine tests or in response to a clinical event. Data on other events will be collected with open questions. Detail on the analysis of MAbs in vaginal secretions and serum samples can be found in section 10.4.

5.6 Duration

All study procedures will be carried out over three to four consecutive menstrual cycles. The screening visit will take place between two and six weeks before the first dosing visit. Dosing with the randomised study gel will begin between days 7 and 13 of the second or third menstrual cycle, and participants will administer the study gel once daily for twelve days. Further genital examinations with systemic and genital tract samples will be taken 12 & 36 hours after the last dose. The final follow up visit will take place between days 7 and 21 of the subsequent menstrual cycle.

5.7 Sub-studies

We will conduct three sub-studies relating to the exploratory objectives of the study: 1) investigation of the impact of Mabgel on vaginal flora; 2) comparison of self-sampled, to clinician-sampled vaginal secretions and 3) Investigation of the experience of taking part in the clinical trial. Further details are provided in section 12.2.

5.8 Organisational summary

Co-Sponsors: York Hospitals NHS Foundation Trust and the University of York
Funder: European Commission (European Microbicide Programme)
Co-ordinator: Professor Charles JN Lacey

6. RECRUITMENT OF PARTICIPANTS

6.1 Inclusion criteria

- Females
- Aged ≥18 and ≤45 years
- In good health as determined by medical history, physical examination and clinical judgement
- Willing and able to give written informed consent
- Available for the duration of the study
- Willing to undergo screening for HIV, Hepatitis B & C, and sexually transmitted infections
- Willing to abstain from vaginal practices including receptive oral sex and sexual intercourse from 48 hours before the first dose of the study gel until after visit 7
- If physiologically fertile, using a reliable method of contraception for the 3 to 4 menstrual cycles covering the pre-study, study dosing and follow up cycle to visit 8 (methods defined as one of: consistent use of condoms with every act of sexual intercourse; combined oral contraceptive pill; desogestrel-containing progesterone only pill (Cerazette); intra-uterine contraceptive device or system; injectable contraceptive; progesterone implant)
- Willing to abstain from using any genital preparations, other than the study gel, during the period of gel administration until after visit 7
- Willing to abstain from using tampons during the period of gel administration until after visit 7
- Judged by clinician to be able, and likely, to comply with the procedures required as set out in the protocol
- Have been registered with a GP for at least the past 3 months
- Response received from GP before randomisation
- Have access to a domestic refrigerator at home for the purposes of storing the study gel

6.2 Exclusion criteria

- Untreated syphilis, gonorrhoea, trichomonas, chlamydia, vaginal candidosis or bacterial vaginosis
- Clinically significant (out of the normal range and deemed clinically significant by the CI or study physician) haematological, biochemical, immunological or coagulation assay abnormalities on screening
- HIV infection, anti-HCV antibody positive, HbsAg positive
- Abnormal findings on pelvic examination, deemed clinically significant by the CI or study physician
- History of coagulation or thrombotic disorders
- Significant current general medical illness
- Irregular menstrual bleeding likely to cause vaginal bleeding during the dosing period as judged by the CI or study physician
- Current participation, or participation within the last 2 months in another clinical trial
- Treatment for cervical intraepithelial neoplasia (CIN) or other gynaecological instrumentation of the cervix within the past 3 months
- Pregnant, within 12 weeks postpartum, or breast feeding
- Unlikely to comply with protocol
- Have any condition which, in the opinion of the CI or study physician, might interfere with the evaluation of the study objectives

- Unable to fluently read and speak English to a level adequate for the full comprehension of procedures required in participation and consent

6.3 Number and source of participants

We will aim to recruit and randomise 30 healthy HIV negative female volunteers. All 30 participants will be randomised to one of the three trial arms, subject to complete fulfilment of the eligibility criteria.

Participants will be recruited by advertising in the York area, including via the GU medicine clinic, the University of York, York St John University, mailing lists, and by coverage and advertising in local media.

6.4 Screening procedures and pre-randomisation investigations

6.4.1 Informed Consent

The Investigator has both ethical and legal responsibility to ensure that each participant being considered for inclusion in this study is given a full explanation of the protocol. This shall be documented on a written informed consent form (Appendix 3) that shall be approved by the same Research Ethics Committee (REC) responsible for approval of this protocol. Each informed consent form shall include the elements required by UK Clinical Trials Regulations and FDA regulations in 21 CFR Part 50 and must adhere to the ethical principles that have their origin in the Declaration of Helsinki.

Participants will be provided with a detailed written participant information leaflet no less than 24 hours before attending for the screening visit. A brief version of the participant information leaflet is also available to be used as an optional aid when explaining key information regarding the trial. Participants will also be given a full verbal explanation of the trial before providing informed consent. Once the appropriate essential information has been provided to the participant and fully explained by a Clinical Investigator, and it is felt that the participant understands the implications of participating, the REC-approved written informed consent form shall be signed and dated by both the participant and the Investigator obtaining consent, who must be a physician. The participant will be given a copy of the signed informed consent form; the original will be kept in the trial master file by the Investigator and a copy will be kept in the participant's file. All of the above mentioned activities must be completed before the participant can take part in the trial.

At the point when a potential participant contacts the research team to indicate their initial interest in the study (this will usually be by telephone in response to an advertisement) the Investigator may discuss the availability of the study and the possibility for entry with a potential participant without first obtaining consent. The following general eligibility questions may be discussed prior to conducting informed consent:

- Age
- Sexual activity
- Any current method of contraception
- Availability for the duration of the study
- Menstrual cycle
- General health state

Informed consent must be obtained and documented prior to initiation of any trial-related procedures that are performed solely for the purpose of determining eligibility for research. Answers to the above eligibility questions will be documented as part of the screening visit.

If a procedure is done in anticipation of, or in preparation for, the research, it is considered to be part of the research.

6.4.2 Screening Visit

Each participant will be assigned a Screening Identification Number (SID). SID numbers will not be reassigned. A master log of screened participants with SID, demographic and locator information must be maintained and kept in the trial master file (kept at the study site in a secure location) to track participants who have been screened for the study. If the participant does not enroll in the dosing part of the trial, study staff will document the reason for not enrolling in the participant register.

At the screening visit, a Clinical Investigator will conduct a general discussion of the trial and the participant's interest in trial participation will be assessed. If the Clinical Investigator determines the participant is eligible (based on the general discussion of the trial and the eligibility criteria and subject to screening) informed consent will be obtained from the female participant by a study physician. Informed consent will be taken prior to the initiation of any trial-related procedures using the informed consent form (Appendix 3). Checks will be made by the study team to prevent over-volunteering.

Once written informed consent is obtained, the following procedures will be conducted according to the Study Schedule (section 7.1):

- Medical, gynaecological, and sexual history
- Details of concomitant medications and allergy history
- Physical examination
- Urine pregnancy test and urinalysis
- HIV, Syphilis, haematology, chemical pathology
- Cervico-vaginal examination, modified colposcopy,and genital infection screen

A record of the above procedures and any findings will be entered into the participant's file.

Following the screening visit, the blood and cervical GC and CT samples will be sent to the pathology laboratory at York Hospital for analysis (the urinalysis, pregnancy test and microscopy for BV, TV and Candida will be carried out at the trial site). During cycle 1 a Clinical Investigator will review the results from the laboratory to determine if participants are eligible for enrolment into the study. Once the laboratory results are obtained, they will be signed and dated by the Investigator within 72 hours of receipt, and the original signed report will be kept in the participant's file.

6.4.3 Screen failures

If the HIV/Syphilis, Hepatitis B or C tests are positive, the participant is not eligible for the trial and will be immediately considered a screen failure. The participant will be given the result (independent of his/her partner unless otherwise specified by the participant) by an appropriate Clinical Investigator and provided with the opportunity for same day referral to a GU physician.

If the pregnancy test is positive, the participant is not eligible for the trial and will be immediately considered a screen failure. The participant will be given the result by an appropriate Clinical Investigator and provided with the opportunity for referral to Family Planning services if appropriate.

If the results for STIs are positive for Chlamydia, gonorrhoea, Trichomonas vaginalis, vaginal candidosis or bacterial vaginosis the participant will be offered the opportunity to be referred to GUM services within 48 hours of results or given the choice to be treated and managed

by a study physician.. If the relevant infection is properly treated with acceptable contact tracing parameters, then the participant will be offered the opportunity to continue with their participation in the study after at least one further menstrual cycle has elapsed. In this case the participant will be assigned a different SID upon re-entry into the trial and the screening visit will be repeated.

If any of the routine screening blood test results are outside normal range (as defined by current York Pathology laboratory ranges) and are deemed to be clinically significant by the CI (or study physician in accordance with the delegation log) the participant will be asked for permission to inform her GP. Similarly, if any abnormalities are detected on genital or physical examination and are deemed to be clinically significant by the CI (or a study physician in accordance with the delegation log) permission to inform the participant's GP or another appropriate physician will be sought.
Depending on the abnormality, its natural history and clinical significance, the participant may be enrolled into the dosing stage of the trial, undergo further investigations, or be considered a screen failure, as determined by the CI (or study physician according to the delegation log). The participant may be assigned a different SID if enrolled after further investigation and/or resolving of the abnormal finding, in which case the screening visit will be repeated.

If for any reason the participant is deemed a screen failure, the participant will be contacted and a final visit will be scheduled to discuss the results. Decisions about suitability of the participant in light of any abnormal results or findings will be fully documented in the participant's file.

6.4.4 Signing off Eligibility

The CI (or study physician in accordance with the delegation log) must confirm eligibility for each participant based on the screening procedures, including findings from clinical histories, examinations and laboratory results, and this must be documented in the participant's file. If participants are deemed to be eligible the first dosing visit (Visit 2) can be scheduled. The participant will contact the research team on the first day of her menses. An appointment for visit 2 will be scheduled for a Wednesday or Thursday on day 7-13 of her cycle.

7. ASSESSMENTS AND FOLLOW-UP

7.1 Schedule for follow-up

The schedule of visits, timing with regard to the menstrual cycle, study days relative to the first dose, and investigations are presented in Table 7-1 below.

7.1.1 Women without a regular menstrual cycle

The schedule has been constructed in the traditional manner, referring to days of the cycle, as seen in a regularly menstruating woman. However, there may be some women, who are using either progesterone injections, progesterone implants, or intra-uterine progestogen-only systems as contraception, who are amenorrhoeic, have no or very infrequent bleeding, and who are thus not in regular cycles. In these women we will arbitrarily define the day of the first dose and length of 'cycle', but using the same intervals as defined in Table 7-1.

7.1.2 Time window for sampling

The study schedule and following description of the visits indicates the timings for sampling to be carried out. This is an approximate time only, and the following time windows for sampling visits are permitted:

- Visits 1, 5 and 8: Sampling must be carried out on the day. There is no restriction on the time at which samples should be taken.
- Visit 2 (pre-1st dose sample): Sampling must be carried out on the day, prior to the delivery of the 1st dose.
- Visit 2 (1 hr post-1st dose sample): Sampling must be carried out within 15 minutes less and 30 minutes more than the time indicated.
- Visits 3, 4, 6 and 7 must be carried out within 1 hour (either more or less) than the time indicated.

The time at which all samples for the pharmacokinetic parameters are taken will be recorded in the participant's file.

Table 7-1 : Schedule of visits

		Screening cycle	Dosing cycle										Follow-up cycle	
**Visit number**		**1**	**t/c**	**2 1st dose**		**3**	**4**	**t/c**	**5**	**t/c**	**6**	**7**	**t/c**	**8**
**Day of menstrual cycle** (Day 1 = first day of bleeding)			1	7-13 W-Th	7-13, W-Th	7-13, W-Th	8-14, Th-Fr	8-16	11-19 M-T	14-22	19-25	20-26	1	7-21
**Study day relative to first dose**		-42 to -14	-6 to -12	1	1	1	2	2-4	5-7	8-10	13	14		
**Time of visit**				pre-1st dose	1hr post-1st dose	8 hrs post-1st dose	24 hrs post-1st dose				12 hrs post-12th dose	36 hrs post-12th dose		
Informed Consent		x												
Randomisation				x										
Medical & other history		x												x
Physical examination		x												x
Cervico-Vaginal examination		x		x	x	x	x		x		x	x		x
Colposcopy		x		x	x	x	x		x		x	x		
GU infection screen	CT cervical NAAT	x												
	GC cervical culture	x												
	Vaginal pH	x										x		x
	Gram stain cervix	x												
	Gram stain vagina	x										x		x
	Wet mount	x												
	Candida culture			x										
	HIV antibody test	x												
	Syphilis serology	x												
Laboratory parameters	Pregnancy test[a]	X		x										x
	Urinalysis[a]	x												
	Biochemistry [b]	x									x			x
	Haematology [c]	x									x			x
	Hepatitis B/C serology	x												
	APTT/aCL [d]	x		x		x					x			x
Pharmacokinetic parameters (for MAbs)	Serum sample[e]			x		x					x			
	Vaginal self-sample[e]	x					x					x		
	Vaginal Weck-Cel[e]			x	x	x	x				x	x		
	CVL supernatant & pellet[f]			x			x					x		
Vaginal swab for vaginal flora substudy[g]		x										x		x
Side-effect, compliance check								x	x	x	x			
Qualitative interview														x
Telephone call for appointment			x										x	
Diary card issued/collected				x							x			

a) Urine dipstick carried out at clinical site; **b)** Sample sent to York Hospital Pathology lab for analysis: U&Es, LFTs, electrolytes. c**)** Sample sent to York Hospital Pathology lab for analysis: Full blood count; **d)** Sample sent to York Hospital Pathology lab for analysis; e**)** Samples processed and stored at HYMS EMU, or at IIU, York; assayed in Polymun Scientific, Vienna; f**)** Samples processed and stored at HYMS EMU, or at IIU, York; supernatant assayed in Polymun Scientific, Vienna, pellet assayed at HYMS EMU, or at IIU, York **g)** Samples processed and stored at HYMS EMU, or at IIU, York; assayed in Institute of Tropical Medicine, Antwerp

7.2 Visit 2: Randomisation and trial entry, pre- and post-1st dose

7.2.1 Pre-1st dose procedures

All screening procedures will be carried out at visit 1, according to the study schedule, before randomisation, allocation of a subject number or administration of the study gel.

At visit 2, to confirm the participant is suitable for randomisation, a Clinical Investigator will review the CRF and screening results. They will check the informed consent documentation, the inclusion and exclusion criteria and that the participant has been signed off as eligible..

Providing the pre-dosing, visit 2 cervico-vaginal examination, colposcopy and pregnancy test are normal, the participant will proceed onto randomisation and the administration of the 1st dose.

7.2.2 Randomisation

Participants entered into the study will be allocated consecutive subject numbers from 01 to 30 at visit 2. The subject number assigned will be recorded on the participant log in the trial master file. This subject number will be used to identify the participant throughout the trial and will be used on CRFs, samples for external analysis and study documentation (see section 11.1). Subject numbers will not be reassigned.

Further details on the randomisation process can be found in section 10.3.

7.2.3 Administration of 1st dose

A Clinical Investigator will transfer the study gel from the syringe (labelled with individual subject number) to an Ortho vaginal applicator and then administer the study gel to the participant at the clinical unit.

7.2.4 Demonstration and instruction on using the gel

A Clinical Investigator will provide each participant with verbal instructions on how to self-administer the study gel, and will describe the process of application while administering the first dose of the study gel. Each participant will be provided with a 'practice' syringe and Ortho vaginal applicator. After a demonstration by the Clinical Investigator, the participant will practise transferring the study gel from the syringe into the applicator under supervision during the clinic visit.

The participant will be provided with the remaining 11 doses in a plastic zipper bag and 11 Ortho vaginal applicators, along with a spare bag for the purpose of returning used syringes. The remaining 11 doses will be self-administered on a daily basis at home. The participant will be given written instructions on how and when to administer the study gel, storage instructions for the study gel and contact details for the research team in case of emergency or any problems (Appendix 5). The participant also will be provided with a diary card for daily completion during the dosing stage of the study. The Clinical Investigator will discuss the circumstances when the participant should contact the research team (due to particular or serious side effects). The participant will be informed that they can contact the research team at any time if they have any concerns or questions.

The demonstration and instruction on using the gel and the provision of the remaining 11 doses of study gel and the diary card can occur at any time on the day of the first dose, as appropriate in each case.

7.2.5 1 hour post-1st dose

One hour after administration of the first dose, investigations will again be carried out according to the study schedule. The Investigator will enter the data collected into the

participant's file and send all samples for processing and storage at HYMS EMU, or at IIU, York.

7.3 Visit 3, 8 hours post 1st dose

The participant will return to the clinical unit for visit 3 eight hours after administration of the first dose. Investigations will be carried out according to the study schedule. The Clinical Investigator will enter the data collected into the participant's file and send all samples for processing and storage at HYMS EMU, or at IIU, York.

7.4 Visit 4, 24 hours post 1st dose

Twenty-four hours after administration of the first dose, investigations will be carried out according to the study schedule. The Clinical Investigator will enter the data collected into the participant's file and send all samples for processing and storage at HYMS EMU, or at IIU, York.

7.5 Dosing at home

The participant will administer doses 2 to 12 at home in the evening. The research team will offer to send a text message to the participant as a reminder at an agreed time on each day when they are due to administer a dose. If the participant does not have a mobile phone, alternative arrangements will be made.

7.6 Telephone calls

A Clinical Investigator will telephone the participant on two occasions during the dosing period (day of the 2nd to 4th and 8th to 10th doses). The Investigator will check on the participant's condition, record information about any adverse events experienced or medication used and check they are using the gel on a daily basis. The Investigator will enter the data collected into the participant's file.

7.7 Visit 5, day of the 5th to the 7th dose

On the day of the 5th to the 7th dose, the participant will attend the clinical unit and examination will be carried out according to the study schedule. A Clinical Investigator will enter the data collected into the participant's file.

7.8 Visits 6 and 7, timed post 12th dose

The participant will be instructed to administer their final dose at a specified time in order to coincide with the time of visits 6 and 7, which will be carried out at 12 and 36 hours after the final dose.

At each visit, procedures will be carried out according to the study schedule, a Clinical Investigator will enter the data collected into the participant's file and send all samples for processing and storage at HYMS EMU, or at IIU, York or the pathology laboratory at York hospital as appropriate.

At visit 6 or 7, all used (and any unused) syringes will be collected by the Investigator for later checking as described in section 8.6. The diary card will be collected.

7.9 Visit 8: Final visit

At the onset of menses after the dosing period, the participant will contact the research team and schedule an appointment for visit 8. Visit 8 will take place on day 7-21 of the participant's menstrual cycle.

Procedures will be carried out according to the study schedule. A Clinical Investigator will enter all data collected into the participant's file and send samples for processing and storage at HYMS EMU, or at IIU, York and the pathology laboratory at York hospital. No arrangements for further follow up will be made unless deemed necessary by the CI.

7.9.1 Qualitative interview

At the final follow up visit, each participant will be interviewed by an appropriately trained Investigator. This qualitative interview will explore participant's views on taking part in the clinical trial and will be conducted according to a pre-prepared topic guide. The Investigator will describe the purpose of the interview, and explain that the interview will be entirely confidential. The interview will be conducted anonymously; no personal identifiers or subject numbers are used to identify recordings or transcripts (a separate, unlinked interview ID will be used). Participants will be informed that quotes from the interview may be reported and/or published, but that they will not be identifiable from these quotes. The process of obtaining informed consent will include reference to this. The audio-recording of the interview will be transcribed before analysis. See section 12.3 for further details.

7.10 Unblinding individual participants

In the event of a SAE the Sponsor may unblind the participant with regard to the allocation treatment group, in order to assign likely causality (whether it is likely to be linked to the IMP or not). This information will not be reported to the research team or CI as a matter of course, in order to maintain double-blinding.

In the event of a SAE occurring during the dosing period (or follow up), it may be considered necessary to completely unblind the participant with regard to the allocated treatment group. We believe this is most unlikely, and in general such unblinding is to be discouraged. Such a decision can only be taken by the CI after careful consideration of the clinical issues related to the SAE, and such a decision would be supported if it could be argued that knowledge of the allocated treatment group would assist in management of the SAE. Such unblinding would be carried out by liaison between the CI and the York Trials Unit. Unblinding should be carefully recorded and reported to the Sponsor.

7.11 Co-enrolment

Participants may not be co-enrolled in any other concurrent clinical studies.

8. DETAILS OF STUDY PRODUCTS

8.1 Products

For this study the MAbs are formulated in a gel vehicle suitable for vaginal application. The formulation of the gel vehicle was developed within the EMPRO project. HEC (Hydroxyethyl Cellulose) was chosen as gel forming matrix. The gel is preserved with parabens (2 mg/g). The placebo gel has the same composition as the active gels, but does not include the monoclonal antibodies.

The constituents of the three study gels are described in Table 8-1 below.

Table 8-1: Study gels

	Composition (per 1G)		
	**Mabgel (high dose)**	**Mabgel (low dose)**	**Placebo**
MAbs	20 mg C2F5<br>20 mg C2G12<br>20 mg C4E10	10 mg of C2F5<br>10 mg of C2G12<br>10 mg C4E10	0
Hydroxyethyl cellulose	16 mg	16 mg	16 mg
Glycerin	25 mg	25 mg	25 mg
Methylparaben	1.8 mg	1.8 mg	1.8 mg
Propylparaben	0.2 mg	0.2 mg	0.2 mg
Maltose	50 mg	51 mg	52 mg
Purified water	847 mg	876 mg	905 mg

The research gels will be supplied by Polymun Scientific, Nussdorfer Laenda 11, A-1190 Vienna, Austria. They will be shipped to the UK as a single batch. Gels will be stored in a refrigerator (2-8°C). Ortho applicators will be purchased from Janssen-Cilag in the UK.

8.2 Packaging and labelling

All study gels are supplied as 3g of gel in a 3ml luer lock syringe. The study gel is transferred from the syringe into an Ortho vaginal applicator immediately before vaginal application. This results in approximately 2.5ml of extractable volume per dose.

Syringes will be individually labelled and each one will be separately packaged. Twelve syringes will be provided per participant and these will be packaged together in a plastic zipper bag. The wording for the labels for each item is shown below (not to scale).

To go directly on syringes (5.5cm x 3.5cm):

3 g Mabgel/placebo **Subject number: (01, 02, 03….30)**
For administration intravaginally
Batch number: HECAK0108
EudraCT number: 2008-000312-32
Investigator: Professor C Lacey
Sponsor: York Hospitals NHS Foundation Trust & University of York

To go on syringe packaging of each individual unit (7cm x 5 cm):

Mabgel/placebo **Subject number: (01, 02, 03….30)**

3 g gel for administration intravaginally, contains 20 or 10 or 0 mg/g gel of each mab (C2F5, C2G12, and C4E10). Transfer total expendable contents into an Ortho vaginal applicator and insert into the vagina as detailed in the 'Mabgel - instructions for use' patient information leaflet.

Batch number: HECAK0108
Expiry date: Month/Year
EudraCT number: 2008-000312-32
Investigator: Professor C Lacey, Department of GU Medicine, York
Sponsor: York Hospitals NHS Foundation Trust & University of York

Store at 2-8°C (in the refrigerator)

To go on the outer package (plastic zipper bag), 12 syringes packed together (7cm x 7.5cm):

Mabgel/placebo **Subject number: (01, 02, 03….30)**

For clinical trial use only
NOT FOR INJECTION

3 g gel per syringe for administration intravaginally, contains 20 or 10 or 0 mg/g gel of each mab (C2F5, C2G12, and C4E10). Transfer total expendable contents into an Ortho vaginal applicator and insert into the vagina as detailed in the 'Mabgel - instructions for use' patient information leaflet.

Batch number: HECAK0108
Expiry date: Month/Year
EudraCT number: 2008-000312-32
Investigator: Professor C Lacey, Department of GU Medicine, York
Sponsor: York Hospitals NHS Foundation Trust & University of York

Contains 12 doses
Store at 2-8°C (in the refrigerator)

Keep out of reach of children

8.3 Storage of the gels

The study gels will be stored in a refrigerator (2-8°C) at the trial site, until dispensed to the participant. This refrigerator will be in a secure facility with restricted access. Participants will be instructed to store the study gels in a domestic refrigerator at home. The storage

temperature of the gels will be monitored at the clinical site and documented on a temperature log.

8.4 Treatment schedule and dispensing

The study gel will be dispensed by a Clinical Investigator at the trial site. The first dose of study gel will be given in the research clinic. The Investigator will demonstrate and instruct the participant on the procedure for transferring the study gel into the vaginal applicator. A short information sheet will be given to the participant to explain how to prepare and administer the microbicide. The Clinical Investigator will administer the first dose to the participant. When the participant leaves the clinic that day, she will be issued with the subsequent eleven doses and eleven applicators contained in a plastic zipper bag. The participant will be given instructions on how to store the gel safely at home. She will also be provided with a spare plastic zipper bag for the purpose of returning used syringes. All subsequent doses will be administered at night at bedtime, except for the 12th dose, which will be administered at a specified time to coincide with visit 6.

8.5 Dose modifications

Interruption of dosing or permanent discontinuation due to adverse events is not expected. Details of the management of adverse events that could lead to such interruption or discontinuation are provided section 9. The toxicity grading is included as Appendices 6 and 7.

8.6 Accountability and unused drugs/devices

The research team will keep a log of the receipt of all the investigational materials, and also logs detailing the issue of vaginal gels and materials to the participants, and the receipt and checking of use of the returned gels. Any unopened, unused study gel in syringes remaining after the end of the trial will be returned to Polymun Scientific, Vienna.

All used syringes will be retained by the participant and returned to the clinic at visit 6 or 7. After visual inspection as detailed below, all used syringes will be stored at the trial site until after the trial monitor has verified their return, and will subsequently be disposed of as clinical waste.

8.7 Measures of compliance and adherence

Compliance/adherence will be assessed by history and by inspection of returned syringes. An Investigator will assess whether the syringe is fully or partially emptied or not emptied at all. This data will be recorded in the participant's file.

8.8 Non-trial treatment

8.8.1 Medications permitted

Hormonal contraception as defined in section 6.1 is permissible. Routine regular medications such as inhalers or non-steroidal anti-inflammatory agents are only allowable at the discretion of the CI, and provided that this does not mean that the participant fulfils the exclusion criteria of significant current general medical illness. Participants may take paracetamol as deemed necessary. All concurrent medications and decisions regarding eligibility in light of medication will be documented in the participant's file.

8.8.2 Medications not permitted/precautions

Participants may not use tampons or any genital preparations such as pessaries, creams, etc, during the period of gel administration until after visit 7, unless prescribed after evaluation by a study physician. Participants must abstain from sexual intercourse and receptive oral sex from 48 hours before gel administration until after visit 7.

8.8.3 Data on concomitant medication

All concomitant medication must be recorded in the participant's file and updated at each visit.

9. SAFETY REPORTING

The North and East Yorkshire Research and Development Alliance is responsible for coordinating the reporting of SAEs in accordance with the UK Medicines for Human Use (Clinical Trials) Regulations

For all adverse events, the Investigators will take appropriate action to ensure the safety of all participants and staff in the study. The Investigators will consider what actions, if any, are required and in what timeframe. Decisions about interruption or discontinuation of dosing will be taken in line with section 9.4.

All adverse event reporting will be carried out in accordance with the North and East Yorkshire R and D Alliance Research Related Adverse Event Reporting Procedures.

9.1 Definitions

**Term**	**Definition**
**Adverse Event (AE):**	Any untoward medical occurrence in a subject to whom a medicinal product (in this case, study gel) has been administered, including occurrences which are not necessarily caused by or related to that product.<br><br>*An AE can therefore be any unfavourable and unintended sign (including an abnormal laboratory finding), symptom, or disease* ***temporally*** *associated with the use of an investigational medicinal product (IMP), whether or not considered related to the IMP.*<br><br>*Note: The above definition has been taken from the Medicines for Human Use (Clinical Trials) Regulations 2004. However for the avoidance of doubt, in line with the North and East Yorkshire R and D Alliance Research Related Adverse Event Reporting Procedures, all AEs will be collected for all trial subjects from the time of their enrolment into the study, whether an IMP has been administered to the subject or not.*
**Solicited Genital Adverse Event (AE):**	One of the specified adverse events that will be systematically solicited during visits. These events are listed by grade in Appendix 6.
**Adverse Reaction (AR):**	Any untoward and unintended response in a subject to an IMP related to any dose administered to that subject.<br><br>*All AEs judged by either the reporting investigator or the Sponsor for which there is at least some evidence or argument to support a causal relationship to a medicinal product qualify as adverse reactions.*<br>*Note: All adverse reactions are adverse events*
**Unexpected Adverse Reaction:**	An AR, the nature or severity of which is not consistent with the applicable product information in the investigator's brochure.
**Serious Adverse Event (SAE)** or	Any adverse event or adverse reaction that at any dose:<br>• Results in death

**Serious Adverse Reaction:**	• Is life-threatening*<br>• Requires hospitalisation, or prolongation of existing inpatients' hospitalisation**<br>• Results in persistent or significant disability or incapacity<br>• Is a congenital anomaly or birth defect
**Suspected Unexpected Serious Adverse Reaction (SUSAR):**	Any suspected adverse reaction related to an IMP that is both unexpected and serious. There are no serious adverse reactions for MAbs C2F5, C2G12 and C4E10 or the gel vehicle reported to date and so any such event would be unexpected.

9.1.1 Clarifications and Exceptions

*The term 'life-threatening' in the definition of 'serious' refers to an event in which the participant was at risk of death at the time of the event; it does not refer to an event which hypothetically might have caused death if it were more severe.

**Hospitalisation is defined as an inpatient admission, regardless of length of stay, even if the hospitalisation is a precautionary measure for continued observation. Hospitalisations for a pre-existing condition (including elective procedures that have not worsened) do not constitute an SAE.

Medical judgement should be exercised in deciding whether an AE/AR is serious in other situations. Important AE/ARs that are not immediately life-threatening or do not result in death or hospitalisation but may jeopardise the participant or may require intervention to prevent one of the other outcomes listed in the definition above, should also be considered serious.

9.2 Causality

The assignment of the causality should be made by the CI, or Clinical Investigator as delegated by the CI, using the definitions in the table below. The Investigator will use clinical judgement to determine the relationship. Alternative causes, such as underlying illness, concomitant therapy, other risk factors etc. will be considered. The Investigator should also consult the Investigator Brochure.

Relationship	Description	Event type
**Not related**	There is no evidence of any causal relationship. The temporal relationship of the onset of the event, relative to administration of the IMP, is not reasonable or another cause can by itself explain the occurrence of the event.	AE/SAE
**Unlikely**	The temporal relationship of the onset of the event, relative to administration of the IMP, is reasonable but is more likely to have another cause which can by itself explain the occurrence of the event.	AE/SAE
**Possibly Related**	The temporal relationship of the onset of the event, relative to administration of the IMP, is reasonable but the event could have been due to another, equally likely cause.	AR/SAR/ SUSAR
**Probably Related**	The temporal relationship of the onset of the event, relative to administration of the IMP, is reasonable and the event is more likely explained by the product than any other cause	AR/SAR/ SUSAR

**Definitely Related**	The temporal relationship of the onset of the event, relative to administration of the IMP, is reasonable and there is no other cause to explain the event.	AR/SAR/ SUSAR
**Not assessable**	There is insufficient or incomplete evidence to make a clinical judgement of the causal relationship.	

9.3 Reporting procedures

The Investigator will complete an SAE report form and forward by fax to the North and East Yorkshire R and D Alliance immediately (within 24 hours) of becoming aware of an SAE.

Contact details for reporting SAEs and SUSARs

In the case of a SAE, complete a SAE report form and forward by fax to the North and East Yorkshire R&D Alliance within 24 hours of becoming aware of the SAE.

Fax numbers:
North and East Yorks R&D Alliance: 01904 731297

9.3.1 Follow-up

In the event of an SAE, the participant will be followed up by the Investigator until completion of the event or until a decision is made by the Sponsor to cease follow up.

In the event of withdrawal due to an SAE or AE, the Investigator will follow up the participant until completion of the event or until a decision is made by the Sponsor to cease follow up.

9.3.2 Ongoing and end of trial reporting

At the conclusion of the study all AEs/ARs, occurring during a study must be subject to statistical analysis and that analysis and subsequent conclusions will be included in the final study report.

One year following the granting of a Clinical Trials Authorisation Certificate, and thereafter annually, the CI will send an annual safety report to the:

- Sponsor
- Medicines and Healthcare products Regulatory Agency (MHRA).
- Research ethics committee that granted approval.

In addition, the CI will provide annual progress reports to the research ethics committee and report to the Sponsor as required.

9.3.3 Pregnancy reporting

Should a pregnancy occur in a participant during the trial then this pregnancy will be reported to the Sponsor as an SAE and the pregnancy will be followed up by the CI in accordance with North and East Yorkshire R and D Alliance procedure.

9.4 Withdrawal of participants from the study

The participant is free to withdraw from the study at any time, without giving a reason, and without their clinical care being affected.

9.4.1 Interruption of dosing

Interruption of dosing describes a temporary cessation of study gel use. Interruption of dosing will occur in the presence of:

- a grade 2 or above solicited genital adverse event
- any clinical event at the discretion of the CI which should lead to discontinuation of study gel

Dosing may be recommenced after interruption following an adverse event at the discretion of the CI.

9.4.2 Discontinuation of dosing

Discontinuation of dosing describes a permanent cessation of study gel use. Discontinuation of dosing will occur in the presence of:

- a grade 3 or above solicited genital adverse event
- a grade 3 or above clinical or laboratory event
- any clinical event at the discretion of the CI which should lead to discontinuation of study gel

Interruption or discontinuation of gel use may also occur if the participant desires to stop study gel use. Wherever possible, participants who discontinue study gel use either by choice or following advice will continue to adhere to the follow up schedule up to the final visit.

9.4.3 Withdrawal from the trial

Withdrawal from the trial means discontinuation of both study visits and study gel. The reasons for withdrawal (if available) and any efforts made to contact the participant after loss of contact or non-attendance should be recorded in the participant's file. Examples for study withdrawal include:

- participant desire to stop participating in the trial
- Investigator concerns that continuing visits is not in the best interest of the participant
- decision taken to discontinue the trial in all participants

9.4.4 Samples and analysis after withdrawal

If a participant is considered a screen failure, discontinues dosing or withdraws from the study at any time, either by choice or on the recommendation of clinical personnel, data and samples collected up to that point will remain available for analysis as part of the study. Participants' anonymised tissue samples will be stored and analysed as per the study protocol.

9.4.5 Follow-up of participants after withdrawal

In the event of withdrawal due to an SAE or AE, the Investigator will follow up the participant until completion of the event.

9.5 Temporarily halting or stopping the study

In the case of 3 women experiencing either a grade 3 or above genital adverse event or grade 3 or above clinical or laboratory adverse event, the study will be temporarily halted pending unblinded review by the Data Monitoring Committee (DMC). Several severe or

serious AEs, which are probable in relation to the study gel, will lead to the study being terminated at that point. The decision to stop the study rests on the CI, with recommendations from the DMC (see section 11.6.3).

The Sponsor reserves the right to terminate the study at any time.

10. STATISTICS AND ANALYSIS

10.1 Sample size

As this is the first administration in humans intravaginally, there is no information to carry out power calculations. This study will provide some information to help such calculations in future studies.

10.2 Planned statistical analyses

Analysis will be carried out according to a detailed statistical analysis plan.

10.2.1 Initial subject characteristics

All demographic (age, height, weight) and other baseline subject characteristics (physical examination, concomitant diseases) will be tabulated and analysed descriptively.

10.2.2 Statistical analyses of primary and secondary endpoints

Antibody levels in vaginal Weck-Cel samples, cervico-vaginal lavage and serum samples will be summarised for each treatment group as minimum, median, and maximum at each time-point. Treatment groups will be compared using the Kruskal Wallis test corrected for ties. Where the Kruskal Wallis test is statistically significant, $P<0.05$, the two active treatment groups will be compared using a Mann Whitney U test, to check whether there is evidence that the higher dose produces higher antibody concentrations.

Half-life will be estimated for each antibody using only the groups which received an active dose, and observations at 1, 8, and 24 hours after the first dose. The method will be to assume an exponential decay curve. If we cannot assume this, a half-life is not a meaningful estimate. A log transformation of the observed concentration will be used and an analysis of covariance model fitted with time as continuous predictor and participant as a categorical factor. Two checks on the assumptions will be made. Linearity will be checked by including and testing a time squared term. Uniformity of the half life will be checked by testing an interaction between time and participant. If the assumptions are met, half-life will be estimated by log(2)/slope. The 95% confidence interval will be found from the usual confidence interval for the slope. If assumptions are not met, analysis will then be data-driven.

All analyses of antibody levels will be carried out using the raw data and Weck-Cel antibody concentrations will also be analysed using data adjusted for the dilution factor. All "not detectable" observations will remain "not detectable" and will not be adjusted. The dilution factor will be calculated as

(volume of processing buffer + Weck-Cel sample volume) / Weck-Cel sample volume

Adjustment for the dilution factor will be to multiply the observed antibody concentration by this dilution factor. Volume of processing buffer will be 600 microlitres.

Analysis of the safety endpoints will involve comparing counts of adverse event rates in the three groups using negative binomial regression.

Comparisons will be made for the following:

- Number of grade 3 or above solicited genital adverse events or events attributable to the study gel leading to discontinuation of gel (Appendix 6) during the dosing or follow up period

- Number of grade 3 or above other clinical or laboratory adverse (Appendix 7) events confirmed at examination or on repeat testing respectively during the dosing or follow up period
- All adverse events (irrespective of assigned relationship to study gel)
- All adverse events classed as possibly or probably treatment-related.

Details of sub-study analyses are provided in section 12.

10.3 Randomisation scheme and maintenance of blinding

10.3.1 Randomisation code

Randomisation will be carried out by the York Trials Unit, University of York. Subject numbers 01 to 30 will be randomised to Mabgel (high dose), Mabgel (low dose) or placebo using blocked randomisation of mixed block sizes. Randomisation will be in the ratio of 1:1:1. The randomisation program will be generated using Microsoft Visual Basic and a copy of the generated data stored in Microsoft SQL Server. The randomisation list will be posted to Polymun Scientific, Austria for the purposes of labelling the study gel. Personnel carrying out subsequent analyses will not have access to this list.

Participants will only be allocated a randomised subject number when they have provided informed consent to take part in the trial, and all screening tests have been carried out to ensure they meet the eligibility criteria. Subject numbers will then be allocated on a sequential basis at visit 2 after cervico-vaginal examination.

In order to maintain double-blinding, neither the CI nor the Investigators will have direct access to the randomisation codes.

10.3.2 Emergency unblinding procedure

Randomisation break codes will be held by Polymun Scientific and the York Trials Unit, University of York.

It is the responsibility of the CI to decide when participants may be unblinded. At the beginning of the trial, the Sponsor and CI will be provided with a password for the online unblinding service provided by the York Trials Unit. In the event of unblinding being required the CI (or designated clinician) will use the password-protected, online unblinding service. Unblinding by the Investigator should only take place in the event of a medical emergency, and efforts should be made to maintain Investigator blinding at all times.

Each participant will carry a card (similar size to a credit card) that indicates that the bearer is taking part in a clinical trial, along with the contact details for the research team. The participant will be instructed to carry this at all time to aid communication with the research team in the event of admission to hospital or an emergency situation.

10.3.3 Sponsor unblinding of SAEs

In the event of an SAE, the Sponsor may unblind participants, without informing the Investigator of the treatment group. If unblinding is required, the Sponsor will use the password-protected, online unblinding service provided by the York Trials Unit.

10.4 Analysis of biological samples

Serum and vaginal secretion samples will be analysed according to established SOPs (held by Polymun Scientific, Vienna, the Institute of Tropical Medicine and IIU, York). A brief description is provided below:

10.4.1 C2F5 content (ELISA)

C2F5 content is determined by an ELISA system using the peptide GGGLELDKWASL for coating and goat anti-human IgG gamma chain antibody (Zymed Laboratories Inc. 62-8420, USA) conjugated with horseradish peroxidase for detection. A highly purified internal C2F5 antibody is used as a standard.

Quantification is performed in pre-coated 96-well microtiter plates. The standard and samples are run in a 2^8 fold dilution row. The concentration of the standard is in the range of 200 - 1.563 ng/ml. After incubating the pre-coated plates with the added samples for one hour at room temperature and washing with PBS pH 7.2 including 0.1 % Tween 20, the conjugated antibody is added. After incubation for one hour at room temperature and washing with PBS pH 7.2 including 0.1 % Tween 20, the enzymatic colour reaction is started by addition of the chromogene substrate o-phenylenediamine dihydrochloride (OPD) and H_2O_2 and stopped by adding 2.5 M sulphuric acid. The absorbance is measured at 492 nm (reference wavelength 620 nm) and the results are evaluated with a fourth-degree polynomial (Software Biolise for Windows). (Polymun SOP number: AI0018).

The test is considered valid if the well with the highest standard protein concentration displays an absorbance of at least 1.0 and if the absorbance of the blank vials is below 0.1.

10.4.2 C2G12 content (ELISA)

C2G12 content is determined by an ELISA using the protein gp160 for coating and anti-idiotypic antibody M1G1 (in-house) conjugated with horseradish peroxidase for detection. A highly purified internal C2G12 antibody is used as a standard.

Quantification is performed in pre-coated 96-well microtiter plates. The standard and samples are run in a 2^8 fold dilution row. The concentration of the standard is in the range of 200 - 1.563 ng/ml. After incubating the pre-coated plates with the added samples for one hour at room temperature and washing with PBS pH 7.2 including 0.1 % Tween 20, the conjugated antibody is added. After incubation for one hour at room temperature and washing with PBS pH 7.2 including 0.1 % Tween 20, the enzymatic colour reaction is started by addition of the chromogene substrate o-phenylenediamine dihydrochloride (OPD) and H_2O_2 and stopped by adding 2.5 M sulphuric acid. The absorbance is measured at 492 nm (reference wavelength 620 nm) and the results are evaluated with a fourth-degree polynomial (Software Biolise for Windows). (Polymun SOP number: AI0005)

The test is considered valid if the well with the highest standard protein concentration displays an absorbance of at least 1.0 and if the absorbance of the blank vials is below 0.1.

10.4.3 C4E10 content (ELISA)

C4E10 content is determined by an ELISA system using the peptide KKWNWFDITNWGGG for coating and goat anti-human IgG gamma chain antibody (Zymed Laboratories Inc. 62-8420, USA) conjugated with horseradish peroxidase for detection. A highly purified internal C4E10 antibody is used as a standard.

Quantification is performed in pre-coated 96-well microtiter plates. The standard and samples are run in a 2^8-fold dilution row. The concentration of the standard is in the range of 1.563 - 200 ng/ml. After incubating the pre-coated plates with the added samples for one hour at room temperature and washing with PBS pH 7.2 including 0.1 % Tween20, the conjugated antibody is added. After incubation for one hour at room temperature and

washing with PBS pH 7.2 including 0.1 % Tween20, the enzymatic colour reaction is started by addition of the chromogene substrate o-phenylenediamine dihydrochloride (OPD) and H_2O_2 and stopped by adding 2.5 M sulphuric acid. The absorbance is measured at 492 nm (reference wavelength 620 nm) and the results are evaluated with a fourth-degree polynomial (Software Biolise for Windows). (Polymun SOP number: AI0026)

The test is considered valid if the well with the highest standard protein concentration displays an absorbance of at least 1.0 and if the absorbance of the blank vials is below 0.1.

10.4.4 Vaginal flora analysis

Real time PCR of vaginal fluid will be performed for *Lactobacillus species* and for *Gardnerella vaginalis* and *Atopobium vaginae*. DNA will be isolated with magnetic extraction with BOOM-technology (Biomérieux) according to the manufacturer's instructions. Quantitative PCR will be performed with Sybr Green PCR Master Mix. Sequences of *Lactobacillus* species specific primers are selected for *Lactobacillus crispatus*, *Lactobacillus gasseri*, *Lactobacillus jensenii*, *Lactobacillus vaginalis* and *Lactobacillus iners*. Primers described by Byun et al will be used for *Lactobacillus* gasseri and *Lactobacillus* crispatus (40). Primers described by Verhelst et al will be used for *Atopobium* vaginae and *Gardnerella* vaginalis (31). New primers will be set up for *Lactobacillus* gasseri, *Lactobacillus* jensenii and *Lactobacillus* vaginalis.

In addition, quantitative PCR for *Lactobacillus* species will be performed on the pellet taken from the cervico-vaginal lavage.

10.4.5 Coagulation parameters

Blood samples will be sent to the York pathology laboratories to undergo routine testing for APTT and anti-cardiolipin antibodies.

10.4.6 Candida culture

High vaginal swabs will be sent to the York Hospital pathology laboratory to undergo Candida culture, speciation and quantification.

11. MANAGEMENT OF DATA, SAMPLES AND TRIAL PROCEDURES

11.1 Data management

11.1.1 Source data

Consenting participants will be allocated a 'participant's file' which will act as the source data. These notes will be separate from any existing hospital notes or GUM clinic notes, but when an onward referral is made, the hospital/GUM clinic number will be recorded in the participant's file to enable linking to the participant's medical records. An alert label will be placed on any existing York hospital records to indicate that the patient is taking part in a clinical trial, and that the research team should be contacted upon admission. Permission will be obtained as part of the informed consent process to allow the research team and other responsible individuals access to the participant's medical records.

Data collected directly from the participant or from medical examinations will be entered onto a proforma to be included in the participant's file. All laboratory reports will be filed in the participant's file after review by a clinical Investigator. Data collected on the proforma will be used to complete the CRFs.

The participant's file will hold personal identifiable information on the participant, including name, address and date of birth, along with the subject number. The participant's file will be kept at the clinical site in a secure location. Data collected from the interviews will be analysed anonymously, and as such these data will not be entered into the participant's file, although it will be noted in the participant's file that an individual has been interviewed.

11.1.2 Case report forms and study database

Data collected at the clinical site will be transcribed onto case report forms (CRFs). The type of data to be recorded in the CRF will be in line with details provided in section 7.1. Appendix 8 provides details of what constitutes source data in this trial. CRFs will be identified with subject number, participant initials and date of birth, and no personal identifiable information will be sent outside of the hospital.

Covance will undertake delegated data management responsibilities, which include the provision of a study database, data entry and validation procedures. Completed CRFs will be sent to Covance and entered into the study database according to established SOPs.

11.1.3 Participant log

A participant log containing SIDs, subject numbers (if enrolled into the dosing stage of the study), participant name and date of birth will be kept in the Trial Master File (TMF). The TMF will be kept in a secure location at the trial site, with access restricted to study staff.

11.1.4 Direct Access to Data

Participating Investigators agree to allow trial-related monitoring, including audits and regulatory inspections by providing direct access to source data/documents as required. Participants' consent for this is obtained as part of the consent process. Where access to CRFs is required, read-only electronic access will be provided.

11.1.5 Archiving of data
All participants' files and CRFs will be securely stored for a minimum of 15 years after the close of the trial.

11.1.6 Laboratory reports
Samples for pharmacokinetic parameters (see section 7.1) will be sent to Polymun, Vienna, for analysis. Results from these analyses will be provided to the trial site and Sponsor in the form of a signed scientific report. These data will be filed centrally in the Trial Master File by study close-out.

Samples for vaginal flora analysis will be sent to the Institute of Tropical Medicine, Antwerp for analysis. Results from these analyses will be provided to the trial site and Sponsor in the form of a signed scientific report. These data will be filed centrally in the Trial Master File by study close-out.

11.1.7 Confidentiality
Participants will be identified by their subject number (or SID), date of birth and initials only on any samples or documentation that leave the trial site. No personal identifiable data will be shared with external organisations. Participants will not be identifiable in any study reports or publications.

11.2 Management of biological samples
Blood samples taken for routine safety parameters, GU infection screen or coagulation parameters will be sent to the pathology laboratory at York Hospital. These samples will be identified by the participant's SID or subject number, date of birth and participant initials only.

Serum samples and vaginal secretion samples taken for pharmacokinetic parameters and vaginal flora analysis will be processed and stored at HYMS EMU, or transported to the Immunology and Infections Unit, University of York for processing and storage according to relevant SOPs. These samples will be identified by subject number (or SID), date of birth and participant initials only. Processed samples will be transported to Polymun Scientific and the Institute of Tropical Medicine (ITM) for analysis.

11.3 End of the study
The end of the study is defined as the date of the last visit of the last participant undergoing the trial (visit 8 of the final participant). The CI will notify the MHRA of the end of the trial within 90 days of its completion, or within 15 days if the study is terminated early.

11.4 Risk assessment
A full trial risk assessment has been carried out in collaboration with the North and East Yorkshire Alliance

11.5 Trial monitoring
The conduct of the trial will be formally monitored by Covance. Monitoring will be undertaken before any participants are enrolled in the study and at regular intervals by suitably qualified and trained personnel in accordance with their own SOPs. The purpose of monitoring is to ensure:

- compliance with the protocol
- adherence to regulatory and GCP obligations
- proper maintenance of all study documentation
- the completeness and exactness of the data entered on the CRFs
- source data verification
- accurate reporting of all adverse events
- drug accountability
- close liaison with the Investigators to clarify any problems which may arise during the study

11.6 Trial management

11.6.1 Delegation of responsibilities

The Sponsor delegates certain roles and responsibilities to the CI, as detailed in the SOP entitled 'Delegation of Roles & Responsibilities for Alliance Trust Sponsored Clinical Trials of Investigational Medicinal Products (CTIMPs)'.

11.6.2 Trial Management Group

A Trial Management Group (TMG) will be formed comprising the CI and other Investigators based at the study site. The TMG will be responsible for the day to day running of the trial and will meet at least once a month during the course of the trial.

The TMG will provide feedback to the Data Monitoring Committee.

11.6.3 Data Monitoring Committee

The Sponsor will appoint a Data Monitoring Committee (DMC) which will incorporate the functions of data and safety monitoring. Numbers and membership of the DMC will be decided by the Sponsor. The role of the DMC will be to:

- supervise the conduct of the trial on behalf of the Sponsor, having particular regard for the rights, safety and well-being of participants;
- monitor Trial progress and protocol adherence;
- review new information from other studies that may affect the Trial;
- determine if any additional interim analyses should be undertaken;
- consider the trial data and the results of any interim analyses, unblinded if considered appropriate, as well as any safety issues and relevant information from other sources, and make recommendations on continuation of the Trial;
- consider any requests for release of interim data;
- in the event of further funding being required, provide appropriate information and advice on the data gathered to date that will not jeopardise the integrity of the Trial.

12. SUB-STUDIES

12.1 Investigation of the impact of Mabgel on vaginal flora

An important aspect of assessing the local safety of a microbicide is evaluating its effect on the vaginal microflora[40]. This is commonly done by measuring the pH of the vagina, and evaluating the flora with microscopy techniques and culture methods[41-43]. Special attention has been given to *Lactobacillus* organisms for which there is mounting evidence that they play a protective role against STDs[44-45]. A recent nested case-control study showed an association between abnormal vaginal flora and HIV seroconversion[46]. Culture methods can differentiate between H_2O_2 producing *Lactobacillus* species and non- H_2O_2 producing species[42]. *Lactobacillus* species producing H_2O_2 have shown *in vitro* inhibition of the growth of various micro-organisms including HIV[47]. The exact protective mechanism(s) is/are still unknown as is the exact composition of the microbial organisms related to this effect.

The introduction of new molecular biology techniques such as real time polymerase chain reaction (quantitative PCR) has made it possible to detect organisms that are cultivation-resistant[48]. The identification and quantification of single organisms in the vaginal flora is now possible[49-51]. Colleagues at the Institute for Tropical Medicine, Antwerp, Belgium, (Dr Tania Crucitti and Dr Vicky Jespers) have developed these techniques, and we wish to collaborate with them on a small sub-study. We will thus take a single additional vaginal swab and vaginal gram stain (i) before dosing, (ii) at the end of dosing, and (iii) during the next menstrual cycle, to assess any changes in the vaginal flora caused by the use of the microbicides. Dr Jespers and Dr Crucitti will be responsible for carrying out these laboratory analyses.

Lactobacillus species will also be identified from cervico-vaginal lavage samples. While the supernatant from the cervico-vagnial lavage samples will be sent to Polymun Scientific, Vienna for identification of MAbs, the pellet will be stored in the Immunology and Infections Unit, University of York and undergo quantitative PCR for lactobacillus species.

12.2 Comparison of self-sampled, to clinician-sampled vaginal secretions

Weck-Cel samples have been established as effective and reproducible methods of collecting vaginal secretion samples, but require a speculum examination by a clinician. In order to reduce speculum examinations in clinical trials, self-sampling of vaginal secretions would be desirable. A new self-sampling device which obtains neat vaginal secretions has recently been developed, but this has not been validated against Weck-Cel sampling at the present time. If self-sampling is found to be an acceptable and effective method of sampling in this study, future clinical trials of vaginal microbicides would be able to use this method as a primary outcome measure.

Participants will collect neat samples of their own vaginal secretions at three time points (as determined by the study schedule, see section 7.1), using a volumetric vaginal aspirator. Vaginal aspirators will be provided by the Center For AIDS Research, University of North Carolina, Durham, NC.

Levels of MAbs in clinician-collected Weck-Cel samples will be compared to those found in self-collected samples..Provided sufficient data is obtained, regression will be used to see

how well the antibody levels in the clinician-collected Weck-Cel samples can be predicted from those found in the self-collected samples.

12.3 Investigation of the experience of taking part in the clinical trial

This trial involves a relatively intense schedule of visits and investigations. In order to improve the conduct of future clinical trials for vaginal microbicides or similar studies, we will conduct individual qualitative interviews with all participants to explore their experiences of taking part in the trial. This interview will be carried out by an appropriately trained Investigator according to a pre-prepared topic guide. Topics covered during this interview will include, but are not limited to, motivation for involvement in the clinical trial, compliance with the abstinence requirement, suitability of information provided and communication during the trial, use of the self-sampling device, the experience of clinical visits and experience of using the product. The findings from the interviews will be used to aid the development of future clinical trials, and to ensure that participant preferences are taken into account in future studies.

Audio-recordings from the qualitative interviews will be fully transcribed. This will be done anonymously so that no participant identifiers or subject numbers are included. Audio recordings and transcripts will be identified using a separate interview ID number. Transcripts will be analysed in line with the 'framework' approach[54-55]

13. ETHICAL CONSIDERATIONS AND APPROVAL

13.1 Ethical approval

Before commencement of the study, the protocol, consent forms and participant information sheet will be submitted for NHS Research Ethics Committee (REC) approval.

For details of any changes since the original version of the protocol that have been REC-approved, see section 18.

13.2 Informed consent

Prior to the commencement of the study (at least 24 hours before attending for the screening visit), each participant will be provided with a full written participant information sheet giving details of the investigational product, procedures and potential risks involved during the study. Participants will also be instructed that they are free to obtain further information from the Investigator and that they are free to withdraw their consent and to discontinue their participation in the study at any time.

Following reading of the information sheet and discussion of the study with appropriate research staff, participants will sign the study-specific consent form, in the presence of a relevant Investigator witness, who must be a physician, to indicate that they are freely giving their informed consent.

13.3 Confidentiality

No material will be kept on file referring to the study participant by their full name, other than in source documents (participant's file and participant log) at the clinical site. The confidentiality of the participant will be respected and maintained at all times. All study-related information will be stored securely at the clinical site. All participant information will be stored in locked cabinets in areas with access limited to study staff.

Study reports will be identified by SID/subject number only; CRFs and the associated database records will be identified by SID/subject number, participant initials and date of birth. The participants will be told that all study findings will be stored on a computer and handled in the strictest confidence. Participant logs and participant files (which link study numbers to other identifying information) will be stored in a separate, locked file in an area with limited access at the clinical site.

Blood samples taken for routine safety parameters, GU infection screen or coagulation parameters will be sent to the pathology laboratory at York Hospital. These samples will be identified by the participant's subject number (or SID), date of birth and participant initials only.

Serum samples and vaginal secretion samples taken for pharmacokinetic parameters and vaginal flora analysis will be processed and stored at HYMS EMU, or transported to the Immunology and Infections Unit, University of York for processing and storage according to relevant SOPs. These samples will be identified by subject number (or SID), date of birth

and participant initials only. Processed samples will be transported to Polymun Scientific and the Institute of Tropical Medicine for analysis.
Participants' study information will not be released without written permission of the participant, except as necessary for analysis, monitoring by the Sponsor, Sponsor's designated monitors, or regulatory authorities.

13.4 Risks

The Investigators will ensure that the dignity, rights, safety and well-being of participants are given priority at all times.

York Hospital Emergency Department will receive the protocol and information relating to the IMP prior to the study commencing and will be notified of dosing days.

The MAbs C2F5, C2G12 and C4E10 have not been used intravaginally in humans, however clinical experience exists from intravenous infusions of the MAbs:

In the course of 4 clinical trials the antibodies were infused intravenously to 39 human HIV-1 infected subjects. Antibodies were administered in high dose regimens using multiple repeated infusions and patients received doses up to 80 g of antibodies within a period of 16 weeks. The antibodies were extremely well tolerated in all clinical trials and few adverse events were observed. Of note, the rate of adverse events observed was not related to the dose. Laboratory safety parameters (haematology, clinical chemistry and urinalysis) showed either changes within the normal range or no change compared with pre-trial levels in most patients. Possible antibody related adverse events were primarily myalgias (n = 7) and arthralgias (n = 4). A laboratory adverse event with established relationship to the infusion of antibody C4E10 was a mild prolongation (grade 1 or below) in activated partial thromboplastin time (APTT) directly after antibody infusion in high dose. Prolongations were transient and aPTT levels returned to normal upon cessation of MAb treatment and did not lead to possible related AEs.

In this study the antibodies will be applied in a significantly lower dose (~ 1/50) compared to previous studies and therefore antibody related adverse drug reactions are extremely unlikely (section 9.1).

Possible AEs may rather originate from the dosing procedure and the gel vehicle. All vaginal microbicides can cause minor local adverse events such as vaginal discharge, vaginal spotting/bleeding, vulvo-vaginal itching or burning. Therefore such side effects may be observed during this phase 1 trial, but they are usually mild and resolve during or shortly after cessation of dosing.

Blood sampling can sometimes cause bruising and soreness of the arms, or very rarely a blockage of the vein or a small nerve injury which can cause numbness and pain. Normally these problems resolve with time. Some people may faint while blood is being drawn.

Vaginal examinations and taking cervico-vaginal samples can feel uncomfortable for some women and will be very similar to having a smear test. These procedures may sometimes cause spotting. Women may also experience some discomfort and embarrassment at providing a full sexual history and from undergoing vaginal examinations. Experienced and appropriately trained study staff will conduct the vaginal examinations and the utmost care will be taken to preserve participants' dignity at all times.

If the vaginal applicator is inserted too far it may cause a very small bruise or scratch to the cervix. The participant will probably not notice these, but they may be seen during internal examination.

13.5 Benefits

Although the MAbs show potent HIV-1 neutralisation *in vitro*, this is only a phase 1 study and there will be no direct benefit to the female volunteers.

Participants will receive a thorough medical examination and genitourinary infection screen. In the event of any abnormal findings being found, participants will be advised of the best course of action and referred on to appropriate clinicians where appropriate.

Although participants will receive no direct benefit, the results of the study will provide information which will help in the development of an effective vaginal microbicide. It is hoped that people at risk of HIV infection, particularly in developing countries, will ultimately benefit from the study.

13.6 Reimbursement

Participants will be compensated for their time and potential discomfort or inconvenience resulting from the study. Details of compensation are provided in section 16.

13.7 HIV testing

Participants will be required to undergo HIV testing as part of the eligibility screening process. Participants will receive thorough pre-test counselling in line with normal GUM clinic practices.

14. REGULATORY ISSUES

14.1 Required approvals

Current regulatory frameworks for this phase I trial require that the following approvals/registrations must be obtained before commencement of the trial:

- EudraCT registration (2008-000312-32)
- Clinical Trials Authorisation (MHRA approval)
- Ethical approval from an NHS Research Ethics Committee
- Research Governance Approval from York Hospitals NHS Foundation Trust

14.2 GCP and GMP compliance

The trial will be compliant with the UK Clinical Trials Regulations and all study staff will be GCP trained. The study will be monitored and audited in line with GCP requirements and all adverse events will be recorded and reported.

All trial supplies will be manufactured to EU Good Manufacturing Practice (GMP) standards, as detailed in the Investigator's Brochure and IMP Dossier.

15. INDEMNITY

York Hospitals NHS Foundation Trust and the University of York will act as co-Sponsors for the project.

15.1 Negligent harm

Management approval from the North and East Yorkshire R&D Alliance, on behalf of York Hospitals NHS Foundation Trust will be obtained before commencement of the study. All study personnel will either be employed by the NHS or hold an honorary contract. Therefore NHS indemnity with respect to negligent harm will apply.

15.2 Non-negligent harm

Cover for non-negligent harm is not provided under normal NHS arrangements. Cover for non-negligent harm will be provided by the University of York.

16. FINANCE

16.1 Funding

The study is supported by a grant from the European Commission and is being carried out as part of the European Microbicide Project (EMPRO). The University of York will hold the grant on behalf of the clinical trial team.

16.2 Reimbursement for participants

Remuneration for taking part in the study is as follows:

- Clinic visit requiring colposcopy and/or vaginal sample collection: £65 per visit
- Clinic visit not requiring colposcopy and/or vaginal sample collection: £35 per visit
- Gel administered at home: £10 per dose

According to the full study schedule the reimbursement would be as follows:

- Visits 1 and 3 to 6: £65 per visit
- Visit 2 (extended visit, consisting of two sampling sessions)
- Doses 2-12 administered at home: £10 per dose

A participant who completes the study in full will therefore receive £565. Payment will generally be provided at the end of the trial, in the form of a cheque, unless there is good reason to do otherwise.

17. PUBLICATION

The results of the study will be analysed and compiled in a study report and published in a peer-reviewed journal. The results and analyses will be published as soon as possible.

No verbal or written report on the primary or secondary objectives of the trial may be made without the approval of the EMPRO consortium. The CI, Trial Statistician and Principal Investigators will form the basis of the writing group.

If there are named authors on publications, these should include at least the CI and Trial Statistician. If there are no named authors (i.e. group authorship) then a writing committee will be identified that would usually include these people, at least. The ISRCTN that has been allocated to this trial should be attached to any publications resulting from this trial.

The members of the DMC should be listed with their affiliations in the Acknowledgements/Appendix of the main publication.

18. PROTOCOL AMENDMENTS

Version 3.3, 29 March 2010, incorporates changes to the location of processing and storage of samples, the deletion of references to Diary Cards as source data (except for the timing of doses), deletion of reference to entry into CRF of reports from Polymun, changes to the identifiers used for samples at the pathology laboratory of York Hospital, and deletion of references to HYMS EMU as the sole location in York Hospital for volunteer visits.

Version 3.2, 8 Sept 2009, incorporates a change in the timing of the baseline participant (self)-taken vaginal aspirate from pre-dosing at visit 2 to the screening visit (visit 1) for logistical reasons.

Version 3.1, 6 July 2009, incorporates minor changes requested after formal review by the Sponsor.

Version 3.0, 9 June 2009, incorporates a number of changes which have been made as a result of changes to the schedule to extend the time window of the sampling points. In addition, a brief participant information leaflet has been added, changes to the SAE reporting procedures and adverse definitions have been made for the purposes of clarity. Other changes were made as detailed in the relevant notification of substantial amendment.

Version 2.1, 10/10/2008 was amended in line with comments received from Cambridgeshire 1 Research Ethics Committee, after review at a meeting held on the 16th September 2008. Changes consist of amendments to the Patient Information Sheet.

Version 2.0, 25/07/08 was preceded by version 1.0, 25/06/08 (submitted to the York Hospitals NHS Foundation Trust Research and Development Committee).

19. REFERENCES

1. McCormack S et al. Science, medicine and the future: Microbicides in HIV prevention. BMJ 2001; 322: 410-3
2. Turpin JA. Consideration and development of topical microbicides to inhibit the sexual transmission on of HIV. Expert Opinion Investing Drugs 2002;11:1077-97
3. Moench TR et al. Preventing disease by protecting the cervix: the unexplored promise of internal vaginal barrier devices. AIDS 2001;15:1595-602
4. http://www.popcouncil.org/microbicides/CarraDataComplete.html
5. http://www.hptn.org/research_studies/hptn035.asp
6. http://mdp.mrc.ac.uk
7. Klasse PJ, Shattock R, Moore JP. Antiretroviral drug-based microbicides to prevent HIV-! Sexual transmission. Annu Rev Med 2007; Sep 24; epub ahead of print
8. Xu W, Smith-Franklin BA, Li PL, Wood C, He J, Du Q, et al. Potent neutralization of primary human immunodeficiency virus clade C isolates with a synergistic combination of human monoclonal antibodies raised against clade B. J Hum Virol 2001 4;55-61.
9. Trkola A, Kuster H, Rusert P, Joos B, Fischer M, Leemann C, et al. Delay of HIV-1 rebound after cessation of antiretroviral therapy through passive transfer of human neutralizing antibodies. Nat Med 2005;11:615-22
10. Mascola JR, Stiegler G, VanCott TC, Katinger H, Crapenter CB, Hanson CE, et al. Protection of macaques against vaginal transmission of a pathogenic HIV-1/SIV chimeric virus by passive infusion of neutralizing antibodies. Nat Med 2000;6:207-10
11. Muster T, Steindl F, Purtscher M, Trkola A, Klima A, Himmler G, Rüker F, Katinger H. A conserved neutralizing epitope on gp41 of human immunodeficiency virus type 1. J Virol. 1993;67(11):6642-7.
12. Trkola A, Dragic T, Arthos J, Binley JM, Olson WC, Allaway GP, Cheng-Mayer C, Robinson J, Maddon PJ, Moore JP. CD4-dependent, antibody-sensitive interactions between HIV-1 and its co-receptor CCR-5. Nature. 1996 Nov 14;384☹6605):184-7
13. Hemming A, Gram GJ, Bolmstedt A, Losman B, Hansen JE, Ricksten A, Olofsson S. N-linked oligosaccharides of the C-terminal portion of human immunodeficiency virus type 1 gp120 and viral susceptibility to neutralizing antibodies. Arch Virol 1996;141(11):2139-51
14. Calarese DA, Scanlan CN, Zwick MB, Deechongkit S, Mimura Y, Kunert R, Zhu P, Wormald MR, Stanfield RL, Roux KH, Kelly JW, Rudd PM, Dwek RA, Katinger H, Burton DR, Wilson IA. Antibody domain exchange is an immunological solution to carbohydrate cluster recognition. Science. 2003 Jun 27;300(5628):2065-71
15. Stiegler G, Kunert R, Purtscher M, Wolbank S, Voglauer R, Steindl F, Katinger H. A potent cross-clade neutralizing human monoclonal antibody against a novel epitope on gp41 of human immunodeficiency virus type 1. AIDS Res Hum Retroviruses. 2001 Dec 10;17(18):1757-65
16. Zwick MB, Labrijn AF, Wang M, Spenlehauer C, Saphire EO, Binley JM, Moore JP, Stiegler G, Katinger H, Burton DR, Parren PW. Broadly neutralizing antibodies targeted to the membrane-proximal external region of human immunodeficiency virus type 1 glycoprotein gp41. J Virol. 2001 Nov;75(22):10892-905.
17. Verrier FC, Charneau P, Altmeyer R, Laurent S, Borman AM, Girard M. Antibodies to several conformation-dependent epitopes of gp120/gp41 inhibit CCR-5-dependent cell-to-cell fusion mediated by the native envelope glycoprotein of a primary macrophage-tropic HIV-1 isolate. Proc Natl Acad Sci U S A. 1997 Aug 19;94(17):9326-31

18. Crooks ET, Moore PL, Richman D, Robinson J, Crooks JA, Franti M, Schülke N, Binley JM. Characterizing anti-HIV monoclonal antibodies and immune sera by defining the mechanism of neutralization. Hum Antibodies. 2005;14(3-4):101-13.
19. Parren PW, Moore JP, Burton DR, Sattentau QJ. The neutralizing antibody response to HIV-1: viral evasion and escape from humoral immunity. AIDS. 1999;13 Suppl A:S137-62.
20. Hessell AJ, Hangartner L, Hunter M, Havenith CE, Beurskens FJ, Bakker JM, Lanigan CM, Landucci G, Forthal DN, Parren PW, Marx PA, Burton DR. Fc receptor but not complement binding is important in antibody protection against HIV. Nature. 2007 Sep 6;449(7158):101-4.
21. Purtscher M, Trkola A, Gruber G, Buchacher A, Predl R, Steindl F, Tauer C, Berger R, Barrett N, Jungbauer A, et al. A broadly neutralizing human monoclonal antibody against gp41 of human immunodeficiency virus type 1. AIDS Res Hum Retroviruses. 1994 Dec;10(12):1651-8.
22. Purtscher M, Trkola A, Grassauer A, Schulz PM, Klima A, Döpper S, Gruber G, Buchacher A, Muster T, Katinger H. Restricted antigenic variability of the epitope recognized by the neutralizing gp41 antibody 2F5. AIDS. 1996 Jun;10(6):587-93.
23. Conley AJ, Kessler JA 2nd, Boots LJ, Tung JS, Arnold BA, Keller PM, Shaw AR, Emini EA. Neutralization of divergent human immunodeficiency virus type 1 variants and primary isolates by IAM-41-2F5, an anti-gp41 human monoclonal antibody. Proc Natl Acad Sci U S A. 1994 Apr 12;91(8):3348-52.
24. Trkola A, Pomales AB, Yuan H, Korber B, Maddon PJ, Allaway GP, Katinger H, Barbas CF 3rd, Burton DR, Ho DD, et al. Cross-clade neutralization of primary isolates of human immunodeficiency virus type 1 by human monoclonal antibodies and tetrameric CD4-IgG. J Virol. 1995 Nov;69(11):6609-17.
25. Baba TW, Liska V, Hofmann-Lehmann R, Vlasak J, Xu W, Ayehunie S, Cavacini LA, Posner MR, Katinger H, Stiegler G, Bernacky BJ, Rizvi TA , Schmidt R, Hill LR, Keeling ME, Lu Y, Wright JE, Chou TC, Ruprecht RM. Human neutralizing monoclonal antibodies of the IgG1 subtype protect against mucosal simian-human immunodeficiency virus infection. Nat Med. 2000 Feb;6(2):200-6.
26. Binley JM, Wrin T, Korber B, Zwick MB, Wang M, Chappey C, Stiegler G, Kunert R, Zolla-Pazner S, Katinger H, Petropoulos CJ, Burton DR. Comprehensive cross-clade neutralization analysis of a panel of anti-human immunodeficiency virus type 1 monoclonal antibodies. J Virol. 2004 Dec;78(23):13232-52.
27. Mehandru S, Wrin T, Galovich J, Stiegler G, Vcelar B, Hurley A, Hogan C, Vasan S, Katinger H, Petropoulos CJ, Markowitz M. Neutralization profiles of newly transmitted human immunodeficiency virus type 1 by monoclonal antibodies 2G12, 2F5, and 4E10. J Virol. 2004 Dec;78(24):14039-42.
28. Armbruster C, Stiegler GM, Vcelar BA, Jäger W, Michael NL, Vetter N, Katinger HW. A phase I trial with two human monoclonal antibodies (hMAb 2F5, 2G12) against HIV-1. AIDS. 2002 Jan 25;16(2):227-33.
29. Armbruster C, Stiegler GM, Vcelar BA, Jäger W, Köller U, Jilch R, Ammann CG, Pruenster M, Stoiber H, Katinger HW. Passive immunization with the anti-HIV-1 human monoclonal antibody (hMAb) 4E10 and the hMAb combination 4E10/2F5/2G12. J Antimicrob Chemother. 2004 Nov;54(5):915-20. Epub 2004 Sep 29.
30. Mehandru S, Vcelar B, Wrin T, Stiegler G, Joos B, Mohri H, Boden D, Galovich J, Tenner-Racz K, Racz P, Carrington M, Petropoulos C, Katinger H, Markowitz M. Adjunctive Passive Immunotherapy in Human Immunodeficiency Virus Type 1-Infected Individuals Treated with Antiviral Therapy during Acute and Early Infection. J Virol. 2007 Oct;81(20):11016-31. Epub 2007 Aug 8.
31. Vcelar B, Stiegler G, Wolf HM, Muntean W, Leschnik B, Mehandru S, Markowitz M, Armbruster C, Kunert R, Eibl MM, Katinger H. Reassessment of autoreactivity of the

broadly neutralizing HIV antibodies 4E10 and 2F5 and retrospective analysis of clinical safety data. AIDS. 2007 Oct 18;21(16):2161-70.

32. Mascola JR, Lewis MG, Stiegler G, Harris D, VanCott TC, Hayes D, Louder MK, Brown CR, Sapan CV, Frankel SS, Lu Y, Robb ML, Katinger H, Birx DL. Protection of Macaques against pathogenic simian/human immunodeficiency virus 89.6PD by passive transfer of neutralizing antibodies. J Virol 1999 May;73(5):4009-18
33. Mascola JR, Louder MK, VanCott TC, Sapan CV, Lambert JS, Muenz LR, Bunow B, Birx DL, Robb ML. Potent and synergistic neutralization of human immunodeficiency virus (HIV) type 1 primary isolates by hyperimmune anti-HIV immunoglobulin combined with monoclonal antibodies 2F5 and 2G12.J Virol. 1997 Oct;71(10):7198-206. 7-73.
34. Hofmann-Lehmann R, Vlasak J, Chenine AL, Li PL, Baba TW, Montefiori DC, McClure HM, Anderson DC, Ruprecht RM. Molecular evolution of human immunodeficiency virus env in humans and monkeys: similar patterns occur during natural disease progression or rapid virus passage. J Virol. 2002 May;76(10):5278-84
35. Hofmann-Lehmann R, Vlasak J, Rasmussen RA, Jiang S, Li PL, Baba TW, Montefiori DC, Bernacky BJ, Rizvi TA, Schmidt R, Hill LR, Keeling ME, Katinger H, Stiegler G, Cavacini LA, Posner MR, Ruprecht RM. Postnatal pre- and postexposure passive immunization strategies: protection of neonatal macaques against oral simian-human immunodeficiency virus challenge. J Med Primatol 2002 Jun;31(3):109-19
36. Hofmann-Lehmann R, Vlasak J, Rasmussen RA, Smith BA, Baba TW, Liska V, Ferrantelli F, Montefiori DC, McClure HM, Anderson DC, Bernacky BJ, Rizvi TA, Schmidt R, Hill LR, Keeling ME, Katinger H, Stiegler G, Cavacini LA, Posner MR, Chou TC, Andersen J, Ruprecht RM. Postnatal passive immunization of neonatal macaques with a triple combination of human monoclonal antibodies against oral simian-human immunodeficiency virus challenge. J Virol 2001 Aug;75(16):7470-80
37. Ferrantelli F, Buckley KA, Rasmussen RA, Chalmers A, Wang T, Li PL, Williams AL, Hofmann-Lehmann R, Montefiori DC, Cavacini LA, Katinger H, Stiegler G, Anderson DC, McClure HM, Ruprecht RM. Time dependence of protective post-exposure prophylaxis with human monoclonal antibodies against pathogenic SHIV challenge in newborn macaques. Virology. 2007 Feb 5;358(1):69-78. Epub 2006 Sep 25.
38. Ferrantelli F, Hofmann-Lehmann R, Rasmussen RA, Wang T, Xu W, Li PL, Montefiori DC, Cavacini LA, Katinger H, Stiegler G, Anderson DC, McClure HM, Ruprecht RM.Post-exposure prophylaxis with human monoclonal antibodies prevented SHIV89.6P infection or disease in neonatal macaques. AIDS 2003 Feb 14;17(3):301-9
39. Ferrantelli F, Rasmussen RA, Buckley KA, Li PL, Wang T, Montefiori DC, Katinger H, Stiegler G, Anderson DC, McClure HM, Ruprecht RM.Complete protection of neonatal rhesus macaques against oral exposure to pathogenic simian-human immunodeficiency virus by human anti-HIV monoclonal antibodies.J Infect Dis. 2004 Jun 15;189(12):2167-73. Epub 2004 May 26.
40. Mauck, C., Z. Rosenberg, and L. Van Damme. 2001. Recommendations for the clinical development of topical microbicides: an update. AIDS 15:857-868.
41. Clarke, J. G., J. F. Peipert, S. L. Hillier, W. Heber, L. Boardman, T. R. Moench, and K. Mayer. 2002. Microflora changes with the use of a vaginal microbicide. Sex Transm.Dis. 29:288-293.
42. Hillier, S. L., M. A. Krohn, L. K. Rabe, S. J. Klebanoff, and D. A. Eschenbach. 1993. The normal vaginal flora, H2O2-producing lactobacilli, and bacterial vaginosis in pregnant women. Clin.Infect.Dis. 16 Suppl 4:S273-S281.
43. Nugent, R., M. Krohn, and S. Hillier. 1991. Reliability of diagnosing bacterial vaginosis is improved by a standardized method of gram stain interpretation. J Clin.Microbiol. 29:297-301.

44. Klebanoff, S. J. and R. W. Coombs. 1991. Viricidal effect of Lactobacillus acidophilus on human immunodeficiency virus type 1: possible role in heterosexual transmission. J Exp.Med. 174:289-292.
45. Martin, H. L., B. A. Richardson, P. M. Nyange, L. Lavreys, S. L. Hillier, B. Chohan, K. Mandaliya, J. O. Ndinya-Achola, J. Bwayo, and J. Kreiss. 1999. Vaginal lactobacilli, microbial flora, and risk of human immunodeficiency virus type 1 and sexually transmitted disease acquisition. J Infect.Dis. 180:1863-1868.
46. Paavonen, J. 1983. Physiology and ecology of the vagina. Scand.J Infect.Dis.Suppl 40:31-35.
47. Myer, L., L. Denny, R. Telerant, M. Souza, T. C. Wright, Jr., and L. Kuhn. 2005. Bacterial vaginosis and susceptibility to HIV infection in South African women: a nested case-control study. J Infect.Dis. 192:1372-1380.
48. Hugenholtz, P., B. M. Goebel, and N. R. Pace. 1998. Impact of culture-independent studies on the emerging phylogenetic view of bacterial diversity. J Bacteriol. 180:4765-4774.
49. Sha, B. E., H. Y. Chen, Q. J. Wang, M. R. Zariffard, M. H. Cohen, and G. T. Spear. 2005. Utility of Amsel criteria, Nugent score, and quantitative PCR for Gardnerella vaginalis, Mycoplasma hominis, and Lactobacillus spp. for diagnosis of bacterial vaginosis in human immunodeficiency virus-infected women. J.Clin.Microbiol. 43:4607-4612.
50. Verhelst, R., H. Verstraelen, G. Claeys, G. Verschraegen, J. Delanghe, L. Van Simaey, C. De Ganck, M. Temmerman, and M. Vaneechoutte. 2004. Cloning of 16S rRNA genes amplified from normal and disturbed vaginal microflora suggests a strong association between Atopobium vaginae, Gardnerella vaginalis and bacterial vaginosis. BMC.Microbiol. 4:16.
51. Zariffard, M. R., M. Saifuddin, B. E. Sha, and G. T. Spear. 2002. Detection of bacterial vaginosis-related organisms by real-time PCR for Lactobacilli, Gardnerella vaginalis and Mycoplasma hominis. FEMS Immunol.Med.Microbiol. 34:277-281.
52. Byun, R., M. A. Nadkarni, K. L. Chhour, F. E. Martin, N. A. Jacques, and N. Hunter. 2004. Quantitative analysis of diverse Lactobacillus species present in advanced dental caries. J Clin.Microbiol. 42:3128-3136.
53. Verhelst, R., H. Verstraelen, G. Claeys, G. Verschraegen, J. Delanghe, L. Van Simaey, C. De Ganck, M. Temmerman, and M. Vaneechoutte. 2004. Cloning of 16S rRNA genes amplified from normal and disturbed vaginal microflora suggests a strong association between Atopobium vaginae, Gardnerella vaginalis and bacterial vaginosis. BMC.Microbiol. 4:16.
54. Ritchie, J., and Spencer, L. (1994) Qualitative data analysis for applied policy research. In Bryman, A., and Burgess, R.G.(eds) (1994) Analyzing qualitative data. [London: Routledge]
55. Ulin, P., Robinson, E.T., and Tolley, E.E. (2005) Qualitative Methods in Public Health: A Field Guide for Applied Research [San Francisco: Jossey-Bass]

APPENDIX 1: BRIEF PARTICIPANT INFORMATION SHEET

BRIEF PARTICIPANT INFORMATION SHEET

MABGEL1: Clinical trial of a vaginal microbicide

This information sheet provides a brief description of the MABGEL1 clinical trial and what is involved. If, after reading this information sheet you would like to find out more, or you are interested in taking part in the study, please read the full participant information sheet. If you would like to discuss anything about the study, need a full information sheet, or you would like to take part, please contact:

David Thompson or Carol Taylor
01904 721893
mabgel@yorkhivresearch.org.uk

The full information leaflet is also available on our website:
www.yorkhivresearch.org.uk/volunteers

What is the purpose of this study?
Products called 'vaginal microbicides' are currently being developed as new ways to protect against Human Immunodeficiency Virus (HIV) and other sexually transmitted infections (STIs).

Vaginal microbicides can come in the form of pessaries or gels which a woman applies inside the vagina before sexual intercourse. The microbicide used in this clinical trial comes in the form of a gel. The main purpose of the study is to investigate what happens when a newly developed vaginal microbicide gel called 'Mabgel' is used every day for 12 days. We will do tests to see how long the active ingredient remains in the vagina and whether it is absorbed into the blood.

The active ingredient has not been used in the vagina in humans before. However, it has been used in previous tests where volunteers received an injection. In these tests the product was found to be safe, although some people experienced mild side effects (muscle pain after injection, joint pain or skin rashes). In this study, we will

collect information about any side effects people experience when they use the microbicide gel so that we can test whether the product is safe when used in the vagina. Some people will receive a gel with a high dose of the active ingredient, some will receive a low dose, and the rest will receive a placebo dose (a 'dummy' gel which does not contain the active ingredient).

Who can take part?
To take part in this study you must be a healthy female aged between 18 and 45 years old. More details about who can take part are provided on the full participant information leaflet and the research team will discuss these in full with you before you take part in the study.

What will happen if I take part?
Participation in the study will last approximately 6 to 12 weeks overall (this will depend on where you are in your menstrual cycle when you start the study). You need to be available for the whole of this time.

During this time, you will come to the clinical unit based in York for 8 visits. The first visit is a screening visit where we will check that you are happy to take part in the study and that you understand everything that you will need to do. We will also do some blood tests to check that you are healthy and you will have a vaginal examination.

At the next visit, we will do some more tests and we will give you the first dose of the gel. You will be given the other 11 doses of the study gel to take home and use on a daily basis. You will have another visit later on this day in the afternoon and then four more visits over the next 2 weeks. Your last study visit will take place a few weeks later to do some final tests.

For some of the visits, it is important that they are carried out at specific times of the day in relation to when you use the study gel. This means that for some of the visits you will need to be able to attend the clinical unit for a visit in the morning (for 1 to 1½ hours) and on one occasion you will also have to return for another visit on the same day in the afternoon. The research doctor or nurse will discuss this with you before you take part in the study.

Details about what will happen at each visit are provided on the full participant information leaflet and the research doctor or nurse will discuss this with you in full before you take part. In recognition of the time involved and inconvenience caused, you will receive financial reimbursement if you take part. The total amount you will receive if you complete the study in full is £565.

What should I do if I want to find out more?
It is important that you read the full information leaflet before taking part in the study. Please contact the research team using the details at the start of the information sheet to find out more, to get a copy of the full information sheet or to arrange a screening visit.

APPENDIX 2: PARTICIPANT INFORMATION SHEET

PARTICIPANT INFORMATION SHEET

MABGEL 1: A randomised double blind phase 1 study to assess the pharmacokinetics of C2F5, C2G12 & C4E10 when administered together in a gel vehicle as a vaginal microbicide

Clinical trial of a vaginal microbicide
Version 3.1, 08 Sep 2009
EudraCT: 2008-000312-32

You are being invited to take part in a research study. Before you decide, it is important for you to understand why the research is being done and what it will involve. Please ask the study doctor or nurse if there is anything that is not clear or if you would like more information. Take time to decide whether or not you wish to take part. Thank you for reading this leaflet.

Part 1 tells you the purpose of this study and what will happen to you if you take part. Part 2 gives you more detailed information about how the study will be carried out.

INFORMATION SHEET PART 1

What is the purpose of this study?

Throughout the world the most common way in which Human Immunodeficiency Virus (HIV) spreads is through sexual intercourse between men and women. Although condoms are very effective, it is not always possible for a woman to get her partner to agree to use them.

Therefore there is an urgent need for other methods of protection that can be used by women.

Products called 'vaginal microbicides' are currently being developed as new ways to prevent HIV and other sexually transmitted infections (STIs). Vaginal microbicides can come in the form of pessaries or gels which a woman applies inside the vagina before sexual intercourse. This means that women would be able to use them without necessarily having to negotiate this with their partners. The microbicide used in this clinical trial comes in the form of a gel.

The main purpose of this study is to investigate what happens when a newly developed vaginal microbicide gel is used every day for 12 days. From previous research that has been done using the active ingredient in this microbicide gel in humans, we expect the product to be very safe. In these previous tests participants received an injection, but the active ingredient has not been used in the vagina before. This study is the first time that the active ingredients have been applied vaginally. We will look at how long the active ingredient stays in the vagina and whether it is absorbed into the blood.

Participation in the study will last approximately 6 to 12 weeks overall.

Sub-studies

We will also investigate some other aspects of the vaginal microbicide gel and some features of the clinical trial itself (these can be referred to as sub studies):

1. We will look at how the study gel affects the normal balance of bacteria in the vagina;
2. We will investigate how vaginal samples collected in different ways (either self-collected or collected by a doctor) compare to one another; and
3. We will interview you about your experience of taking part in the trial.

Further details of these sub-studies can be found in part 2 of this information sheet.

Who can take part?

To take part in this study you must be:

- female
- between 18 and 45 years old
- in good health as determined by the screening tests carried out, and in the opinion of the study doctor
- willing and able to give written informed consent
- available for the duration of the study
- willing to be tested for HIV, hepatitis B & C, and sexually transmitted infections
- willing to abstain from vaginal practices from 48 hours before the first dose of the study gel until 24 hours after the last dose (15 days in total). This means that you cannot have sexual intercourse or receive oral sex during this time
- willing to use a reliable method of contraception for the duration of the study (although as described above you will not be allowed to have sex for some of this time). This must be either consistent use of condoms with every act of sexual intercourse, the combined oral contraceptive pill, Cerazette (desogestrel containing oral contraceptive pill), an intra-uterine contraceptive device or system, the injectable contraceptive, or a progesterone implant
- willing to abstain from using any genital preparations (such as creams or pessaries), other than the study gel, during the time when you are using the gel until 24 hours after the 12th dose
- willing to abstain from using tampons during the time when you are using the gel until 24 hours after the 12th dose
- willing and able to comply with the procedures required as described in this information leaflet and as directed by the study doctor or nurse
- have been registered with a GP for a minimum of 3 months
- have access to a fridge for the purposes of storing the study gel

You cannot take part in this study if:

- the above does not apply to you
- you have, or are found to have after the initial screening tests, untreated syphilis, gonorrhoea, trichomonas, chlamydia, thrush or bacterial vaginosis
- you have, or are found to have after the initial screening tests, HIV, hepatitis B or hepatitis C

- you have any abnormal findings/results from screening blood tests and pelvic examination
- you have a history of coagulation disorders
- you have any significant current general medical illness
- you have irregular menstrual bleeding which is likely to cause vaginal bleeding during the dosing period (as judged by the study doctor)
- you are currently taking part in another clinical study, or have taken part in another clinical study within the 2 months before the screening visit
- you have been treated for cervical intraepithelial neoplasia (CIN, these are abnormal cells on your cervix) or had any other surgical procedures affecting your cervix within the past 3 months
- you are pregnant, gave birth less than 12 weeks ago, or you are breast feeding
- have any condition which, in the opinion of the investigator, might interfere with evaluating the study objectives
- you are unable to read and speak fluent English

Do I have to take part?
It is up to you to decide whether or not to take part. If you do decide to take part you will be given this information sheet to keep and be asked to sign a consent form. If you decide to take part you are still free to withdraw at any time without giving a reason. A decision to withdraw at any time, or a decision not to take part, will not affect the standard of care you receive.

Table 1: Schedule of visits and tests over the course of the study

	Visit number								
	**1**	**2**		**3**	**4**	**5**	**6**	**7**	**8**
	**Screening visit**	**Before the 1st dose**	**1 hour after 1st dose**	**8 hours after the 1st dose**	**24 hours after the 1st dose**	**Mid-way through the dosing period**	**12 hours after the last dose**	**36 hours after the last dose**	**2-5 weeks after the last dose**
Questions about your general/sexual health	●								●
Physical examination	●								●
Gynaecological examination	●	●	●	●	●	●	●	●	●
Screen for sexually transmitted infections	●								
Blood tests to check your general health	●						●		●
**Types of samples we will take:**									
Blood samples*	●	●		●			●		●
Samples from your vagina	●	●	●	●	●		●	●	●

*We will take a maximum of 25mls of blood at each of the 5 study visits where blood samples are required (this is the equivalent to about 2 tablespoons of liquid).

What will happen to me if I take part?

The study will take place over approximately 6 to 12 weeks. Table 1 on the previous page shows the details of when your visits will take place and what will happen at each visit, if you decide to take part. All of the visits will take place at York Hospital.

Visit 1 (screening)

Your first visit will take place 2 to 6 weeks before the first dose of the study gel. At this screening visit you will have the opportunity to ask any questions and discuss the trial in full. If you decide you want to take part in the study you will then:

- sign the informed consent form
- be asked questions about your general health
- be asked questions about your sexual health, including questions about current and/or past sexual partners
- be asked permission for us to contact your General Practitioner (GP) for details of your medical history and to inform them that you are taking part in a clinical trial
- be given a full medical examination
- be given an internal vaginal examination using a plastic speculum (a hollow tube which is opened slightly so the doctor or nurse can see the cervix and vagina more easily). You will also have colposcopy done at this visit. Colposcopy is a more detailed examination of the cervix, where a magnifier (colposcope) is used to look at your cervix in more detail. A photograph of the cervix will be taken
- have vaginal, blood and urine samples taken for a number of routine laboratory tests to check it is safe for you to take part in the study. This includes tests for HIV, Hepatitis B and C infections, syphilis, gonorrhoea, chlamydia, trichomonas, bacterial vaginosis and thrush as well as a pregnancy test.

We will look at some provisional test results under the microscope within the research clinic: this will involve looking for thrush, bacterial vaginosis, trichomonas and gonorrhoea. We will be able to discuss these provisional results with you within 15-20 minutes of your examination. All the other test results can take up to 10 days to be processed. Once we have received your results we will contact you to talk about the next stage of the study. The next visit will take place 2 to 6 weeks after your initial screening visit.

If any of the tests or examinations detects an infection or another problem, there may be a need to stop your participation in the study. This is in order to ensure your safety. If we detect any problems we will discuss these with you and refer you to the appropriate person for treatment or care. Depending on the problem, this will be either a genitourinary medicine doctor or nurse or your GP. If the pregnancy test comes back positive you can be referred to a family planning professional if you wish. It may be possible to be screened again for entry into the study after treatment of some infections or problems. This will depend on the nature of the condition and will be at the discretion of the study doctor.

First application of the gel (2-6 weeks after your first visit)
On the first day of your next period after the screening visit, you will be asked to telephone the clinic to arrange your next appointment. This visit will take place between 7 and 13 days after the start of your period. At this visit you will:

- have an internal vaginal examination and colposcopy
- have vaginal and blood samples taken
- have cervico vaginal lavage. This is a way of taking samples of fluid from your vagina. A small volume of saline (salt water) is passed into your vagina, and then subsequently removed using a small plastic tube

The doctor or nurse will then administer the first dose of the study gel for you, using a vaginal applicator. You will also be given a demonstration of how to transfer the study gel into the applicator, so you can do this yourself at home for the remaining 11 doses.

The above examinations and tests will be repeated one hour after applying the gel. During this time you will be provided with refreshments and will be free to walk around.

During this visit we will give you:

- 11 vaginal applicators and 11 syringes containing the study gel for you to take home
- a spare plastic zipper bag to keep the used syringes and applicators in
- a leaflet to describe how to use the gel and how it should be stored
- a diary card to record when you use the study gel

You will return to the clinical unit later that day so that the tests can be repeated 8 hours after applying the gel.

You will then return to the clinical unit the next day so that the tests can be repeated 24 hours after applying the gel.

Application at home
For the following 11 days you will apply the study gel yourself at home. You will do this once a day, usually just before going to bed. If you have any problems or concerns about using the study gel at any time, please contact the research team using the contact details provided at the end of this leaflet.

Upon entering the trial, you will be given a card (similar to a credit card) with details of the trial and contact details in case of emergency. This should be carried at all times.

Storage of the study gels
We will provide the study gels in a plastic zipper bag. The study gels must be kept in the refrigerator at home and they must be kept in the bag to ensure they do not come into contact with food in your fridge. All used syringes should be kept in the separate plastic zipper bag provided, and returned to the research team at your next visit. You do not need to keep the used syringes in the fridge.

Follow up appointments
One of the research doctors or nurses will call you on two occasions to check whether you are having any problems with the gel. We will arrange a convenient time with you to do this.

During the time when you are using the gel, you will attend for another visit and have a vaginal examination.

On the day after your 5th dose, you will attend for another visit 12 hours after your last dose. The study doctor or nurse will arrange the time of this visit with you. We will collect vaginal, blood and urine samples from you, collect your diary card.

The next day you will attend for another visit 36 hours after your last dose of the study gel. At this visit you will have an internal vaginal

examination including colposcopy, and vaginal samples will be taken. You will also have cervico-vaginal lavage at this visit.

On the first day of your next period you will telephone the clinic to arrange your final appointment. This will take place between days 7 and 21 after the start of your period. At this visit you will be given a full medical examination. Vaginal swabs will be taken and you will have another pregnancy test. Blood samples will be taken to check that you are healthy.

Sometimes during the study it may be necessary to do extra tests to those described in this information sheet. This would only usually be if the study blood tests showed an abnormality that required further investigation. The study doctor will discuss these with you at the time if they are needed.

After the final visit we may need to contact you again in case extra investigations need to be done. Please do not book a holiday or leave the country for 2 weeks after your final visit.

Interview
At your final follow up visit, you will also be interviewed by a member of the research team. This interview will be carried out as a way to evaluate the trial, and to find out about your experience of taking part in the trial. At this interview you will be asked about what it was like for you to take part in the study, what parts of the trial you found easy or hard to carry out, your experiences of using the product and whether you would suggest any changes to how this study, or similar studies, should be run. The interview will take about 40 minutes, although it may be shorter or longer depending on the discussion.

Reimbursement
In recognition of the time involved and inconvenience caused, you will receive financial reimbursement if you take part. The total amount you will receive if you complete the study in full is £565. If you do not complete all of the study visits and procedures, you will receive a proportion of this amount. The amount will be dependent on how far through the course of the study you are before withdrawal. Payment will generally be provided at the end of the trial, in the form of a cheque.

What do I have to do?

To take part in the study you must make yourself available for all study visits and comply with the instructions given to you. You must also agree to abstain from sexual intercourse from 48 hours before the first dose of the study gel until 24 hours after the last dose (15 days in total). You must also use reliable contraception (defined as one of: consistent use of condoms with every act of sexual intercourse; combined oral contraceptive pill; intra-uterine contraceptive device; injectable contraceptive; or progesterone implant) from the first day of your period before the screening visit until the first day of your period after applying the final dose of the study gel.

What is the microbicide that is being tested?

The vaginal microbicide we are testing in this study is a gel which contains three monoclonal antibodies (proteins used by the body's immune system) which have been found to deactivate the HIV virus. We hope that the presence of these antibodies in the vagina during sexual intercourse will prevent infection with HIV. The study gel, however, is at the very early stage of development so this study will not collect information directly on how effective the study gel is at preventing HIV infection. Instead we will look at how long the active ingredient remains in the vagina and whether it is absorbed into the blood. This information will help us develop the microbicide gel in the future.

This is the first time that these antibodies have been administered intravaginally (put in the vagina) in humans, although they have been given to humans intravenously (injected into the blood) before. In all of these previous tests, the antibodies have been found to be safe in humans, with very few side effects. The gel comes in a syringe, which must be transferred into a vaginal applicator (a small plastic tube which is used for putting gel or cream into the vagina) before use. There is 2.5ml of gel in each dose.

This is a randomised study. This means that once you have agreed to enter the trial, you will receive either a) the gel with a low dose of the active ingredient, b) the gel with a higher dose of the active ingredient, or c) a placebo gel. The placebo is a 'dummy' gel, which looks and feels exactly like the active microbicide gel, but does not contain the active ingredient. We need to have a placebo group so that we can distinguish the effects of the active microbicide from those caused by the placebo gel.

The study is also 'double blinded'. This means that you will not know whether you have been given the active or placebo gel, and neither will the study doctor or nurse. The study is carried out in this way to avoid errors in the results.

What are the side-effects of any treatment received when taking part?

This is the first time that these antibodies and gel have been administered intravaginally in humans and the side effects are not yet known. The antibodies have been given to 39 people in previous clinical trials. In these studies the antibodies were injected into the blood. These participants experienced few side effects associated with the antibodies, and no one experienced any serious side effects. In two of the previous studies, about one third of participants experienced muscle pain after administration of the antibodies and a small number of people experienced joint pain or skin rashes. All side-effects were generally mild and resolved during or shortly after stopping using the antibodies.

The gel (either active or placebo) can cause vaginal discharge, vaginal spotting/bleeding, vulvo-vaginal itching or burning. These side effects are usually mild and resolve during or shortly after stopping using the gel.

We do not expect you to have any serious side effects from the antibodies or gel, but please tell us if you get any symptoms, whether or not you think they are caused by the study gel.

What are the possible disadvantages and risks of taking part?

Pregnancy:

It is possible that if the study gel is given to a pregnant woman it will harm the unborn child. Pregnant women must not therefore take part in this study, neither should women who plan to become pregnant during the study. Women who could become pregnant must use an effective contraceptive during the course of this study. You will have a pregnancy test before taking part to exclude the possibility of pregnancy. Any woman who finds that she has become pregnant while taking part in the study should immediately tell the study doctor or nurse.

Harm to sexual partners:

The safety of the study gel has not yet been tested. As this must be done in a controlled way, it is important that sexual partners do not

come into contact with the study gel. Therefore you must not have sexual intercourse or receive oral sex while you are using the study gel. Sexual intercourse and oral sex will also affect the tests we need to do for the study. Therefore you must not have sexual intercourse or receive oral sex from 48 hours before you use the study gel until 24 hours after the last dose.

Procedures:
Blood sampling can sometimes cause bruising and soreness of the arms, or very rarely a blockage of the vein or a small nerve injury which can cause numbness and pain. Normally these problems resolve with time. Some people may faint while blood is being drawn.

Vaginal examinations and having cervico-vaginal samples taken can feel uncomfortable for some women and will be very similar to having a smear test. These procedures may sometimes cause spotting.

If you insert the vaginal applicator too far it may cause a very small bruise or scratch to the cervix which you probably won't notice, but which may be seen during internal examination.

Some women may feel some embarrassment or discomfort from having to provide a detailed sexual history and from having internal vaginal examinations. All study procedures will be carried out by trained and experienced personnel, and we will take care to respect your dignity and privacy at all times.

What are the possible benefits of taking part?
We hope that people at risk of HIV infection will ultimately benefit from this study. This is especially relevant to people in developing countries, where HIV is a major problem.

As you are a healthy person, you will not gain any direct medical benefit from participating in this study. You will, however, receive a full medical examination. If, during the course of the study, we find that you have a medical condition of which you are unaware, we will inform you of our findings and refer you to your GP or an appropriate doctor.

What happens when the research study stops?
At the end of the trial, information will be held securely for a minimum of 15 years. You will be provided with contact details of the research

team in case you want to discuss any aspect of taking part in the trial at a later date.

What if there is a problem?
Any complaint about the way you have been dealt with during the study or any possible harm you might suffer will be addressed. The detailed information on this is given in Part 2.

Will my taking part in this study be kept confidential?
Yes. We will follow ethical and legal practices and all information about you will be handled in confidence. The details are included in Part 2.

This completes Part 1 of the information sheet. If the information in Part 1 has interested you and you are considering participation, please read the additional information in Part 2 before making any decision.

INFORMATION SHEET PART 2

What if new information becomes available?

Sometimes during the course of a trial, new information becomes available about the product being tested. If this happens, your study doctor will tell you about it and discuss with you whether you want to continue in the study. If you decide to withdraw, the study doctor will make arrangements for your care to continue. If you decide to continue in the study you may be asked to sign an updated consent form.

On receiving new information, the study doctor might consider it to be in your best interests to withdraw you from the study. He/she will explain the reasons and arrange for your care to continue.

What will happen if I don't want to carry on with the study?

You are free to withdraw from the study at any time, without giving a reason, and without your medical care or legal rights being affected. If you choose to stop using the study gel, you will be asked to continue to attend for follow up visits until the end of the trial, but you may choose not to do so. If you withdraw from the study completely the blood and tissue samples taken as part of the study so far will be stored and analysed, and the data you have provided in the study will be kept and stored, as described below.

What if there is a problem?

If you have a concern about any aspect of this study, you should ask to speak to the researchers who will do their best to answer your questions. If you remain unhappy and wish to complain formally, you can speak to a member of the PALS (Patient Advice and Liaison Service) team. The PALS team are based at York Hospital and can be contacted on York (01904) 726262. The PALS team are available Monday to Friday 8:30 am to 4:30 pm. Complaints can also be made in writing to:

Patient Experience Office
York Hospitals NHS Foundation Trust
Bootham Park Hospital
YORK
YO30 7BY

In the event that something does go wrong and you are harmed during the research you may have grounds to take legal action. York Hospitals

NHS Foundation Trust will have indemnity cover should you wish to pursue a claim, as will the University of York.

Will my taking part in this study be kept confidential?

Yes. Any personal information pertaining to your participation in the study will remain confidential. Access to this information is strictly controlled by authorised staff. All information which is collected about you during the course of the research will be kept strictly confidential, and any information about you which leaves the hospital or clinic will have your name and address removed so that you cannot be recognised. You will be assigned a unique study number which will be used to identify your information and biological samples that leave the hospital.

We will also enter your information onto a computer database. We will only use your unique study number, your initials and date of birth in this database (your name and address will not be included).

The interview will be tape-recorded and the information collected from this will be analysed and reported anonymously. We may use direct quotes from you in reports or published articles, but we will not use your name and you will not be identifiable from these quotes.

The information we collect from you during the course of the study will be processed for the purpose of the study, product registration purposes, and for ensuring compliance with medical, ethical, and pharmaceutical laws and regulations. Your information may be made available to regulatory authorities for the purpose of inspecting and validating our work, and it may be disclosed on a strict 'need to know' basis in case of medical emergencies.

Members of the research team, other authorised staff from York Hospitals NHS Foundation Trust, Covance (the company who are responsible for checking the study is being carried out properly and for entering the data into the study database), or regulatory authorities may need access to your study documents and medical records, including any medical records held at the sexual health clinic in York. By signing the consent form you will permit authorised staff participating in the research as collaborators or acting on behalf of regulatory authorities to review and use your study documents and/or medical records. Your personal information may be reviewed and copied (your identifying

personal data will not be copied) by such people during and after the study to verify clinical and scientific research procedures and/or data to the extent permitted by applicable laws and regulations and without breaching the confidentiality of the records. Even if you withdraw your consent, your personal data may still be processed so that we can verify our work.

Information we collect as part of this study, including your personal and medical details will be held by the research team, for a minimum of 15 years, in paper and electronic format for future reference.

Contacting your GP
We will ask you for your permission to contact your GP who will be informed that you are participating in a clinical trial. We will ask them to provide us with details of any relevant medical history that may prevent you from taking part in the trial, and confirm that you have not taken part in any other clinical trials in the past 2 months. You must therefore have been registered with a GP for at least 3 months so that they can give an informed opinion.

What will happen to my samples taken as part of the study?
The samples being taken will be treated as a 'gift', and you will not benefit financially if this research leads to the development of a new treatment or medical test. Samples will either be sent to York Hospital for analysis, or they will be stored at the University of York and later sent to either the Institute of Tropical Medicine in Antwerp or Polymun Scientific in Vienna for analysis. For those samples sent outside of the hospital, your name and address will be removed so that you cannot be recognised. After the samples have been analysed, they will be destroyed.

What are the sub-studies being carried out?
There are three sub-studies being conducted as part of this trial. Details of these are provided below:

1. *Investigation of how the study gel affects the normal balance of bacteria in the vagina*

There are many different types of bacteria which normally exist in a woman's vagina. These keep the vagina healthy, and a normal balance of these helps protect the body from infection. When the balance of these bacteria is changed, or when the pH (acid/alkaline balance) of

your vagina changes, this could potentially cause a condition called bacterial vaginosis. This is an unpleasant, but treatable condition, where women experience symptoms such as excess vaginal discharge or a discharge with a 'fishy odour'. A change in the pH or balance of bacteria may also affect how well the body can protect itself from sexually transmitted infections. Therefore it is important to know whether the use of the study gel affects the vagina in this way at all.

As part of the tests that we will do while you are in the study, we will collect a sample so we can look at the bacteria and pH in your vagina. We will also ask you about whether you have experienced any unusual discharge.

2. Investigation of how vaginal samples collected in different ways (either self-collected or clinician-collected) compare to one another

In this study, we do quite a few vaginal examinations to take samples of your normal vaginal fluid. We do this so that we can test what happens to the product when you have used it. In future trials, we might be able to reduce the number of vaginal examinations by asking women to take samples themselves. Before we can do this, however, we have to test whether we can use the self-collected samples in the same way as the samples taken by a nurse or a doctor.

During the course of the study, we will ask you to take three samples yourself using a small device shaped like a slim tampon applicator. We will give you instructions about how to use this device at the time of your clinic visit.

3. Interview about your experience of taking part in the trial

By exploring participants' experience of taking part in the study, we hope that we can improve the way we run studies for vaginal microbicides or similar products in the future. Therefore at your final follow up visit, you will be interviewed by a member of the research team. At this interview you will be asked about what it was like for you to take part in the clinical trial, what parts of the trial you found easy or difficult, your experiences of having examinations and using the product and whether you would suggest any changes to how similar studies should be run. The interview will take about 40 minutes, although it may be shorter or longer depending on the discussion.

This discussion will be tape-recorded and then transcribed. We will not use your name on the transcripts. The information will be analysed and reported anonymously. We may use direct quotes from you in reports or published articles, but we will not use your name and you will not be identifiable from these quotes. We will destroy the audio tapes after the analysis has been completed. We will store the transcripts of the interview for 5 years in a secure facility with access restricted to members of the research team. After this time the transcripts will be destroyed.

What will happen to the results of the research study?
The results of the study will be published in a peer-reviewed scientific journal. Results may also be presented at clinical conferences. You will not be identified in any publications or reports.

Who is organising and funding the research?
York Hospitals NHS Foundation Trust and the University of York are sponsoring the research. This means that they are responsible for the conduct of the study. The project is funded by the European Commission.

Who has reviewed the study?
All research in the NHS is looked at by an independent group of people, called a Research Ethics Committee to protect your safety, rights, wellbeing and dignity. This study has been reviewed and given favourable opinion by Cambridgeshire 1 Research Ethics Committee.

What should I do now?
If you have any questions about the study, or you think you would like to take part, please contact David Thompson or Carol Taylor in the research team on:

Tel: 01904 721893
Email: mabgel@yorkhivresearch.org.uk

APPENDIX 3: CONSENT FORM

York HIV Research Group

York Hospitals NHS
NHS Foundation Trust

MABGEL 1: A randomised double blind phase 1 study to assess the pharmacokinetics of C2F5, C2G12 & C4E10 when administered together in a gel vehicle as a vaginal microbicide

Clinical trial of a vaginal microbicide

08 Sept 2009, version 3.1
EudraCT: 2008-000312-32

**I acknowledge by my initials that:**		Participant initials
1.	I confirm that I have read and understand the information sheet dated 08 Sept 2009 (version 3.1) for the above study and have had the opportunity to ask questions.	
2.	I understand that my participation is voluntary and that I am free to withdraw at any time, without giving any reason and without my medical care or legal rights being affected.	
3.	I understand that sections of any of my medical notes and data collected during the course of the trial may be looked at by responsible individuals involved in the running or monitoring of the trial or from regulatory authorities where it is relevant to my taking part in research. I give permission for these individuals to have access to my records.	
4.	I understand that I will not benefit financially if this research leads to the development of a new treatment or medical test.	
5.	I understand that the samples being taken will be stored and analysed for the purposes of this study.	
6.	I agree to abstain from sexual intercourse from 48 hours before using the study gel until 24 hours after the last dose.	
7.	I agree to use an effective form of contraception for the duration of the study as stipulated in the information sheet.	
9.	I agree to my GP being contacted in order to inform them of my participation in this study, and I give permission for them to provide details about my relevant medical history.	
10.	I agree to take part in sub-study 1: 'Investigation of how the study gel affects the normal balance of bacteria and microbes in the vagina'	
11.	I agree to take part in sub-study 2: 'Investigation of how vaginal samples collected in different ways (either self-collected or clinician-collected) compare to one another'	
12.	I agree to take part in sub-study 3: 'Interview about your experience of taking part in the trial'. I understand that this will be audio-recorded, that direct quotations may be used in reports, and that I will not be recognisable from any of these reports.	
13.	I agree to take part in the above study and to abide by the restrictions set out in the information sheet.	

____________________	___/___/___	____________________
Name of Participant	Date	Signature
____________________	___/___/___	____________________
Name of Person taking consent	Date	Signature

3 copies: 1 for participant, 1 for trial master file, 1 to be kept with medical records

APPENDIX 4: GP LETTER

York HIV Research Group

York Hospitals NHS
NHS Foundation Trust

Mabgel1 Clinical Trial Team
Experimental Medicine Unit
Learning and Research Centre
York Hospital
Wigginton Road
York, YO31 8HE
Tel: 01904 721893
Email: mabgel@yorkhivresearch.org.uk

Doctor Address

Date

Dear Dr ____________

Re: MABGEL1: A randomised double blind phase I study to assess the pharmacokinetics of C2F5, C2G12 & C4E10 when administered together in a gel vehicle as a vaginal microbicide (EudraCT number: 2008-000312-32)

We are writing to you about <<participant name>>, (<<date of birth: dd/mmm/yyyy>>), who we understand is your patient, and who has consented to be entered into the above trial. This is a study to assess the pharmacokinetics of a microbicide gel for the prevention of HIV, to be administered intra-vaginally on a daily basis for 12 consecutive days.

Please find enclosed a participant information sheet and the eligibility criteria for this trial.

In order for us to proceed with enrolling your patient into this study, we are seeking confirmation from you, her GP, that a) she has not recently participated in any other clinical trials, b) she has no significant ongoing illness and c) there is no reason that you feel your patient should not be entered into the study. We therefore enclose a very short questionnaire for you to complete in order to confirm these details. We also enclose a copy of the signed consent form, which indicates that the participant has provided consent for us to contact you to request this information.

I would be grateful if you could complete this form as soon as feasible and return it in the enclosed prepaid envelope. Upon receipt of the completed form we will send you an honorarium of £40 in the form of a cheque.

You will be kept up to date with your patient's progress but if you have any concerns or questions in the meantime, please do not hesitate to contact me.

Yours sincerely,

Prof Charles JN Lacey
Chief Investigator

<u>Eligibility Criteria:</u>

Inclusion criteria

- Females
- Aged ≥18 and ≤45 years
- In good health as determined by medical history, physical examination and clinical judgement
- Willing and able to give written informed consent
- Available for the duration of the study
- Willing to undergo screening for HIV, Hepatitis B & C, and sexually transmitted infections
- Willing to abstain from vaginal practices including receptive oral sex and sexual intercourse from 48 hours before the first dose of the study gel until after visit 7
- If physiologically fertile, using a reliable method of contraception for the 3 to 4 menstrual cycles covering the pre-study, study dosing and follow up cycle to visit 8 (methods defined as one of: consistent use of condoms with every act of sexual intercourse; combined oral contraceptive pill; desogestrel-containing progesterone only pill (Cerazette); intra-uterine contraceptive device or system; injectable contraceptive; progesterone implant)
- Willing to abstain from using any genital preparations, other than the study gel, during the period of gel administration until after visit 7
- Willing to abstain from using tampons during the period of gel administration until after visit 7
- Judged by clinician to be able, and likely, to comply with the procedures required as set out in the protocol
- Have been registered with a GP for at least the past 3 months
- Response received from GP before randomisation
- Have access to a domestic refrigerator at home for the purposes of storing the study gel

Exclusion criteria

- Untreated syphilis, gonorrhoea, trichomonas, chlamydia, vaginal candidosis or bacterial vaginosis
- Clinically significant (out of the normal range and deemed clinically significant by the CI or study physician) haematological, biochemical, immunological or coagulation assay abnormalities on screening
- HIV infection, anti-HCV antibody positive, HbsAg positive
- Abnormal findings on pelvic examination, deemed clinically significant by the CI or study physician
- History of coagulation or thrombotic disorders
- Significant current general medical illness
- Irregular menstrual bleeding likely to cause vaginal bleeding during the dosing period as judged by the CI or study physician
- Current participation, or participation within the last 2 months in another clinical trial
- Treatment for cervical intraepithelial neoplasia (CIN) or other gynaecological instrumentation of the cervix within the past 3 months
- Pregnant, within 12 weeks postpartum, or breast feeding
- Unlikely to comply with protocol
- Have any condition which, in the opinion of the CI or study physician, might interfere with the evaluation of the study objectives

Unable to fluently read and speak English to a level adequate for the full comprehension of procedures required in participation and consent

Confirmation of eligibility for participation in 'Mabgel1' clinical trial

PATIENT IDENTIFICATION

Surname: ______________________

First Name: ______________________

Date of Birth: ☐ ☐ ☐

DAY MONTH YEAR

PATIENT HISTORY

Is this patient currently registered with your practice?	Yes ☐	No ☐
Has this patient been registered with you for at least 3 months?	Yes ☐	No ☐
If 'no' please indicate the date of registration with your practice:	☐ ☐ ☐<br>DAY MONTH YEAR	

If you answer 'yes' to any of the following questions, please provide details in the 'comments' section below.

Has the patient been involved in another clinical research study within the past 2 months?	Yes ☐	No ☐
Does the patient have any history of coagulation disorders, such as vitamin K deficiency, von Willebrand's disease, haemophilia or any clotting factor deficiencies?	Yes ☐	No ☐
Does the patient have a history of any significant general medical illness?	Yes ☐	No ☐
Has the patient received treatment for cervico-epithelial neoplasia (CIN) or other gynaecological instrumentation of the cervix within the past 3 months?	Yes ☐	No ☐
Has the patient receiving any prescription medication from your practice in the last 12 months ?	Yes ☐	No ☐
Is there any reason that you feel your patient should not be entered into this study?	Yes ☐	No ☐

Please turn over

COMMENTS

Please add any relevant comments below. Please continue on another sheet if necessary.

I hereby declare that the information provided is accurate and up to date, to the best of my knowledge.

DAY	MONTH	YEAR

Signature Name (Printed) Date

Please return this form in the prepaid envelope provided to:

Mabgel1 Clinical Trial Team
Experimental Medicine Unit
Learning and Research Centre
York Hospital
Wigginton Road
York, YO31 8HE

Thank you.

APPENDIX 5: MABGEL – STORAGE INSTRUCTIONS AND INSTRUCTIONS FOR USE

York HIV Research Group

York Hospitals NHS
NHS Foundation Trust

Mabgel – Instructions for use

How should I store the gel?

You have been provided with 11 doses of the study gel in individually packed syringes. If you think you have not been given the correct number of doses, please contact the research team immediately, using the contact details below.

As soon as you get home, please place the syringes containing the study gel in the fridge. Keep the syringes inside the plastic bag provided. Keep each dose of the study gel in the fridge and in its original packaging until you are ready to use it.

Please keep all used syringes in the extra bag provided. Do not keep this bag in the fridge.

When should I use the gel?

Dose 1 will have been applied by a nurse or doctor at the clinical unit.

Doses 2 to 11 should be applied at home, just before going to bed.

Dose 12 should be applied at home in the evening at <<enter time>>, as instructed by the study doctor or nurse.

How to use the gel:

1. Wash your hands before using the study gel.
2. Remove the syringe from its outer packaging (all of the syringes are the same, so it doesn't matter which one you use first).
3. Remove the rubber stopper from the end of the syringe.
4. Place the end of the syringe into the end of the applicator and hold in place. Check that the plunger on the applicator is fully pressed down.
5. Gently push down the plunger on the syringe, bringing the gel into the cylinder of the applicator. Make sure that the plunger on the syringe is fully pressed down so that all of the gel has been transferred into the applicator.
6. Remove the syringe from the applicator.
7. While lying down, knees bent and spread apart, gently insert the cylinder of the applicator well into the vagina. Press the plunger to transfer the gel from the applicator to the vagina. Make sure that the plunger is fully pressed down before removing.
8. With the plunger still depressed, remove the applicator, holding it by the cylinder.
9. Dispose of the used applicator in the bin, but do not throw away the used syringe.
10. Place the used syringe and rubber stopper into the plastic bag provided. Return all used syringes to the clinical trial unit when you come back for your follow up appointment.
11. Wash your hands.
12. Remember to record the time that you used the gel in the diary card.

If you have any problems using the gel or you have any other questions or concerns, contact the study team on 01904 721893 at any time.

APPENDIX 6: GRADING OF GENITAL REACTIONS

PARAMETER	Grade 1<br>Mild	Grade 2<br>Moderate	Grade 3<br>Severe	Grade 4<br>Life-threatening
**Genital discomfort (including dysuria, pruritis, dyspareunia)**	Easily tolerated	Aware of symptoms at most times<br>But not preventing daily activities. Able to control symptoms with medication from pharmacies	Unable to control symptoms such that cannot undertake essential daily activities	Hospitalisation for pain control
**Non-menstrual bleeding**	Light (spotting or brown discharge) ≤ 7 days	Light (spotting or brown discharge) > 7 days<br>Or<br>Heavy (bleeding like menses)<br>≤ 4 days	Heavy (bleeding like menses)<br>> 4 days<br>Or<br>Profuse of any duration	Hospitalisation for transfusion
**Signs of epithelial disruption (sores or ulcers)**	≤1 swab-tip*	>1 ≤ 4 swab-tips*	> 4 swab-tips*	Hospitalisation
**Erythema (redness)**	Local/diffuse mild redness<br>or<br>Local moderate/severe redness	Diffuse moderate/severe redness<br>With genital discomfort symptoms that are easily tolerated	Diffuse moderate/severe redness with Grade 2 genital discomfort symptoms	Hospitalisation
**Oedema**		Oedema without sloughing of the epithelial cells	Oedema with sloughing of the epithelial cells	

***swab-tip is 5 x 10mm**

APPENDIX 7: GRADING OF CLINICAL AND LABORATORY ADVERSE EVENTS

Based on systems in use at the MRC CTU and NIH Division of AIDS (unless stated otherwise).

Abbreviations:

ULN	Upper Limit of Normal	LLN	Lower Limit of Normal
R_x	Therapy	Req	Required
Mod	Moderate	IV	Intravenous
ADL	Activities of Daily Living	Dec	Decreased
OTC	Over the counter		

The tables below define clinically significant abnormal pathology results which are classed as AEs. Any results that are outside of the normal range for the laboratory at York Hospital but do not meet the criteria for being classed as an AE will not be classed as such. For events or results that are not specified in the tables, a decision will be made by the CI or a trial physician, as delegated by the CI, as to whether the result is clinically significant. Only those events or results that are deemed clinically significant will be classed as AEs.

For clinical events not specified in the tables below the following grading should be applied:

- *Grade 1 (mild):* Awareness of sign or symptom but tolerated with minimal or no interference with ADLs.Transient (< 48 hours). May be relieved by OTC medication but no other medical intervention required.
- *Grade 2 (moderate):* Notable symptoms resulting in greater than minimal interference with ADLs. No or minimal medical intervention required.
- *Grade 3 (severe):* symptoms causing significant incapacity resulting in bed rest or activity reduced by >50% of usual level and/or unable to work. Medical intervention required.
- *Grade 4 (extreme/life-threatening):* Significant medical intervention/therapy required; hospitalization required to prevent permanent impairment or death.

LABORATORY PARAMETERS

PARAMETER	GRADE 1 MILD	GRADE 2 MODERATE	GRADE 3 SEVERE	GRADE 4 EXTREME
**HAEMATOLOGY**				
Hb	95-105g/L	80-94g/L	79-65g/L	<65g/L
White Blood Count<br>Upper<br>Lower	 >13.0<br><3.5	 >15.0<br><3.0	 >20.0<br><2.0	 >30.0<br><1.0
Absolute Neutrophils	1-1.5 x 10^9/L	0.75-0.999 x 10^9/L	0.500-0.749 x 10^9/L	<0.500 x 10^9/L
Platelets	75.0-120.0/mm^3	50.0-74.99/mm^3	20.0-49.99/mm^3	<20.0/mm^3
PT	>1- 1.25 x ULN	>1.25- 1.5 x ULN	> 1.5- 3.0 x ULN	> 3.0 x ULN
APTT	>1-1.66 x ULN	>1.66- 2.33 x ULN	> 2.33- 3.0 x ULN	> 3.0 x ULN
**BIOCHEMISTRY**				
Potassium<br>Hyperkalemia<br>Hypokalemia	 5.0 – 5.5 meq/L*<br>3.2 – 3.4 meq/L	 5.6 – 6.0 meq/L<br>3.0 – 3.1 meq/L	 6.1 – 6.5 meq/L<br>2.5 – 2.9 meq/L	 >6.5 meq/L<br><2.5 meq/L
Sodium<br>Hypernatraemia<br>Hyponatraemia	 146 - 150 meq/L<br>130 – 135 meq/L	 151 - 157 meq/L<br>123 - 129 meq/L	 158 - 165 meq/L<br>116 - 122 meq	 >165 meq/L<br><116 meq/L
Bilirubin<br>Hyperbilirubinemia	 >1.25 – 2.0 x ULN	 >2.0 – 2.5 x ULN	 >2.5 – 5 x ULN	 >5 x ULN
Transaminases<br>AST (SGOT)<br>ALT (SGPT)<br>GGT<br>Alk Phos	 1.25 – 2.5 x ULN<br>1.25 – 2.5 x ULN<br>1.25 – 2.5 x ULN<br>1.25 – 2.5 x ULN	 >2.5 – 5.0 x ULN<br>>2.5 – 5.0 x ULN<br>>2.5 – 5.0 x ULN<br>>2.5 – 5.0 x ULN	 >5.0 – 10.0 x ULN<br>>5.0 – 10.0 x ULN<br>>5.0 – 10.0 x ULN<br>>5.0 – 10.0 x ULN	 > 10.0 x ULN<br>> 10.0 x ULN<br>> 10.0 x ULN<br>> 10.0 x ULN
Creatinine	>1.0 – 1.5 x ULN	>1.5 – 3.0 x ULN	>3.0 – 6.0 x ULN	>6.0 x ULN
Haematuria	Microscopic only $\leq$10 RBC/HPF	>10 RBC/HPF	Gross, with or without clots OR RBC casts	Obstructive OR transfusion req

Note: For Na^+ or K^+ 1 meq = 1 mmol

IMMUNOLOGY

C Reactive Protein	15 - 50mg/L	>50 – 150 mg/L	>150 – 250 mg/L	> 250 mg/L
Anti-cardiolipin antibodies IgG	> 1 - 1.25 x ULN	> 1.25- 1.5 x ULN	>1.5 – 3.0 x ULN	> 3.0 X ULN

Note: The above AE criteria for CRP and anti-cardiolipin antibodies have been devised by the CI as no DAIDS/MRC CTU gradings exist for these parameters.
In contrast to IgG antiphospholipid antibodies, elevated levels of IgM anticardiolipin antibodies are significantly less indicative of possible antiphospholipid syndrome/SLE and have not been induced by C4E10, C2F5 or C2G12 in previous studies. They will therefore not considered an adverse event unless accompanied by raised IgG, coagulation parameter abnormalities or clinical features suggestive of antiphospholipid syndrome/SLE.

CLINICAL PARAMETERS

PARAMETER	GRADE 1 MILD	GRADE 2 MODERATE	GRADE 3 SEVERE	GRADE 4 EXTREME
**CARDIOVASCULAR**				
Cardiac Arrhythmia		Asymptomatic; transient dysrhythmia, no R_x req	Recurrent/persistent dysrhythmia; symptomatic R_x req	Unstable dysrhythmia, hospitalisation and R_x req
Hypertension	Transient, increase >20 mm Hg diastolic BP; no $R_x$req	Recurrent; chronic increase >20 mm Hg diastolic BP; R_x req	Acute R_x req; outpatient	Hospitalisation req OR end organ damage
Hypotension	Transient orthostatic hypotension with heart rate increased by >20 beats/min OR decreased by > 10 mm Hg systolic BP, no R_x req	Symptoms OR BP decreased by >20 mm Hg systolic, correctable with oral fluid R_x	IV fluid req	Mean arterial pressure <60 mm Hg, OR end organ damage, OR shock, vasopressor R_x req OR hospitalisation
Pericarditis	Minimal effusion	Mild/mod asymptomatic effusion, no R_x	Symptomatic effusion, pain, EKG changes	Tamponade OR pericardiocentesis OR surgery req
Haemorrhage, blood loss		Mildly symptomatic, no R_x req	Gross blood loss OR 1-2 units transfused	Massive blood loss OR >2 units transfused
**GASTROINTESTINAL**				
Diarrhoea	Mild OR transient; 3-4 loose stools per day OR mild diarrhoea lasting <1 week	Mod OR persistent; 5-10 loose stools per day OR diarrhoea lasting $\geq$1 week	>10 loose stools/day bloody diarrhoea; OR orthostatic hypotension OR electrolyte imbalance, >2 L IV fluid req	Hypotensive shock OR severe electrolyte imbalance
Oral Discomfort/ Dysphagia	Mild discomfort, no difficulty swallowing	Difficulty swallowing but able to eat and drink	Unable to swallow solids	Unable to drink fluids; IV fluids req
Constipation	------------	Moderate abdominal pain 78 hours with impaction require outpatient prescription	Requiring disimpaction or hospital treatment	Distention with vomiting OR obstipation
**PULMONARY**				
Bronchospasm Acute	Transient; no R_x; FEV1 or peak flow reduced to 70% - 80%	R_x req; normalizes with bronchodilator; FEV1 or peak flow 50% - 69%	No normalization with bronchodilator; FEV1 or peak flow 25% - 49%, retractions	Cyanosis; FEV1 or peak flow <25% OR intubated
Dyspnoea	Dyspnoea on exertion	Dyspnoea with normal activity	Dyspnoea at rest	Dyspnoea requiring O_2

				therapy
**NEUROLOGICAL**				
Neuro-cerebellar	Slight incoordination OR Dysdiadochokinesia	Intention tremor OR dysmetria OR slurred speech OR nystagmus	Ataxia requiring assistance to walk or arm incoordination interfering with ADLs	Unable to stand
Neuro-psych/mood	---------------	---------------	Severe mood changes requiring medical intervention; suicidal ideation	Acute psychosis req hospitalisation ; suicidal gesture/attempt
Parasthesia (burning, tingling, etc.)	Mild discomfort; no R_x req	Mod discomfort; non-narcotic analgesia required	Severe discomfort; OR narcotic analgesia req with symptomatic improvement	Incapacitating; OR not responsive to narcotic analgesia

Neuro-motor	Mild weakness in muscle of feet but able to walk and/or mild increase or decrease in reflexes	Mod weakness in feet (unable to walk on heels and/or toes), mild weakness in hands, still able to do most hand tasks and/or loss of previously present reflex or development of hyperreflexia and/or unable to do deep knee bends due to weakness	Marked distal weakness (unable to dorsiflex toes or foot drop, and mod proximal weakness e.g., in hands interfering with ADLs and/or requiring assistance to walk and/or unable to rise from chair unassisted	Confined to bed or wheel chair because of muscle weakness
Neuro-sensory	Mild impairment (decreased sensation, e.g., vibratory, pinprick, hot/cold in great toes) in focal area or symmetrical distribution	Mod impairment (mod decreased sensation, e.g., vibratory, pinprick, hot/cold to ankles) and/or joint position or mild impairment that is not symmetrical	Severe impairment (decreased or loss of sensation to knees or wrists) or loss of sensation of at least mod degree in multiple different body sites (i.e., upper + lower extremities)	Sensory loss involves limbs and trunk
Eye		Mild pain, visual changes, conjunctivae erythema, abnormal slit lamp	Loss of vision, clinically diagnosed uveitis, mod-severe pain, glaucoma	---------------
Headache	Mild, no R_x req, OR over the counter medication	Mod; OR requiring regular OTC or occasional prescription only medication	Severe; intractable; OR requiring repeated prescription only medication	Requiring hospitalisation, or associated with neurological, respiratory or cardiovascular abnormalities

**MUSCULOSKELETAL**				
Arthralgia/ Arthritis	Arthralgia	Arthralgia with joint effusion or moderate impairment of activity	Frank arthritis with or without effusion OR resulting in severe impairment of activity	Hospitalisation
Myalgia	Myalgia without limitation of activity	Muscle tenderness at other than injection site or with moderate impairment of activity eg difficulty climbing stairs	Frank myonecrosis OR with severe impairment of activity eg can't climb stairs	Hospitalisation
**DERMATOLOGICAL**				
Rash*	Erythema, pruritis	Diffuse maculopapular rash or dry desquamation	Vesiculation or moist desquamation or ulceration	Any 1 of: suspected Steven's Johnson's Syndrome, erythema Multiforme, exfoliative dermatitis
GENERAL				
Fever Oral>12 hours	37.7 - 38.9°C (100.0 – 101.5°F)	39.0 – 39.7°C (101.6 – 102.9°F) OR max temp of 103°F	39.8 – 40.5°C (103 - 105°F) OR max temp of 103.5°F	>40.5°C (105°F) OR max temp of >105°F
Systemic Allergic Reaction*	Pruritis without rash or urticaria	Localised urticaria	Generalised urticaria or angioedema	Anaphylaxis

* For local reaction see section on genital reactions (appendix 6)

APPENDIX 8: SOURCE DATA DEFINITION

Type of Data	Source Document
Informed consent	Paper copy in TMF
Relevant Medical History and Current Medical Conditions	Proforma* and letter from GP where available
Physical Examination	Proforma
Concurrent medication	Proforma
Clinical Trial History	Proforma and letter from GP where available
Fulfilment of eligibility criteria	Proforma
Demographics	Proforma
Clinical Laboratory Reports – Haematology, Biochemistry, HIV, Syphilis, CT, GC	Printed report form, kept in participant's file
Urinalysis, Urine pregnancy tests Screening for vaginal candidosis, BV and TV	Proforma
Colposcopy	Proforma and digital colposcopy image
Time of study gel administration in the clinical unit	Proforma
Time of study gel administration at home	Diary card
Date/time of biological sampling	Proforma
Date of visits/examinations	Proforma
Drug accountability	IMP log and used syringes
Adverse events	Proforma
Protocol Deviations	Proforma
Withdrawal	Proforma
Pharmacokinetic parameters	Report in TMF
Vaginal flora assessment	Report in TMF

* Proforma used in participant's file.
