# Supplementary material for: MABGEL 1: First Phase 1 Trial of the Anti-HIV-1 Monoclonal Antibodies 2F5, 4E10 and 2G12 as a Vaginal Microbicide
Source: PLoS One. 2014 Dec 29;9(12):e116153. doi: 10.1371/journal.pone.0116153 (PMC4278856; doi:10.1371/journal.pone.0116153)
Supplement: S2 Protocol — Final Approved Protocol for In vitro stability study of 2F5, 4E10, 2G12 in vaginal secretions. (PDF) [file pone.0116153.s003.pdf]

Title of Study	Short Time Stability of the Monoclonal Antibodies C2F5, C2G12 and C4E10 in Vaginal Secretion
Study Code	PY/MAB/2011/01
Protocol Version	Version 1, 18.10.2011
Study Sites	ITM, Antwerp, Belgium<br>Polymun GmbH, Klosterneuburg, Austria
Principal Investigator	Dr. Vicky Jespers

Protocol Approval	Date	Signature
Dr Vicky Jespers<br>Prince Leopold Institute of Tropical Medicine (ITM), Antwerp, Belgium		
Dr. Brigitta Vcelar<br>Polymun Scientific GmbH, Klosterneuburg, Austria	18.10.2011	Brigitta Vcelar

The information contained herein is the property of Polymun Scientific Immunbiologische Forschung GmbH and CONFIDENTIAL. The recipient is responsible for safe-keeping and prevention of unauthorised appropriation, use or disclosure.

**Title of Study**	Short Time Stability of the Monoclonal Antibodies C2F5, C2G12 and C4E10 in Vaginal Secretion
**Study Code**	PY/MAB/2011/01
**Background/Rationale**	It has been proposed to use anti-HIV antibodies as microbicides, to protect against vaginal infection with HIV. Protective effects were demonstrated in non human primates after vaginal challenge with SHIV.<br>Vaginal secretion is part of the natural mucosal barrier function and includes proteases and low pH. Stability of passively administered IgG antibodies in this environment has not been investigated.<br>After single application of Mabgel in human PK study antibodies were detectable for 24 hours. Rapid decay was observed within the first hours following application. It is unclear if this rapid reduction can be attributed to the wash out of the applied gel or if enzymatic or microbial destruction is involved. This study aims to investigate the stability of the three broadly neutralizing HIV antibodies C2F5, C2G12 and C4E10 in vaginal secretion obtained from healthy females. Stability of antibodies will be assessed by binding ELISA specific for each antibody. Only complete IgG molecules with give a positive signal as the ELISA requires binding to the epitope as well as to the Fc part.<br>Test of neutralizing activity will not be performed with the samples, since no preservatives will be added prior to incubation at 37°C and a high bioburden would impair the neutralization assay.
**Study Sites**	ITM, Antwerp, Belgium<br><br>Polymun GmbH, Klosterneuburg, Austria
**Principal Investigator**	Vicky Jespers
**Study Duration**	2 days
**Primary Objective**	To investigate the short term stability of the monoclonal antibodies C2F5, C2G12 and C4E10 in vaginal secretion.
**Study Design**	This is an *in-vitro* stability study of the mAb C2F5, C2G12 and C4E10 in freshly collected vaginal secretion.<br>1 ml of vaginal secretion (pool of samples) will be mixed with 1 ml antibody preparation (mix of 3 antibodies containing 10 mg C2F5, 10 mg C2G12 and 10 mg C4E10 / ml). Samples will be vortexed shortly and incubated at 37 °C.<br><br>100 µl samples will be taken at the following times:<br>– before incubation (2 x 100 µl) and after

The information contained herein is the property of Polymun Scientific Immunbiologische Forschung GmbH and CONFIDENTIAL. The recipient is responsible for safe-keeping and prevention of unauthorised appropriation, use or disclosure.

	– 15 min,<br>– 30 min,<br>– 60 min<br>– 120 min<br>– 240 min and<br>– 480 min and<br>– 24 h of incubation.<br><br>100 µl samples will be diluted with 900 µl buffer, vortexed and frozen immediately at -70 °C.<br><br>Spiking and incubation of samples will be performed at the ITM. Frozen samples will be shipped on try ice to Polymun Scientific GmbH, were antibody content will be analysed by specific binding ELISA.
**Vaginal Samples**	Samples will be obtained from healthy volunteers participating in the clinical study Mucosal Immunology Samples Protocol, which was approved by the IRB of the Institute of Tropical Medicine Antwerp.<br>1 ml pure vaginal secretion, collected within 2 h before study initiation. To obtain a volume of 1 ml it will be necessary to pool samples of 2-3 subjects.
**Antibodies**	Antibody mix containing 10 mg/ml C2F5,<br>10 mg/ml C2G12 and<br>10 mg/ml C4E10<br>Excipient: 2 mM acetic acid, 10 % maltose, pH 4
**Other Materials**	Dilution buffer: PBS pH 7,2 – 7,4 + 1% BSA (according Polymun SOP#AI0027)<br>Calibrated pipettes 100 µl and 1000 µl<br>Centrifuge tubes, 10 ml, bottom shape: conical<br>37° C Incubator<br>Sarstedt vials: 1.5 ml<br>- 70° C freezer
**Study conduct**	1. Pure vaginal secretion is collected from volunteers participating in the "Mucosal Immunology Samples Protocol"<br>2. Fresh samples can be stored on ice for not longer than 2 hours.<br>3. Vaginal secretion is pooled to obtain a minimum of 1 ml. Patient numbers, collection time and date, approximate sample volume and pool number are recorded. After short vortexing 1 ml of the pool is transferred into a fresh centrifugation tube with screw cap.<br>4. 1 ml antibody mix (same amount as pool of secretion) is added and vortexed shortly.<br>5. 2 x 100 µl are pipetted to separate cryotubes for baseline evaluation (time 0 h). - See instructions for sample taking

The information contained herein is the property of Polymun Scientific Immunbiologische Forschung GmbH and CONFIDENTIAL. The recipient is responsible for safe-keeping and prevention of unauthorised appropriation, use or disclosure.

	below.<br>6. Incubate remaining mix at 37° C. Tube has to be closed tightly to avoid evaporation.<br>7. Further sampling times: 15 min, 30 min, 1 h, 2 h, 4 h, 8 h and 24 h (see study schedule on page 5).<br><br>Procedure for sample taking:<br>1. Label cryotube appropriately with pool number and incubation time<br>2. Remove centrifuge tube from incubator and vortex shortly<br>3. Transfer 100 µl sample into the cryotube<br>4. Add 900 µl dilution buffer<br>5. Vortex again<br>6. Freeze immediately at -70°C
**Methods of Analysis:**	Samples will be analysed by binding ELISA specific for each antibody. It is hypothesised that only intact antibody will be detected.<br>C2F5 Polymun SOP # AI0020<br>C2G12 Polymun SOP # AI0005<br>C4E10 Polymun SOP #AI0025

The information contained herein is the property of Polymun Scientific Immunbiologische Forschung GmbH and CONFIDENTIAL. The recipient is responsible for safe-keeping and prevention of unauthorised appropriation, use or disclosure.

Study Schedule				
Sample	Sampling times	Dilution	Label	Freeze
1 ml vaginal secretion + 1 ml antibody mix<br>Vortex<br>take 0 min sample<br>Incubate at 37°C	0 min	100 µl sample + 900 µl buffer (2x)	VS1+MAB20<br>0 min, 37°C<br>dd/mm/yyyy	-70 °C
	15 min	100 µl sample + 900 µl buffer	VS1+MAB20<br>15 min, 37°C<br>dd/mm/yyyy	-70 °C
	30 min	100 µl sample + 900 µl buffer	VS1+MAB20<br>30 min, 37°C<br>dd/mm/yyyy	-70 °C
Vortex before taking each further aliquot!	60 min	100 µl sample + 900 µl buffer	VS1+MAB20<br>60 min, 37°C<br>dd/mm/yyyy	-70 °C
	120 min	100 µl sample + 900 µl buffer	VS1+MAB20<br>120 min, 37°C<br>dd/mm/yyyy	-70 °C
	240 min	100 µl sample + 900 µl buffer	VS1+MAB20<br>240 min, 37°C<br>dd/mm/yyyy	-70 °C
	480 min	100 µl sample + 900 µl buffer	VS1+MAB20<br>480 min, 37°C<br>dd/mm/yyyy	-70 °C
	24 h	100 µl sample + 900 µl buffer<br><br>1:10 dilution of remaining sample	VS1+MAB20<br>24 h, 37°C<br>dd/mm/yyyy<br>VS1+MAB20<br>24 h, 37°C<br>dd/mm/yyyy	-70 °C<br><br>-70°C

The information contained herein is the property of Polymun Scientific Immunbiologische Forschung GmbH and CONFIDENTIAL. The recipient is responsible for safe-keeping and prevention of unauthorised appropriation, use or disclosure.
